# Phase separation of Hippo signalling complexes

**DOI:** 10.15252/embj.2022112863

**Published:** 2023-02-20

**Authors:** Teresa T Bonello, Danfeng Cai, Georgina C Fletcher, Kyler Wiengartner, Victoria Pengilly, Kimberly S Lange, Zhe Liu, Jennifer Lippincott‐Schwartz, Jennifer M Kavran, Barry J Thompson

**Affiliations:** ^1^ EMBL Australia, John Curtin School of Medical Research Australian National University Canberra ACT Australia; ^2^ HHMI Janelia Research Campus Ashburn VA USA; ^3^ Department of Biochemistry and Molecular Biology Bloomberg School of Public Health Baltimore MD USA; ^4^ Epithelial Biology Laboratory The Francis Crick Institute London UK; ^5^ Department of Biophysics and Biophysical Chemistry, and Department of Oncology Johns Hopkins School of Medicine Baltimore MD USA

**Keywords:** condensates, epithelia, Hippo signalling, mechanobiology, Development, Signal Transduction

## Abstract

The Hippo pathway was originally discovered to control tissue growth in *Drosophila* and includes the Hippo kinase (Hpo; MST1/2 in mammals), scaffold protein Salvador (Sav; SAV1 in mammals) and the Warts kinase (Wts; LATS1/2 in mammals). The Hpo kinase is activated by binding to Crumbs‐Expanded (Crb‐Ex) and/or Merlin‐Kibra (Mer‐Kib) proteins at the apical domain of epithelial cells. Here we show that activation of Hpo also involves the formation of supramolecular complexes with properties of a biomolecular condensate, including concentration dependence and sensitivity to starvation, macromolecular crowding, or 1,6‐hexanediol treatment. Overexpressing Ex or Kib induces formation of micron‐scale Hpo condensates in the cytoplasm, rather than at the apical membrane. Several Hippo pathway components contain unstructured low‐complexity domains and purified Hpo‐Sav complexes undergo phase separation *in vitro*. Formation of Hpo condensates is conserved in human cells. We propose that apical Hpo kinase activation occurs in phase separated “signalosomes” induced by clustering of upstream pathway components.

## Graphical abstract

Mechanical strain and growth factor signalling regulate the condensation of Hippo pathway components in *Drosophila* and mammalian epithelia.

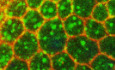

## Introduction

The Hippo signalling pathway was discovered in *Drosophila* to restrict the growth of proliferating epithelial tissues (Boggiano & Fehon, 2012; Irvine & Harvey, 2015; Karaman & Halder, 2018; Misra & Irvine, 2018; Davis & Tapon, 2019; Zheng & Pan, 2019) and has important roles in tissue regeneration and cancer (Moya & Halder, 2019; Dey *et al*, 2020; Thompson, 2020). The core components of the pathway include the upstream kinase Hippo (Hpo; MST1/2 in mammals; Harvey *et al*, 2003; Pantalacci *et al*, 2003; Udan *et al*, 2003; Wu *et al*, 2003), its adaptor protein Salvador (Sav; SAV1 in mammals; Kango‐Singh *et al*, 2002; Tapon *et al*, 2002), the downstream kinase Warts (Wts; LATS1/2 in mammals; Justice *et al*, 1995; Xu *et al*, 1995) and its adaptor protein Mats (MOB1 in mammals; Wei *et al*, 2007; Praskova *et al*, 2008).

Activation of the Hpo kinase involves binding to Sav and auto‐phosphorylation in its activation loop (Creasy *et al*, 1996; Praskova *et al*, 2004; Jin *et al*, 2012; Deng *et al*, 2013; Ni *et al*, 2013) and linker region (Ni *et al*, 2015) in order to bind Mats/MOB1 and activate Wts/LATS (Ni *et al*, 2015). Activated Wts then phosphorylates the nuclear transcriptional co‐activator Yorkie (Yki; YAP/TAZ in mammals) to retain it in the cytoplasm by inducing interaction with 14‐3‐3 proteins (Kanai *et al*, 2000; Huang *et al*, 2005; Zhao *et al*, 2007; Oh & Irvine, 2008, 2009). Hpo kinase auto‐phosphorylation can also be promoted by the Tao‐1 kinase (Boggiano *et al*, 2011; Poon *et al*, 2011) and can be antagonised by the STRIPAK PP2A phosphatase complex (Ribeiro *et al*, 2010; Zheng *et al*, 2017). Recent work revealed that the fundamental principle governing activation of the Hpo/MST kinase and auto‐phosphorylation is the physical proximity of the kinase domains (Tran *et al*, 2020). Thus, the biochemistry of the core Hpo‐Sav‐Wts‐Mats kinase complex and its ability to regulate Yki/YAP/TAZ are well understood, but the upstream signalling mechanisms that control activation of the Hpo kinase remain unclear.

Hpo kinase activation also depends upon several key upstream pathway components that localise to the apical plasma membrane of epithelial cells, including the Crumbs‐Expanded (Crb‐Ex) and Merlin‐Kibra (Mer‐Kib) complexes (McCartney *et al*, 2000; Hamaratoglu *et al*, 2006; Baumgartner *et al*, 2010; Chen *et al*, 2010; Genevet *et al*, 2010; Ling *et al*, 2010; Robinson *et al*, 2010; Yu *et al*, 2010), which interact with the spectrin cytoskeleton (Medina *et al*, 2002; Deng *et al*, 2015; Fletcher *et al*, 2015). Crb‐Ex complexes localise primarily in an apical‐lateral ring of cell–cell junctions (similar to vertebrate tight junctions), while Mer‐Kib complexes localise both to the apical‐lateral ring and to the medial‐apical region (Sun *et al*, 2015; Su *et al*, 2017). In addition, Sav is localised both to apical‐lateral cell–cell junctions (Yue *et al*, 2012) and to the medial‐apical region (Sun *et al*, 2015; Su *et al*, 2017). In contrast, Hpo is broadly distributed in the cytoplasm, although with some enrichment apically that overlaps with both apical‐lateral cell–cell junctions and the medial‐apical region (Sun *et al*, 2015; Su *et al*, 2017). Similarly, the bulk distribution of Wts is largely cytoplasmic, yet its signalling activity is strongly induced by recruitment to the apical plasma membrane (Yin *et al*, 2013).

Importantly, signal transduction can be activated simply by overexpression of the upstream activators Ex or Kib (Hamaratoglu *et al*, 2006; Baumgartner *et al*, 2010; Sun *et al*, 2015; Su *et al*, 2017). For example, increasing the level of Crb‐Ex complexes is sufficient to recruit Hpo kinase complexes (including Wts and Mats) to the apical‐lateral junctions (Sun *et al*, 2015), while increasing the level of Mer‐Kib complexes (by overexpression of Kib) recruits Hpo kinase complexes (including Sav and Wts) to the medial‐apical region (Su *et al*, 2017). Thus, apically localised upstream regulators are crucial for the activation of Hpo kinase complexes, yet precisely how they activate the Hpo kinase, and how this activation is physiologically regulated, needs further investigation.

Several different physiological stimuli can regulate the Hippo pathway in *Drosophila*, including mechanical, polarity and hormonal inputs. Mechanical strain (cell shape change) induced by stretching of the apical domain can dilute Crb‐Ex and Kib‐Mer complexes and reduce Hpo kinase activation (Fletcher *et al*, 2018). Mechanical stress (tension) arising from Rho‐mediated actomyosin contractility at E‐cadherin‐based adherens junctions can recruit the Ajuba protein to inhibit Wts activity (Rauskolb *et al*, 2014; Pan *et al*, 2016, 2018; Alegot *et al*, 2019). Fat‐Dachsous cadherins also planar polarise the Dachs myosin to increase junctional tension and inhibit Wts activity in certain tissues (Mao *et al*, 2006, 2011; Bosch *et al*, 2014; Rodrigues‐Campos & Thompson, 2014; Vrabioiu & Struhl, 2015). Finally, nutritionally induced circulating growth factors of the Insulin/IGF‐1 family signal via the PI3K‐Akt pathway to inhibit Hpo activity (Strassburger *et al*, 2012; Fan *et al*, 2013; Borreguero‐Munoz *et al*, 2019). Consequently, Hippo signalling is normally weakest in mechanically stretched epithelial cells and normally strongest in densely packed columnar epithelial cells – such as the ovarian follicular epithelium that surrounds the oocyte at stage 10 of oogenesis (Fletcher *et al*, 2018). In addition, Hpo signalling is further elevated upon nutrient deprivation (starvation) via reduced PI3K‐Akt signalling in follicle cells (Borreguero‐Munoz *et al*, 2019). Precisely how these different physiological inputs are integrated at the level of Hpo kinase activation remains poorly understood.

A common observation is that Hpo kinase complexes at the apical membrane often appear punctate in nature (Sun *et al*, 2015; Su *et al*, 2017; Fletcher *et al*, 2018), suggestive of higher order organisation rather than simple stochiometric complex formation, although the mechanistic basis for this appearance is not understood. One possibility is that Hpo kinase complexes may be localised to endosomal compartments adjacent to the apical membrane, as the upstream regulator Crb is a transmembrane protein that traffics through apically localised Rab11‐positive or Rab5‐positive endosomes (Blankenship *et al*, 2007; Roeth *et al*, 2009; Fletcher *et al*, 2012; Aguilar‐Aragon *et al*, 2020). An alternative possibility is that Hpo signalling complexes may undergo clustering and phase separation to form biomolecular condensates, as observed in other molecular systems (Hyman *et al*, 2014; Banani *et al*, 2017; Case *et al*, 2019). While phase separation is typically associated with cytoplasmic condensates, recent work identified a role for phase separation in transmembrane protein clustering (Banjade & Rosen, 2014; Su *et al*, 2016; Zeng *et al*, 2016; Case *et al*, 2019) and in formation of vertebrate tight junctions (Beutel *et al*, 2019; Schwayer *et al*, 2019), where many upstream Hippo components localise (Karaman & Halder, 2018). Furthermore, in the case of Wnt signalling, cytoplasmic condensates are recruited to the plasma membrane during signal transduction (Gammons & Bienz, 2018; Case *et al*, 2019; Schaefer & Peifer, 2019). Finally, the downstream nuclear transcriptional co‐activator YAP was also recently found to undergo phase‐separation in the nucleus and cytoplasm (Cai *et al*, 2019). Thus, phase separation of Hpo kinase complexes provides a plausible signalling mechanism.

Here we report that supramolecular assembly of Hpo kinase complexes can also occur by the formation of biomolecular condensates both *in vitro* and *in vivo*. The formation of Hpo condensates is strongly enhanced by starvation‐induced loss of PI3K‐Akt signalling, or by overexpression of the upstream components Ex and Kib – conditions that activate Hpo signalling. We further identify a key role for the mechanoresponsive spectrin cytoskeleton in promoting clustering of Crb‐Ex and Kib‐Mer complexes to induce Hpo kinase punctae formation, thereby rendering signal transduction sensitive to mechanical strain. Our findings lead us to propose clustering and phase separation as a unifying biophysical mechanism that enables integration of mechanics, polarity and nutrition by the Hpo signalling pathway.

## Results

### The Hpo kinase can form cytoplasmic condensates in *Drosophila*

**Figure 1:**
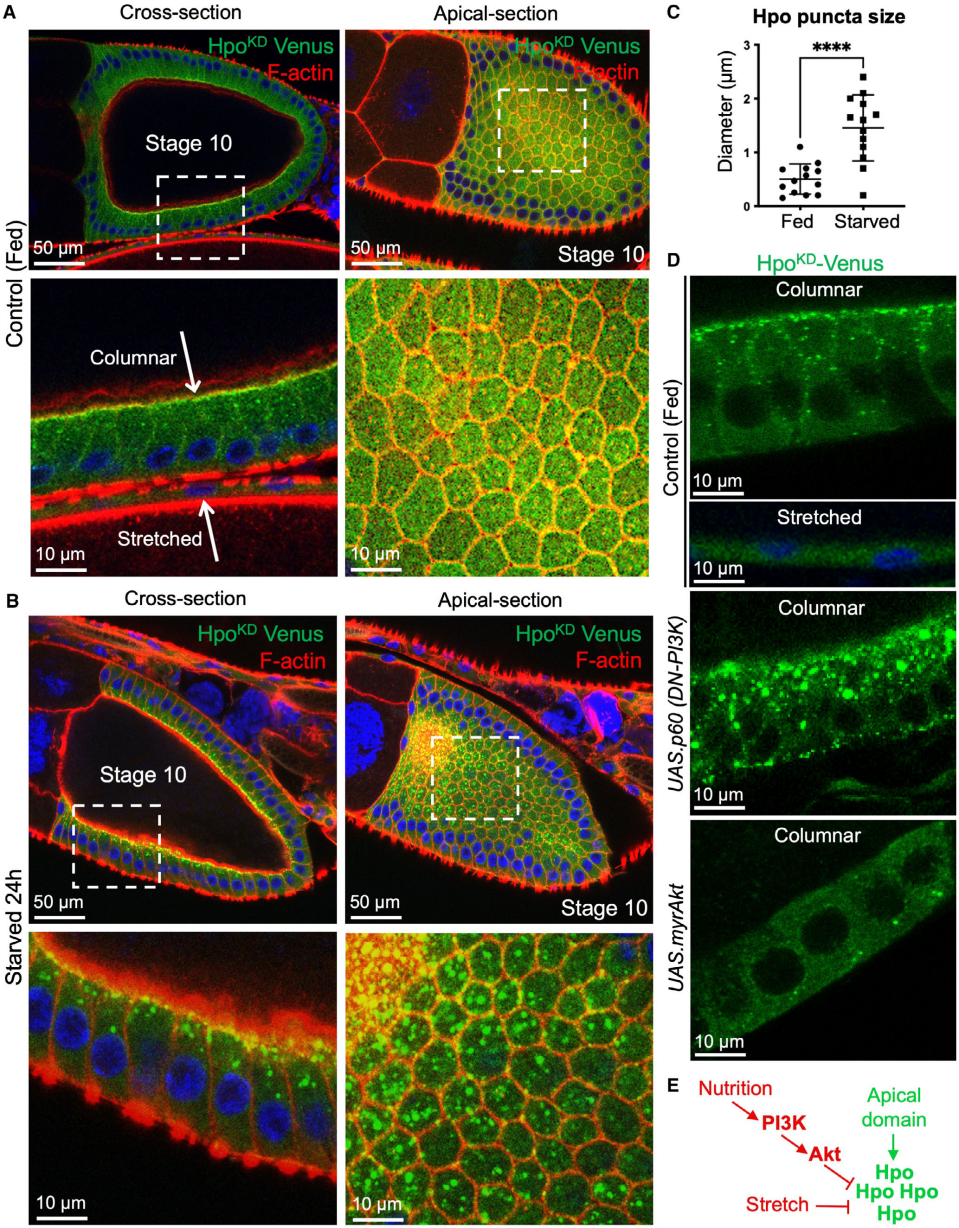
Hpo‐Venus can form cytoplasmic condensates in a mechanically‐ and nutritionally dependent manner *Drosophila* egg chambers are composed of a large oocyte surrounded by a columnar follicle cell epithelium that activates a Hpo kinase bimolecular fluorescence complementation (BiFC) dimerization sensor (HpoKD‐Venus; green) apically in small punctae. Note that the Hpo sensor is activated in columnar cells but not in mechanically stretched cells.Nutrient restriction (starvation) of adult females for 24 h causes formation of large micron‐scale Hpo sensor punctae.Quantification of the size of Hpo sensor punctae from (A) and (B). Biological replicates are plotted as individual data points (*n* = 13 fed; *n* = 13 starved), error bars represent one standard deviation from the mean, statistical significance was determined using a *t*‐test ****P < 0.0001 ***P < 0.001 **P < 0.01 *P < 0.05.Inhibition of PI3K by overexpression of a dominant‐negative version (the p60 subunit alone) is sufficient to induce formation of large micron‐scale Hpo sensor punctae, while activation of Akt by overexpression of a myristylated form of the protein is sufficient to reduce formation of the small apical Hpo sensor punctae that are normally observed in control animals.Schematic diagram of pathways regulating formation of supramolecular Hpo kinase punctae *in vivo*. Source data are available online for this figure.

**Figure EV1:**
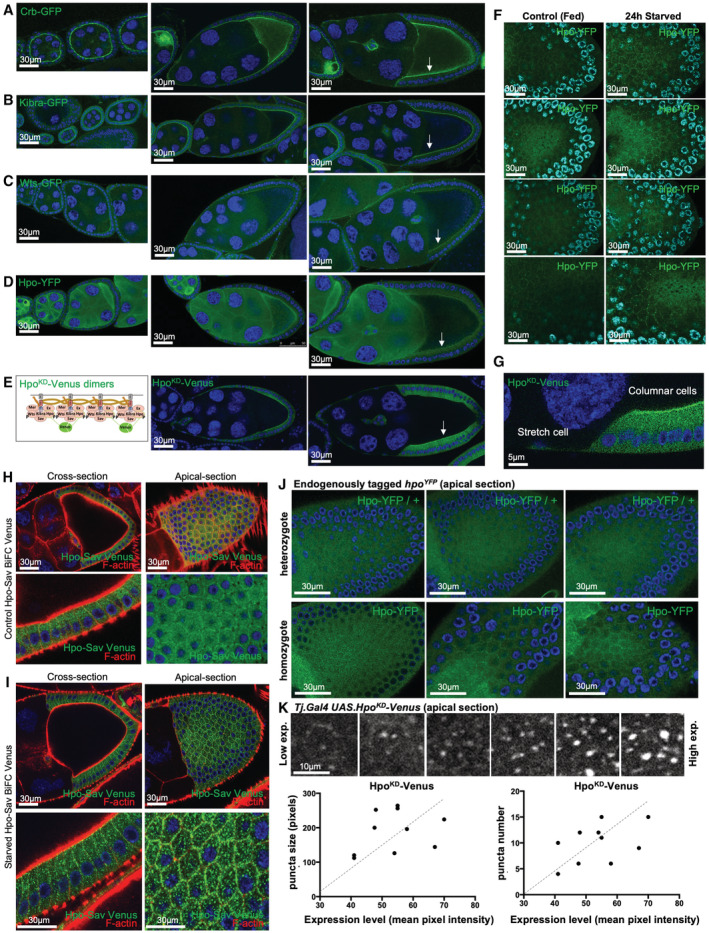
Phase separation of Hippo signalling complexes at the apical domain is enhanced by starvation or overexpression Crb‐GFP localises apically in cuboidal and columnar epithelial cells of the ovarian follicular epithelium.Kibra‐GFP localises apically in cuboidal and columnar epithelial cells of the ovarian follicular epithelium.Wts‐GFP localises apically in cuboidal and columnar epithelial cells of the ovarian follicular epithelium.Hpo‐YFP protein is mostly cytoplasmic, although weak apical signal is detectable in columnar epithelial cells.A HpoKD‐Venus dimerization sensor can be detected at the apical domain of densely packed columnar follicle cells, similar to Crb, Kib and Wts.Hpo‐YFP localises to the apical junctions in the follicular epithelium of control (fed) egg chambers and becomes strongly enriched into apical junction puncta under conditions of nutrient restriction.HpoKD‐Venus is apically enriched in columnar cells but not in stretch cells (high mag view of right‐hand panel in E).Hpo‐Sav Venus bimolecular fluorescence complementation (BiFC) sensor based on split‐Venus proteins fused to Hpo and Sav. Puncta formation in the cytoplasm is evident. Elevated signal at the apical domain is evident.Hpo‐Sav Venus BiFC sensor levels are elevated upon starvation for 24 h, producing an increase in puncta formation.Endogenously tagged Hpo‐YFP localises to apical punctae, which are more easily detected when the tagged allele is homozygous, rather than heterozygous.Ectopically expressed HpoKD‐Venus BiFC sensor forms larger puncta when expressed at higher levels. Quantification of puncta number and size is shown below. Biological replicates are plotted as individual data points. Source data are available online for this figure.

In the *Drosophila* ovarian follicle cell epithelium, Hpo signalling is physiologically activated in densely packed columnar follicle cells that surround the oocyte at stage 9–10 of oogenesis (Fletcher *et al*, 2018) and Hpo signal activation is further enhanced by nutrient deprivation (starvation) which reduces PI3K‐Akt signalling (Strassburger *et al*, 2012; Borreguero‐Munoz *et al*, 2019). We sought to visualise Hpo kinase activation in this model system using both an endogenously tagged Hpo‐YFP knockin allele, to detect bulk Hpo protein, as well as a split‐Venus bimolecular fluorescence complementation (BiFC) reporter, in which dimers/multimers of Hpo reconstitute the Venus molecule to induce fluorescence, to detect active Hpo (Fletcher *et al*, 2018). A kinase‐dead version of this BiFC reporter (Hpo^KD^‐Venus) can be expressed without inducing ectopic Hpo signalling, avoiding abnormal phenotypes (Fletcher *et al*, 2018). Hpo^KD^‐Venus complexes are most strongly detectable at the apical plasma membrane of densely packed columnar follicle cells at stage 10 of oogenesis, similar to other upstream Hippo pathway components (Figs 1A and EV1A–E; Fletcher *et al*, 2018). Hpo^KD^‐Venus complexes exhibit a punctate appearance at the apical membrane (Fig 1A), which we previously showed overlaps with both Crb‐Ex and Mer‐Kib complexes in follicle cells (Sherrard & Fehon, 2015; Fletcher *et al*, 2018).

**Figure EV2:**
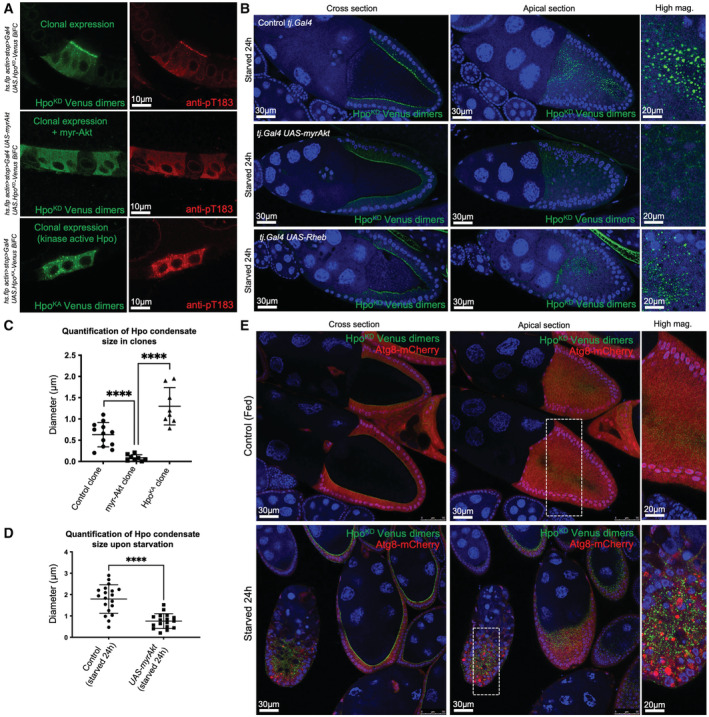
Hpo‐Venus dimers are phosphorylated at Thr183 and condensate formation is inhibited by ectopic expression of active Akt but not Rheb Clonal expression of HpoKD‐Venus dimers stain positively for phospho‐Thr183, indicating phosphorylation of the kinase activation loop. Clonal expression of HpoKD‐Venus dimers fails to enrich apically in the follicular epithelium when co‐expressed with constitutively active Akt. Clonal expression of active Hpo‐Venus dimers localises to large puncta within the cytoplasm which stain positive for phospho Thr183.Hpo punctae induced by nutrient restriction are diminished by constitutively active Akt signalling in the follicular epithelium, but not by expression of Rheb (which activates TOR).Quantification of Hpo punctae size in the clonal conditions described in (A). Biological replicates are plotted as individual data points (*n* > 8 per sample), error bars represent one standard deviation from the mean, statistical significance was determined using a *t*‐test ****P < 0.0001 ***P < 0.001 **P < 0.01 *P < 0.05.Quantification of Hpo punctae size under nutrient restriction in the presence or absence of constitutively active Akt signalling. Biological replicates are plotted as individual data points (*n* > 15 per sample), error bars represent one standard deviation from the mean, statistical significance was determined using a *t*‐test ****P < 0.0001 ***P < 0.001 **P < 0.01 *P < 0.05.Minimal autophagic activity is observed in the follicular epithelium of egg chambers from well‐fed females. Nutrient restriction promotes a strong autophagic response in the follicular epithelium. The autophagosome compartment, marked my mCherry‐Atg8a, does not colocalise with Hpo puncta. Source data are available online for this figure.

Upon nutrient restriction, or reduced PI3K‐Akt signalling, we noticed the striking appearance of large spherical Hpo^KD^‐Venus supramolecular complexes both at the apical membrane and in the cytoplasm (Fig 1A–E; Borreguero‐Munoz *et al*, 2019). We confirmed these findings using the endogenously tagged Hpo‐YFP reporter line as well as an alternative Hpo‐Sav split‐Venus BiFC interaction sensor (Fig EV1F–I). Upon starvation, the endogenous Hpo‐YFP forms punctae and formation of these supramolecular complexes is concentration‐dependent (Fig EV1J and K). The Hpo^KD^‐Venus complexes are phosphorylated in their activation loop (anti‐pT195, equivalent to pT183 in MST1), confirming that they are phosphorylated by endogenous Hpo kinase (Fig EV2A). Furthermore, expression of a kinase‐active version of the Hpo‐Venus dimerization sensor, which induces signalling, is sufficient to induce large spherical complexes in the cytoplasm even in normally fed animals (Fig EV2A). Overexpression of constitutively active myr‐Akt reduced the formation of Hpo^KD^‐Venus complexes (Figs 1D and E, and EV2A–D). These findings show that starvation‐induced loss of PI3K‐Akt signalling can promote formation of large Hpo kinase complexes, while activation of PI3K‐Akt signalling reduces their formation.

**Figure EV3:**
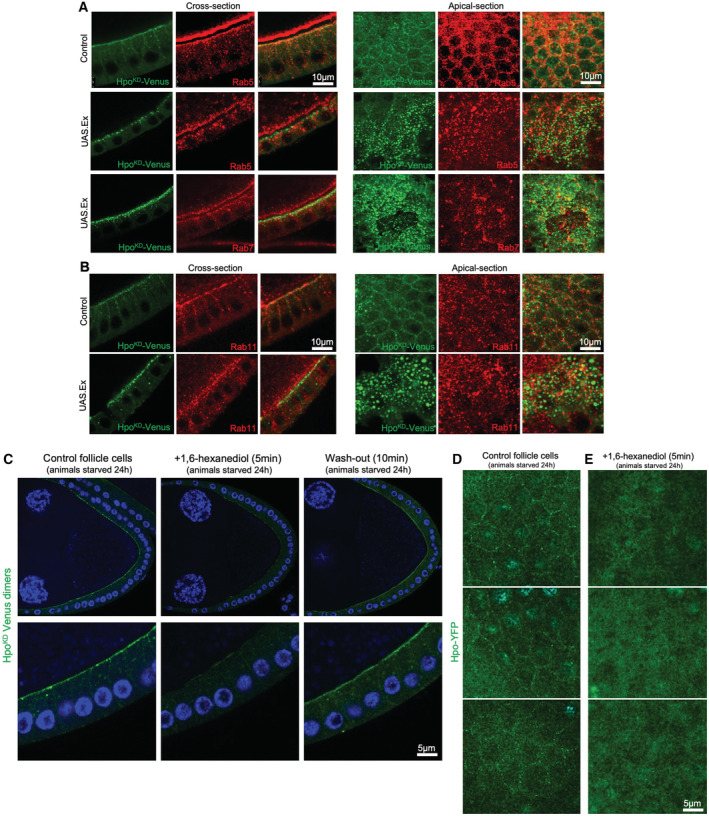
Hpo kinase condensates do not co‐localise with endosomal markers and are reversibly sensitive to 1,6‐hexanediol treatment AHpo puncta formed at the apical domain of the follicular epithelium under wild‐type conditions or strongly recruited into puncta by Ex overexpression, do not co‐localise with markers for early (Rab5) or late (Rab7) endosomal compartments.BHpo puncta formed at the apical domain of the follicular epithelium under wild‐type conditions or Ex overexpression, do not co‐localise with Rab11, a marker of recycling endosomes.C1,6‐hexanediol treatment reversibly disperses HpoKD‐Venus apical clusters.D, E1,6‐hexanediol treatment also disperses endogenous Hpo‐YFP clusters that appear under starvation. Source data are available online for this figure.

To characterise the biophysical nature of these Hpo kinase complexes, we first considered the possibility that they may be bound to autophagosomes or other endosomes. Starvation, or loss of PI3K‐Akt signalling, is known to cause reduced TORC1 activity that drives the formation of autophagosomes via activation of the kinase Atg1 (ULK1 in mammals; Noda & Ohsumi, 1998; Arico *et al*, 2001; Scott *et al*, 2004, 2007). To examine this possibility, we made use of an Atg8‐mCherry sensor that localises strongly to autophagosomes (Mauvezin *et al*, 2014). We find that the formation of large Hpo^KD^‐Venus complexes appears to precede formation of large autophagosomes in ovarian follicle cells following starvation of adult females, and even when large autophagosomes do form in severely affected egg chambers, they rarely overlap with Hpo kinase complexes (Fig EV2E). Similarly, endosomal markers do not colocalise with Hpo kinase complexes (Fig EV3A and B). These results indicate that large Hpo kinase complexes induced upon nutrient restriction, or reduced PI3K‐Akt signalling, are generally distinct from both autophagosomes and endosomes.

**Figure 2:**
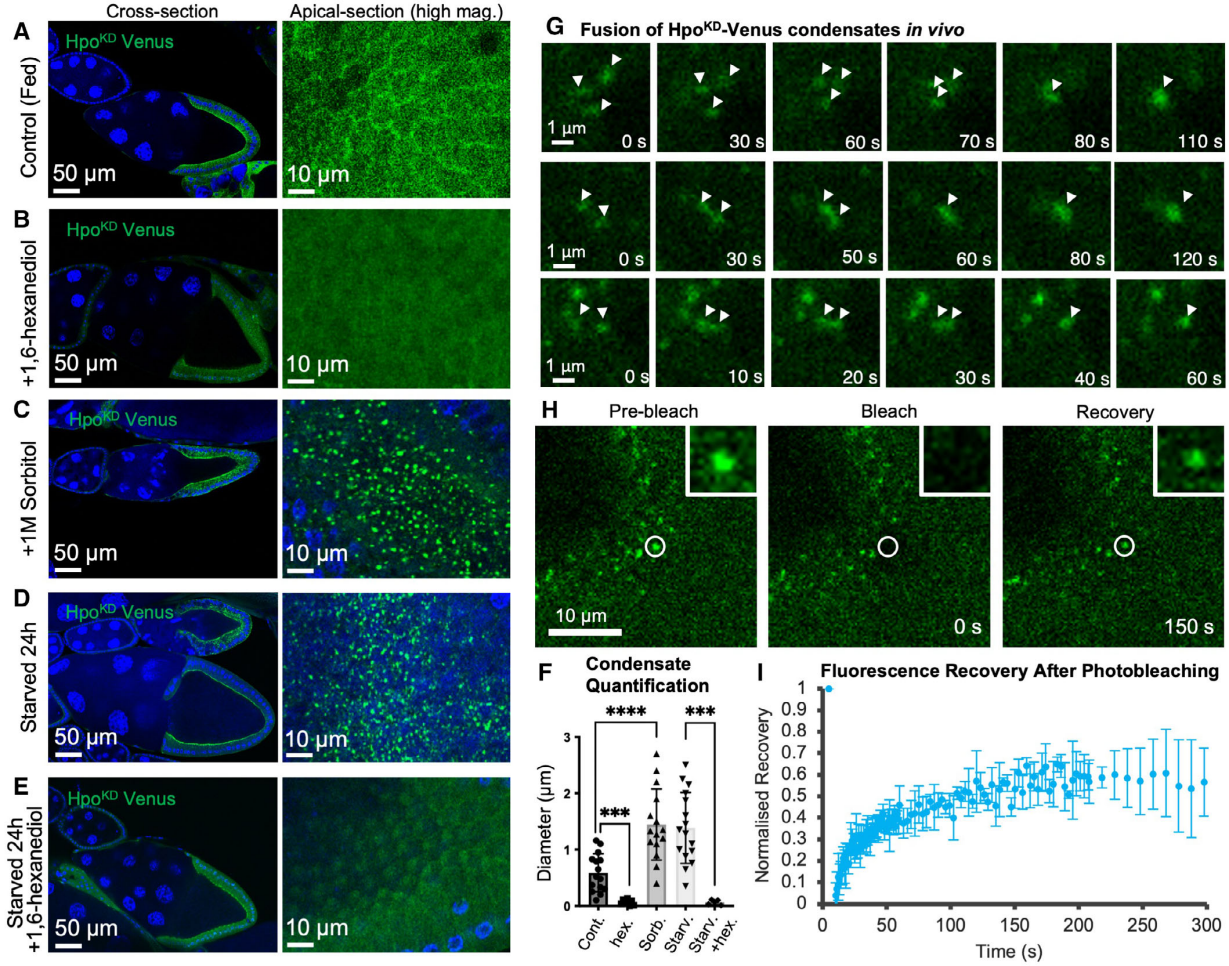
Hpo‐Venus punctae are sensitive to 1,6‐hexanediol, enhanced by macromolecular crowding, and undergo fusion and dynamic exchange with the cytoplasm *Drosophila* egg chambers at stages 9/10 of oogenesis expressing a Hpo kinase dimerization sensor that forms small punctae at the apical domain of the follicle cell epithelium that surrounds the oocyte.Treatment of egg chambers with 1,6‐hexanediol (10%) for 5 min abolishes formation of Hpo sensor punctae.Treatment of egg chambers with 1 M Sorbitol to osmotically withdraw water, and thereby induce macromolecular crowding, is sufficient to induce formation of large micron‐scale Hpo sensor punctae.Nutrient restriction (starvation) of adult females for 24 h induces large micron‐scale Hpo sensor punctae that are similar in size to those in (C).Treatment of egg chambers from adult females starved for 24 h with 1,6‐hexanediol (10%) for 5 min abolishes formation of Hpo sensor punctae.Quantification of the size of Hpo sensor punctae from (A–E). Biological replicates are plotted as individual data points (*n* = 15 control; *n* = 8 hex; *n* = 15 sorb; *n* = 16 starv; *n* = 5 starv + hex), error bars represent one standard deviation from the mean, statistical significance was determined using a *t*‐test ****P < 0.0001 ***P < 0.001 **P < 0.01 *P < 0.05.Live‐imaging of HpoKD‐Venus (green) condensates within follicle cells at high magnification reveals fusion events, occurring over the timescale of seconds. White arrowheads point to individual punctae undergoing fusion.Fluorescence Recovery After Photobleaching (FRAP) experiments reveal that photobleaching of individual condensates is followed by rapid recovery after 150 s.Quantification of FRAP recovery as a fraction of the initial fluorescence intensity. The rapid initial slope of recovery suggests dynamic exchange between the condensate and the cytoplasmic pool of the proteins. Temporal averages are plotted as individual data points (*n* > 3 biological replicates), error bars represent one standard deviation from the mean. Source data are available online for this figure.

We next considered the possibility that large Hpo kinase complexes may instead form by phase separation into biomolecular condensates (Hyman *et al*, 2014; Banani *et al*, 2017; Shin & Brangwynne, 2017; Case *et al*, 2019). Starvation of cells is known to induce phase separation of various condensates in the cytoplasm (van Leeuwen & Rabouille, 2019). Typically, biomolecular condensates can be disrupted by treatment with the compound 1,6‐hexanediol, which disrupts phase separation, and enhanced by macromolecular crowding induced by the osmotic withdrawal of water from cells upon treatment with PEG or Sorbitol (Cai *et al*, 2019; McSwiggen *et al*, 2019). Accordingly, we find that treatment of egg chambers with 10% 1,6‐hexanediol for 5 min strongly inhibits Hpo^KD^‐Venus punctae from coalescing at the apical membrane of follicle cells (Fig 2A and B). Conversely, treatment of egg chambers with 1 M Sorbitol to induce macromolecular crowding was sufficient to drive the formation of large spherical Hpo^KD^‐Venus complexes, even in well fed animals (Fig 2C). Crowding‐induced Hpo^KD^‐Venus complexes are similar in size and number to those induced by starvation of adult females (Fig 2D). Furthermore, the large starvation‐induced Hpo^KD^‐Venus complexes do not form in the presence of 10% 1,6‐hexanediol (Figs 2E and F, and EV3C). These findings suggest that Hpo kinase punctae display several properties of biomolecular condensates, including phase separation driven by increasing protein concentrations and sensitivity to crowding/hexanediol treatment.

Two further properties of biomolecular condensates include the ability of two condensates to undergo dynamic fusion within the cytoplasm and to exhibit dynamic exchange of proteins between the condensate and the cytoplasm. To test these two key properties, we performed live imaging of Hpo^KD^‐Venus punctae formed under starvation conditions in *Drosophila* follicular epithelium. We observed multiple fusion events between two or three punctae in the cytoplasm of follicle cells over a time‐scale of seconds (Fig 2G). In addition, we performed photobleaching of Hpo^KD^‐Venus punctae and measured the recovery of fluorescence by dynamic exchange of protein from the surrounding cytoplasm (Fig 2H). Quantification of fluorescence recovery after photobleaching (FRAP) indicates rapid recovery of punctae over a time‐scale of seconds (Fig 2I). Note that FRAP recovery is not able to return to full fluorescence intensity, and this might reflect generalised mobility of the punctae in the *z*‐axis that we observed during live‐imaging (Fig 2I). Together, these results confirm that Hpo punctae share two key characteristics of biomolecular condensates: dynamic fusion events and dynamic exchange with the cytoplasm.

**Figure 3:**
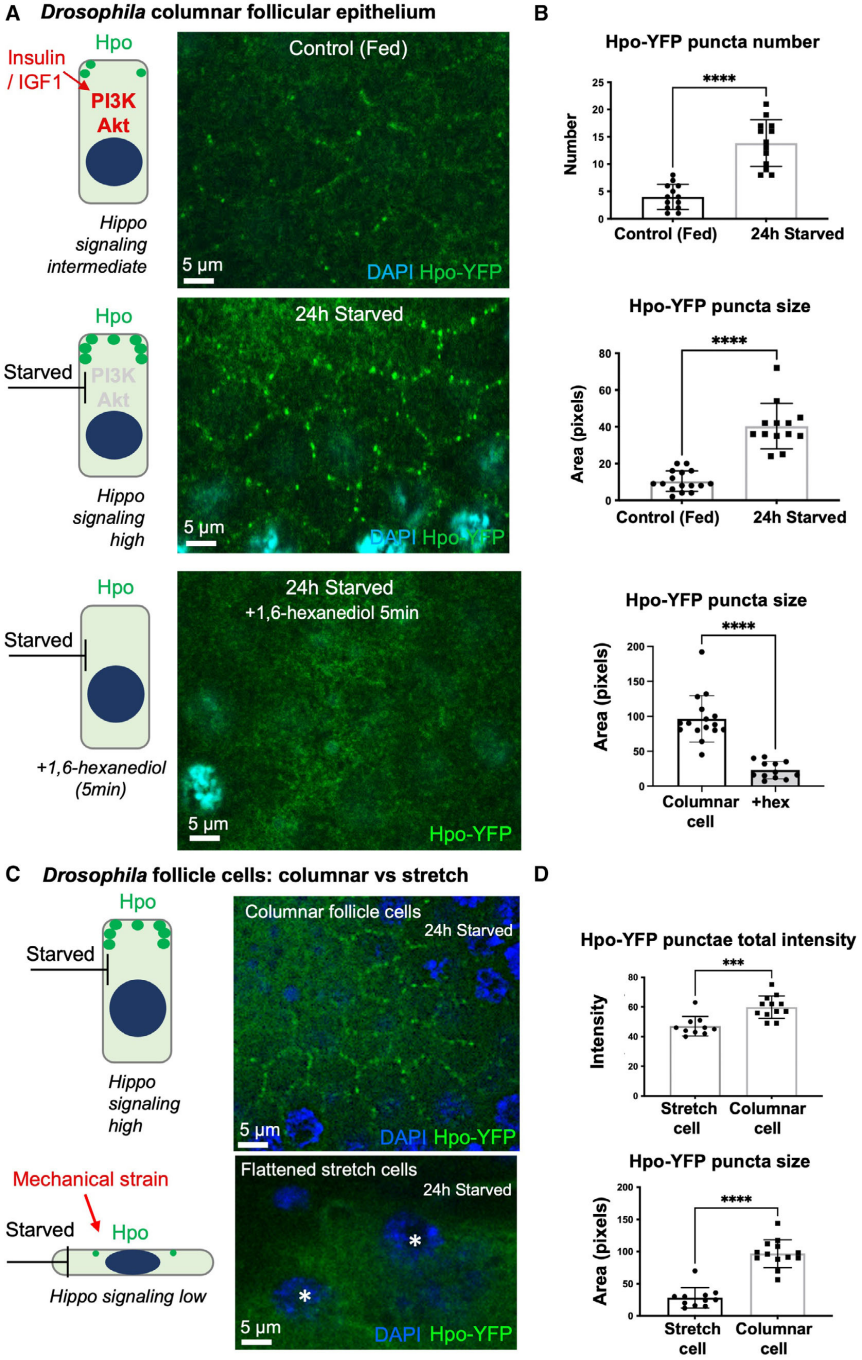
Endogenous Hpo‐YFP punctae are regulated by nutritional and mechanical stimuli Apical cross‐sections of *Drosophila* ovarian follicle cell epithelia at high magnification, showing accumulation of Hpo‐YFP punctae at the apical domain, which is strongly enhanced upon 24 h starvation to reduce Insulin/IGF‐1 signalling. Hpo puncta are lost following a short treatment with 1,6‐hexanediol. Samples were fixed before imaging.Quantification of Hpo‐YFP puncta number and size in (A). Biological replicates are plotted as individual data points (*n* > 13 for all samples), error bars represent one standard deviation from the mean, statistical significance was determined using a *t*‐test ****P < 0.0001 ***P < 0.001 **P < 0.01 *P < 0.05.Apical cross‐sections of *Drosophila* ovarian follicle cell epithelia at high magnification, showing loss of Hpo‐YFP punctae upon mechanical strain in “stretch cells”. The *Drosophila* follicle cell epithelium at stage 10 is composed of anterior stretch cells (flat) and columnar cells. Endogenously tagged Hpo punctae are strongly enriched at the apical domain of columnar cells but not in flattened stretch cells. White asterisks demarcate nuclei of individual stretch cells.Quantification of Hpo‐YFP total intensity and size in (C). Biological replicates are plotted as individual data points (*n* > 10 for all samples), error bars represent one standard deviation from the mean, statistical significance was determined using a *t*‐test ****P < 0.0001 ***P < 0.001 **P < 0.01 *P < 0.05. Source data are available online for this figure.

Endogenously tagged Hpo‐YFP also forms puncta apically (Fig 3A), where upstream signalling components are known to localise. Furthermore, puncta formation by endogenous Hpo‐YFP is also regulated by both nutritional and mechanical inputs, as well as being sensitive to treatment with 1,6‐hexanediol (Figs 3A–D, and EV3D and E). These results provide further support for the notion that phase separation of endogenous Hpo signalling complexes is a physiological signalling mechanism induced by upstream apical cues in *Drosophila*.

### Hpo kinase condensates can be nucleated by upstream pathway components in *Drosophila*

**Figure 4:**
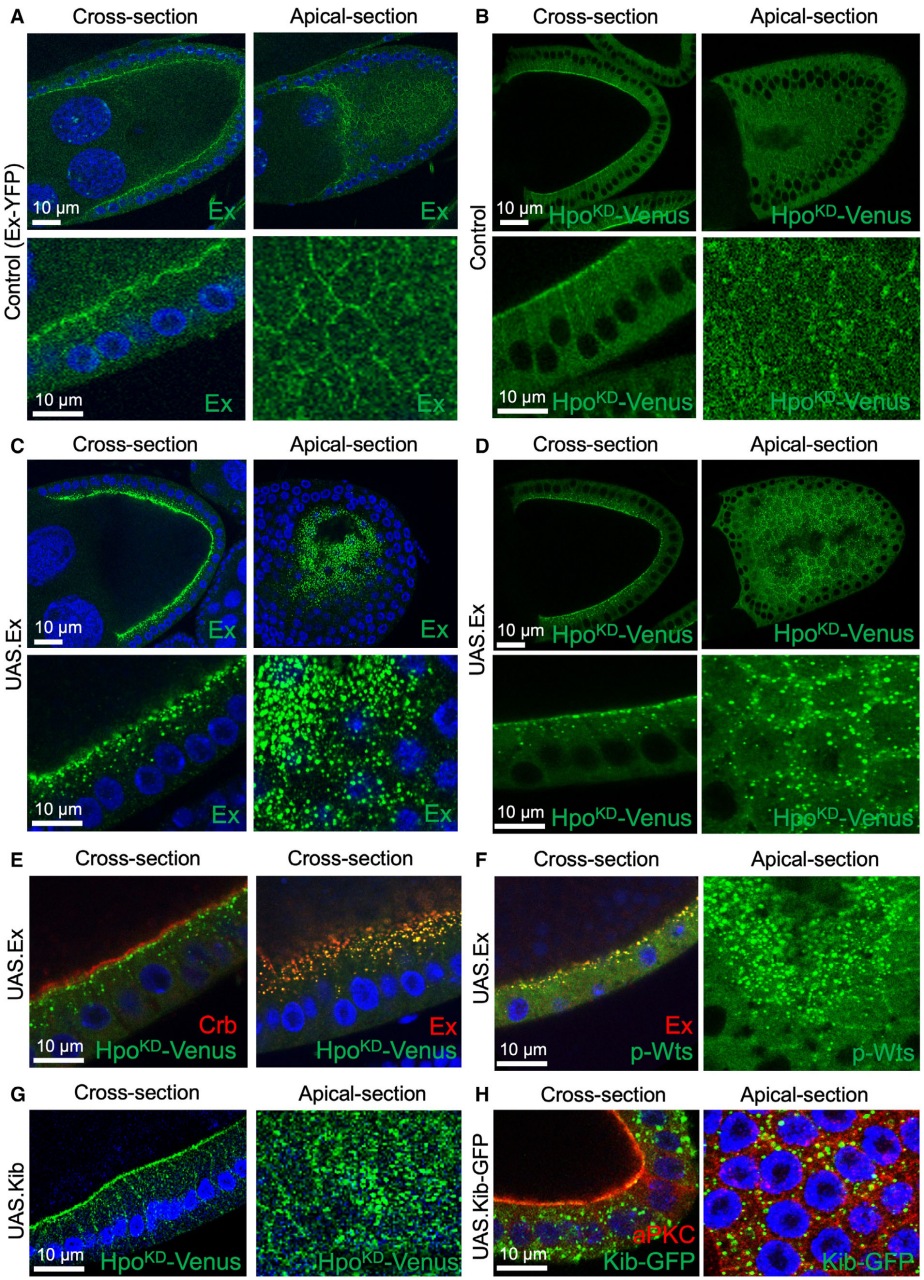
Expanded & Kibra overexpression induces formation of large Hpo punctae in the cytoplasm Expanded (Ex; endogenously tagged with YFP) localises to the apical domain of columnar follicle cells at stages 9/10 of oogenesis.The Hpo dimerization sensor localises in a similar pattern to Ex.Overexpression of Ex is sufficient to induce formation of large micron‐scale condensates in the cytoplasm.Overexpression of Ex is sufficient to recruit the Hpo sensor into large micron‐scale condensates in the cytoplasm.Condensates induced by high levels of Ex in the cytoplasm co‐localise with the Hpo sensor but do not co‐localise with Crb, which remains apical.Condensates induced by high levels of Ex in the cytoplasm immunostain positively for phosphorylated Wts.Moderate overexpression of untagged Kib is sufficient to induce the formation of large micron‐scale Hpo^KD^‐Venus condensates that primarily localise apically. DAPI marks nuclei (blue).Overexpression of Kib‐GFP with the Gal4/UAS system is sufficient to induce formation of large micron‐scale cytoplasmic condensates. Source data are available online for this figure.

A fourth key property of biomolecular condensates is their ability to be “nucleated” by regulatory proteins in a concentration‐dependent manner (Hyman *et al*, 2014; Banani *et al*, 2017). Since Hpo signalling can be induced simply by raising the expression level of the upstream regulators Ex or Kib, we tested whether overexpression of these proteins would be sufficient to induce Hpo kinase punctae in the cytoplasm. In the case of Ex, we find that endogenously tagged Ex‐YFP exhibits a similar or overlapping punctate pattern to that formed by Hpo^KD^‐Venus punctae at the apical membrane domain (Fig 4A and B) and that Ex overexpression is sufficient to drive formation of large spherical Ex and Hpo^KD^‐Venus punctae in the cytoplasm (Fig 4C and D). The Ex and Hpo^KD^‐Venus proteins co‐localise in the same punctae in the cytoplasm, which do not contain the Crb transmembrane protein, which largely remains at the apical membrane (Fig 4E). Furthermore, the Ex‐induced Hpo^KD^‐Venus complexes do not co‐localise with endosomes through which Crb is known to traffic, such as Rab5, Rab7, or Rab11 (Fig EV3A). The Ex‐induced punctae also stain positively for active phosphorylated Wts/Lats (p‐Wts), consistent with the notion that they are actively signalling via recruitment of the endogenous Hpo kinase (Fig 4F). In the case of Kib, we find that overexpression of Kib‐GFP was sufficient to induce large spherical punctae of Hpo^KD^‐Venus and Kib‐GFP itself (Fig 4G and H). These results suggest that Ex or Kib overexpression is sufficient to promote formation of active Hpo kinase condensates.

**Figure EV4:**
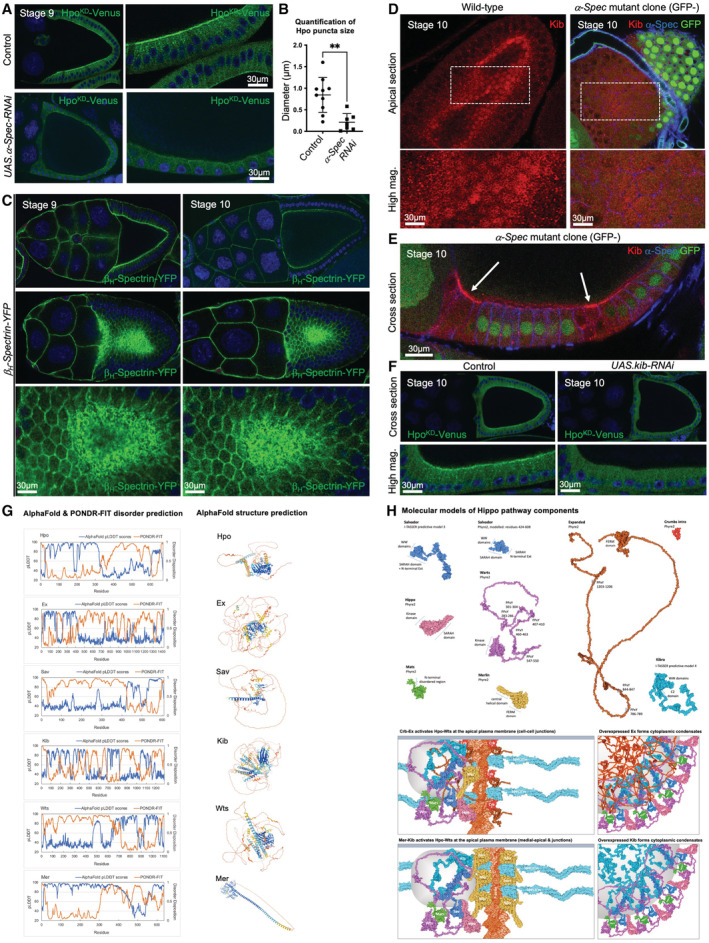
The spectrin cytoskeleton promotes assembly of Hippo signalling condensates via local clustering at the apical plasma membrane α‐Spectrin is required for active Hpo signalling at the apical domain of follicular epithelial cells.Loss of α‐Spectrin significantly reduces Hpo puncta size. Quantification of Hpo punctae size in the presence or absence of α‐Spectrin‐RNAi. Biological replicates are plotted as individual data points (*n* > 6 per sample), error bars represent one standard deviation from the mean, statistical significance was determined using a *t*‐test ****P < 0.0001 ***P < 0.001 **P < 0.01 *P < 0.05.Endogenously tagged β_H_‐Spectrin forms a dense mesh at the apical domain of follicular epithelial cells in stage 9/10 egg chambers.Kibra localises predominantly to the medial‐apical region of follicular epithelial cells, and this localisation is dependent on α‐Spectrin.Mislocalisation of Kibra in α‐Spectrin clones is not a consequence of reduced Kibra levels at the apical domain.Kibra is required for active Hpo signalling at the apical domain of follicular epithelial cells.Disorder prediction of *Drosophila* Hpo pathway proteins, using two independent algorithms. For meta‐predictor PONDR‐FIT, residues with a score above 0.5 are predicted disordered. The per‐residue confidence score (pLDDT) generated by AlphaFold predicts regions below 50 pLDDT to be unstructured in isolation. AlphaFold structure prediction models for Hpo pathway proteins, colour‐coded according to model confidence. Orange regions correspond to very low model confidence (pLDDT < 50), predicted to represent intrinsically disordered protein sequence.Molecular modelling of *Drosophila* Hpo components using the protein structure prediction server Phyre2. Where protein structures were incomplete or could not be predicted (e.g. Sav, Kib), protein sequences were submitted to I‐TASSER. The PDB outputs from both servers were uploaded to Illustrate (https://ccsb.scripps.edu/illustrate/) to generate the graphics. Predictive reconstructions of the Crbs‐Ex complexes at the apical junctions and Kib‐Mer complexes at the medial‐apical domain, highlighting the contribution of intrinsically disordered protein structures to both networks. Ex and Kib nucleate Hpo condensate formation *in vivo*, a process which may be driven by their intrinsically disordered domains. Source data are available online for this figure.

Crb, Ex, Kib and Mer interact with the spectrin cytoskeleton, which forms a spring‐like mesh that links F‐actin to the plasma membrane, to regulate Hpo signalling in *Drosophila* (Medina *et al*, 2002; Deng *et al*, 2015, 2020; Fletcher *et al*, 2015). Accordingly, we find that silencing of *alpha‐Spectrin* (*α‐Spec*) expression by *tj.Gal4*‐driven *UAS.α‐Spec RNAi* strongly reduces apical localisation of Hpo^KD^‐Venus complexes (Fig EV4A and B). The localisation of the apical spectrin cytoskeleton can be readily visualised with a YFP‐tagged betaHeavy‐Spectrin (ß_H_‐Spec‐YFP) allele (Fig EV4C). In addition, mutant clones of *α‐Spec* induced in columnar follicle cells at stage 10 cause a loss of Kib clusters from the medial‐apical region (Fig EV4D). This loss of Kib clustering is not due to reduced Kib levels, which are actually transcriptionally induced as a consequence of the loss of Hpo signalling that occurs in *α‐Spec* mutant clones (Fig EV4E). Furthermore, silencing of *kib* itself via *tj.Gal4*‐driven *UAS.Kib RNAi* strongly reduces apical Hpo^KD^‐Venus punctae (Fig EV4F). These findings further validate the Hpo^KD^‐Venus sensor as a physiological readout of Hippo signalling whose activity depends strictly on endogenous upstream Hippo pathway components. In addition, these results demonstrate an important role for the spectrin cytoskeleton in regulating clustering of Hpo kinase complexes by apical Crb‐Ex and Mer‐Kib to induce signalling.

**Figure 5:**
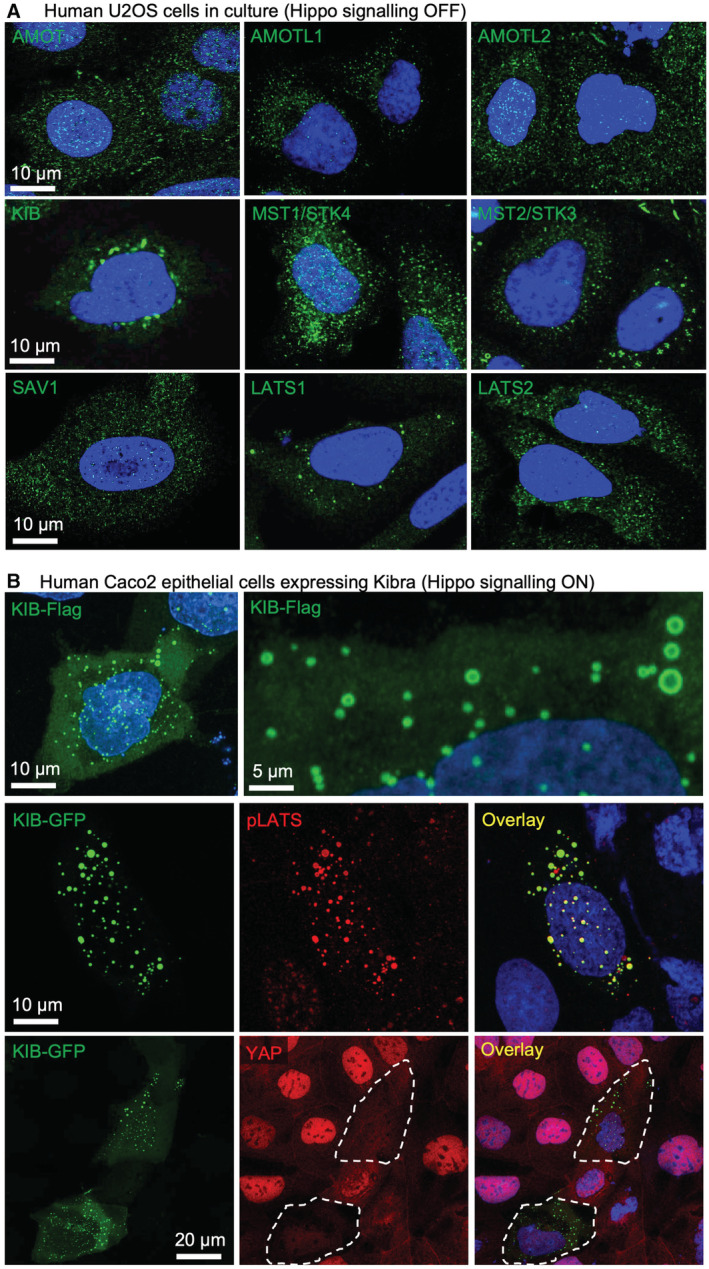
Human Kibra overexpression induces formation of large pLATS‐containing punctae in the cytoplasm and induces signalling Human U2OS cells in culture immunostained for endogenous Hippo pathway components reveals punctate subcellular localisation within the cytoplasm.Ectopic expression of Flag‐tagged human Kibra (KIB‐Flag) generates large spherical punctae in the cytoplasm of human Caco2 epithelial cells (top). Co‐localisation of KIB‐GFP with active phosphorylated LATS (pLATS) in the cytoplasm of human Caco2 epithelial cells (middle). Note that cells expressing KIB‐GFP have increased Hippo signalling as indicated by reduced levels of nuclear YAP localisation (bottom). Dashed lines demarcate transfected cells. Source data are available online for this figure.

The major upstream Hpo pathway components all contain intrinsically disordered regions, identified using two independent algorithms for disorder prediction (Fig EV4G). Molecular modelling of these proteins suggests that such unstructured regions could engage in multivalent interactions, in part by the presence of multiple PPXY motifs that mediate PPXY‐WW interaction networks (Fig EV4H). Intrinsically disordered protein regions are characteristic of both the Crb‐Ex network at apical junctions and the Mer‐Kib complexes at the medial‐apical region/microvilli and at apical junctions (Fig EV4H). Notably, the long intrinsically disordered regions in both Ex and Kib may enable these proteins to induce condensate formation in the cytoplasm when overexpressed (Fig EV4H). Thus, the key upstream regulators of Hippo signalling can promote the formation of large Hpo kinase punctae as well as inducing signal transduction, suggesting that these two properties may be linked.

### Hpo/MST kinase condensates can be nucleated by upstream pathway components in human cells

**Figure 6:**
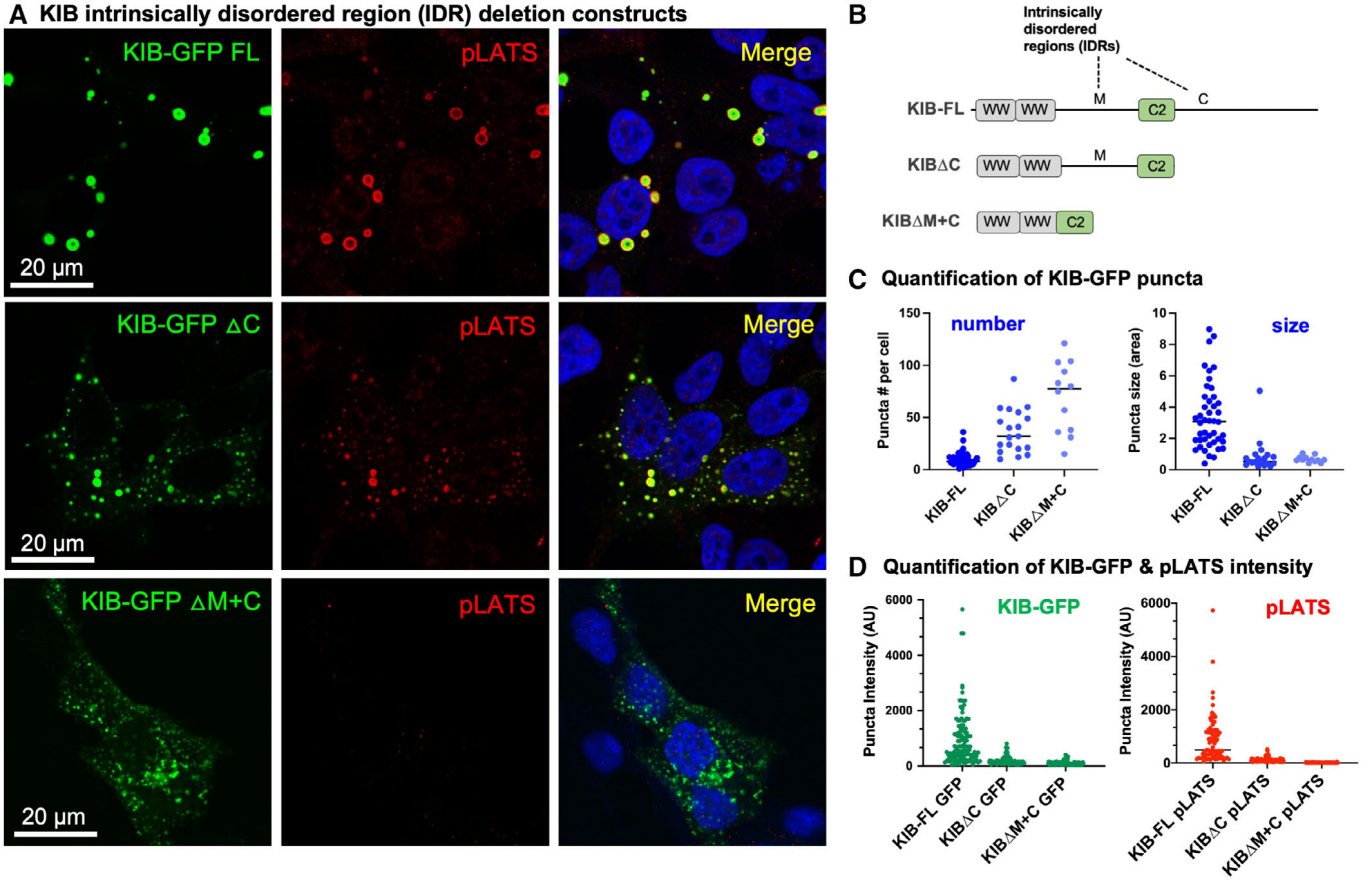
Intrinsically disordered regions (IDRs) are required for KIB to form large spherical punctae and to activate pLATS Expression of KIB‐GFP, KIB∆C‐GFP or KIB∆M + C‐GFP in human HEK293T cells and co‐staining for pLATS. DAPI marks nuclei in blue.Schematic diagram of KIB intrinsically disordered regions (M) and (C) and the ∆C and ∆M + C deletion constructs.Quantification of KIB‐GFP puncta number and size showing dependence upon the IDRs. Biological replicates are plotted as individual data points (*n* > 12 for all samples), error bars represent one standard deviation from the mean, statistical significance was determined using a *t*‐test ****P < 0.0001 ***P < 0.001 **P < 0.01 *P < 0.05.Quantification of KIB‐GFP and pLATS intensity showing dependence of both condensate formation and signalling upon the IDRs. Biological replicates are plotted as individual data points (*n* > 40 for all samples), error bars represent one standard deviation from the mean, and statistical significance was determined using a *t*‐test ****P < 0.0001 ***P < 0.001 **P < 0.01 *P < 0.05. Source data are available online for this figure.

**Figure EV5:**
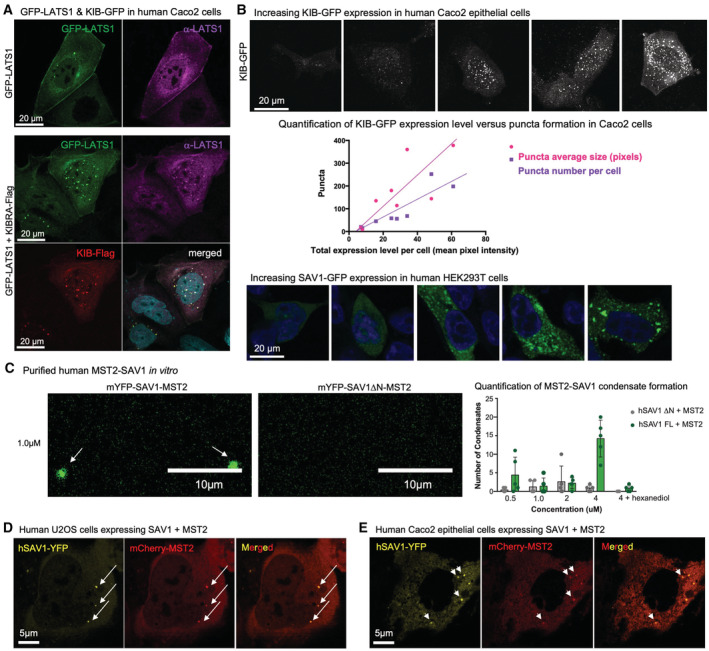
Phase separation of mammalian Hippo signalling condensates *in vitro* and *in vivo* GFP‐LATS1 forms cytoplasmic punctae when expressed in Caco2 epithelial cells. KIB‐Flag colocalises with GFP‐LATS1 in cytoplasmic punctae when co‐expressed in Caco2 epithelial cells.Ectopic KIB‐GFP expression in human Caco2 epithelial cells causes formation of punctae in the cytoplasm. Quantification of KIB puncta size and number reveals a linear correlation with the total expression level of KIB in the cell. Ectopic SAV1‐GFP expression in human Caco2 epithelial cells causes the formation of punctae in the cytoplasm.Purified mYFP‐SAV1:MST2 complex undergoes phase separation to form distinct puncta in a concentration‐dependent manner. Additionally, fewer condensates are observed for complexes containing a variant of SAV1 that lacks the predicted disordered regions, or upon treatment with 1,6‐hexanediol. Biological replicates are plotted as individual data points (*n* > 4 per sample), error bars represent one standard deviation from the mean, statistical significance was determined using a *t*‐test ****P < 0.0001 ***P < 0.001 **P < 0.01 *P < 0.05.In U‐2 OS cells, expression of monomeric YFP‐tagged SAV1 together with mCherry‐tagged MST2 was sufficient to form condensates in which both proteins co‐localise.In Caco‐2 epithelial cells, expression of monomeric YFP‐tagged Sav1 together with mCherry‐tagged MST2 was sufficient to form condensates in which both proteins co‐localise. Source data are available online for this figure.

Endogenous human Hpo pathway components are typically found in very small cytoplasmic puncta by immunostaining in flattened human cells in culture, which reflects a cellular state in which signalling activity is low (Fig 5A). Activation of signalling by transfecting Caco2 epithelial cells with upstream apical Hpo pathway components, as shown for human Kibra (KIB), promotes formation of large spherical cytoplasmic puncta (Fig 5B), with these puncta incorporating active Hpo kinase as indicated by pLATS immunostaining (Fig 5B) and co‐localization with GFP‐LATS1 (Fig EV5A). The number and size of cytoplasmic puncta induced by KIB expression increases linearly with the concentration of KIB in the cell (Fig EV5B). Crucially, the number and size of KIB puncta, and their ability to activate pLATS, depend upon the presence of two intrinsically disordered regions (IDRs) within KIB (Fig 6A–D). Thus, the ability of KIB to activate signalling depends upon its IDRs and its ability to promote phase separation in the cytoplasm.

Despite the key nucleating role of upstream pathway components, overexpression of the Hpo kinase itself is sufficient to induce both signalling and formation of punctae in *Drosophila*, which can be observed in the cytoplasm far from the apical membrane where the upstream pathway components normally concentrate (Fig 1A–D). Thus, our results predict that the core Hpo‐Sav complex alone may have the capacity to phase separate without upstream pathway components. To test the notion that Hpo‐Sav complexes themselves are able to induce phase separation at sufficiently high concentrations, we sought to analyse purified complexes *in vitro*. We first expressed and purified a soluble complex of human mYFP‐SAV1:MST2. We find that the complex undergoes phase separation to form distinct puncta in a concentration‐dependent manner and these puncta can be disrupted by 1,6‐hexanediol (Fig EV5C). Additionally, fewer condensates are observed for complexes containing a ∆N variant of SAV1 that lacks the predicted disordered regions (Fig EV5B and C). In cell culture, expression of monomeric YFP‐tagged SAV1 together with mCherry‐tagged MST2 was sufficient to form punctae in which both proteins co‐localise (Fig EV5D and E). These results demonstrate that MST2‐SAV1 complexes can form biomolecular condensates either *in vitro* or upon overexpression in cultured cells.

**Figure 7:**
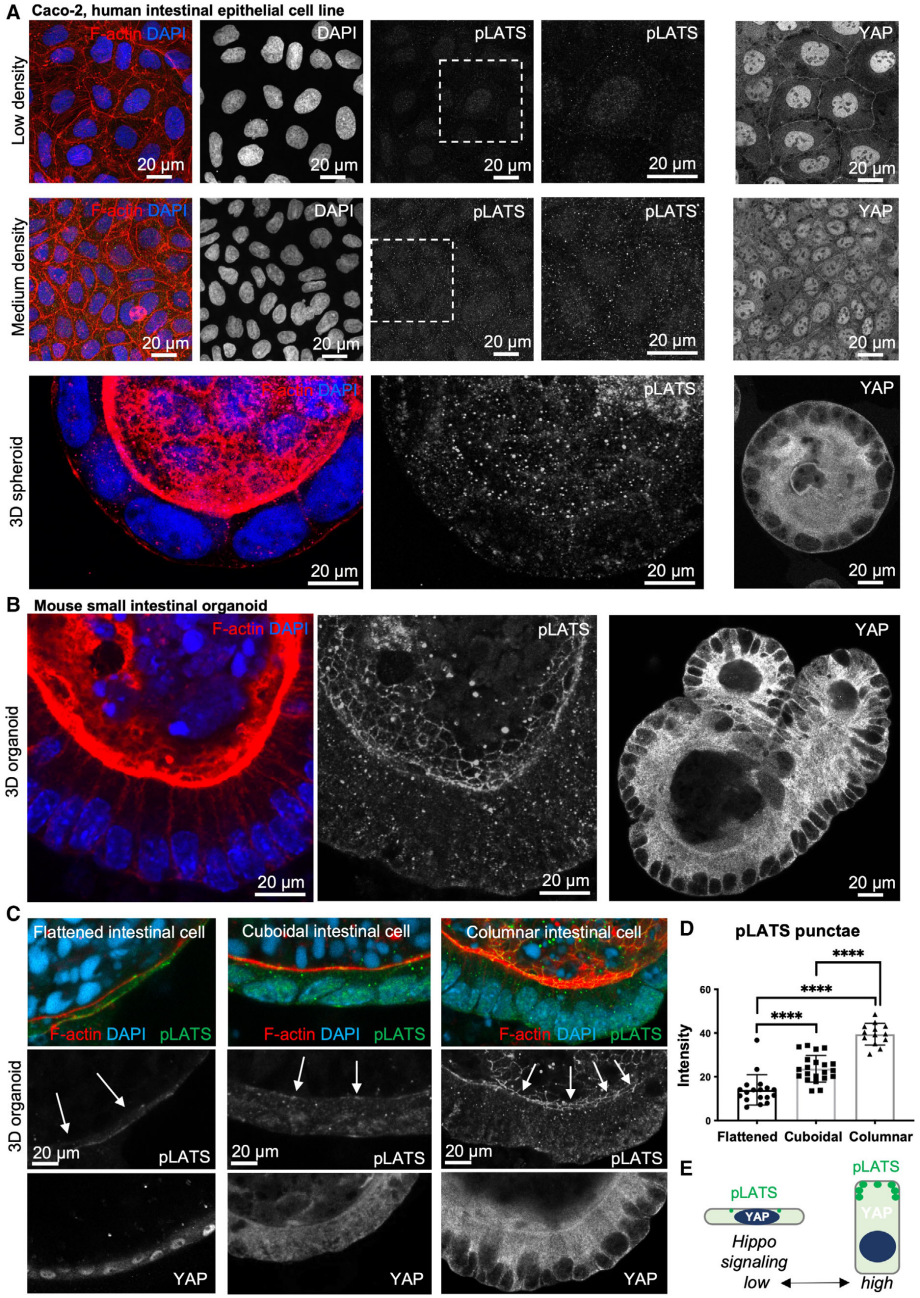
Mechanical strain inhibits the formation of pLATS‐containing punctae and drives YAP nuclear localisation Human Caco2 intestinal epithelial cells are relatively flat when cultured as cell lines in 2D (Caco‐2 cells, top, middle) and cuboidal when cultured in 3D (spheroid, bottom). Punctae of pLATS (green) are strongly enriched at the apical domain of cuboidal cells grown in 3D culture but not in flattened cells grown in 2D culture. Note the increased apical enrichment in medium‐density culture in 2D, where cells become more cuboidal than at low density. Phalloidin staining marks the actin cytoskeleton in red. DAPI marks nuclei in blue.Mouse small intestinal organoids exhibit highly columnar cells which feature a striking concentration of pLATS‐containing punctae into an apical ring and high levels of signalling (YAP becomes largely cytoplasmic).Mouse small intestinal organoids can become stretched upon growth as inflated spheres, which leads to mechanical strain in epithelial cells and reduced pLATS localisation apically, which correlates with reduced Hippo signalling (leading to increased YAP nuclear localisation). White arrows highlight the apical domain of epithelial cells.Quantification of pLATS punctae total intensity in mouse small intestinal organoids from (C). Biological replicates are plotted as individual data points (*n* > 17 for all samples), error bars represent one standard deviation from the mean, statistical significance was determined using a *t*‐test ****P < 0.0001 ***P < 0.001 **P < 0.01 *P < 0.05.Schematic diagram of YAP nuclear localisation in mechanically stretched cells and YAP cytoplasmic localisation in columnar epithelial cells. Source data are available online for this figure.

**Figure EV6:**
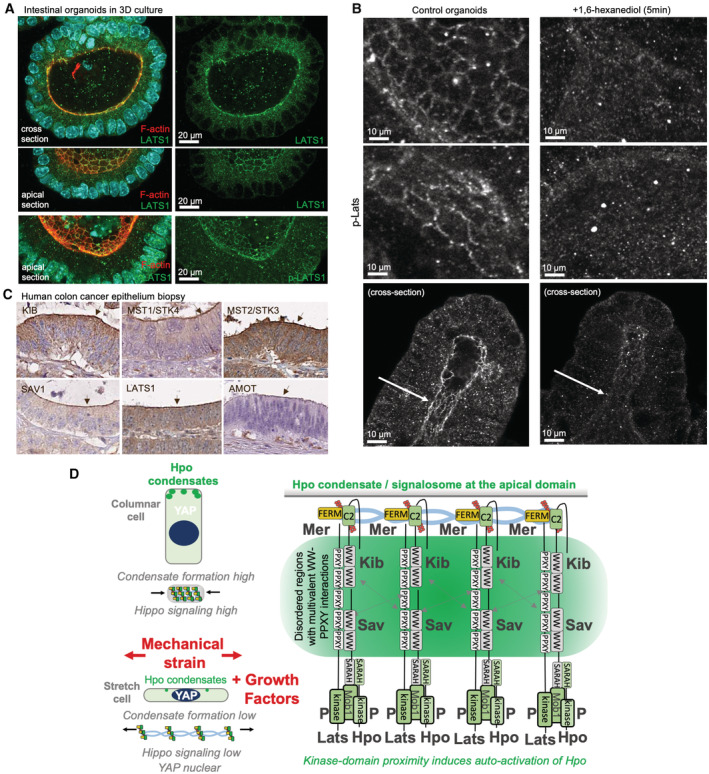
Apical localisation of pLATS and other Hippo pathway components in intestinal organoids and colorectal epithelial cells *in vivo* LATS1 and p‐LATS localise apically in intestinal organoids.Loss of apical p‐LATS enrichment (white arrow) in mouse intestinal organoids treated with 10% 1,6‐hexanediol for 5 min.Hippo pathway components localise apically in colorectal cancer epithelial cells from patient biopsies. Data were mined from the Human Protein Atlas (proteinatlas.org).Schematic diagram of Hippo condensate/signalosome formation at the apical domain and its regulation by mechanical strain and growth factor signals. Source data are available online for this figure.

We next sought to examine whether endogenous Hpo signalling complexes form punctae in polarised human epithelial cells. Endogenous pLATS‐containing punctae localise to the apical surface in 2D cultured Caco2 epithelial cells, particularly with increasing cell density, and even more strikingly upon 3D culture of Caco2 intestinal epithelial cells, which strongly activates signalling and therefore causes YAP retention in the cytoplasm (Fig 7A). In 3D cultured intestinal organoids, which have a highly columnar morphology, pLATS‐containing punctae localise strongly to an apical ring (Fig 7B) and are also sensitive to treatment for 5 min with 1,6‐hexanediol (Fig EV6A and B). Similarly, in biopsies of human colon cancer, endogenous Hpo pathway components concentrate at the apical surface as detected by immunostaining of histological sections (Fig EV6C). Together, these results indicate that endogenous mammalian Hpo signalling complexes can cluster and form condensates that contain active, phosphorylated, LATS kinase at the apical domain of polarised epithelial cells. Thus, mammalian Hpo signalling complexes can undergo phase separation in a similar manner to those in *Drosophila* (Fig EV6D).

Next, we asked whether physiological regulation of Hpo pathway punctae formation by mechanical strain is conserved between *Drosophila* and mammalian cells. We find that stretching of the apical domain (mechanical strain that increases apical area) is sufficient to reduce endogenous pLATS punctae formation in mammalian intestinal epithelial organoids. pLATS‐containing signalling punctae are readily visible at the apical domain of highly columnar cells in mammalian organoids, whereas Hpo signalling punctae are smaller and dimmer when cells are stretched into a flattened morphology, when Hpo signalling is reduced and YAP becomes nuclear localised (Fig 7C and D). This observation indicates that mechanical regulation of Hpo kinase complex formation and signalling at the apical domain is a fundamentally conserved regulatory principle of Hpo signalling.

**Figure 8:**
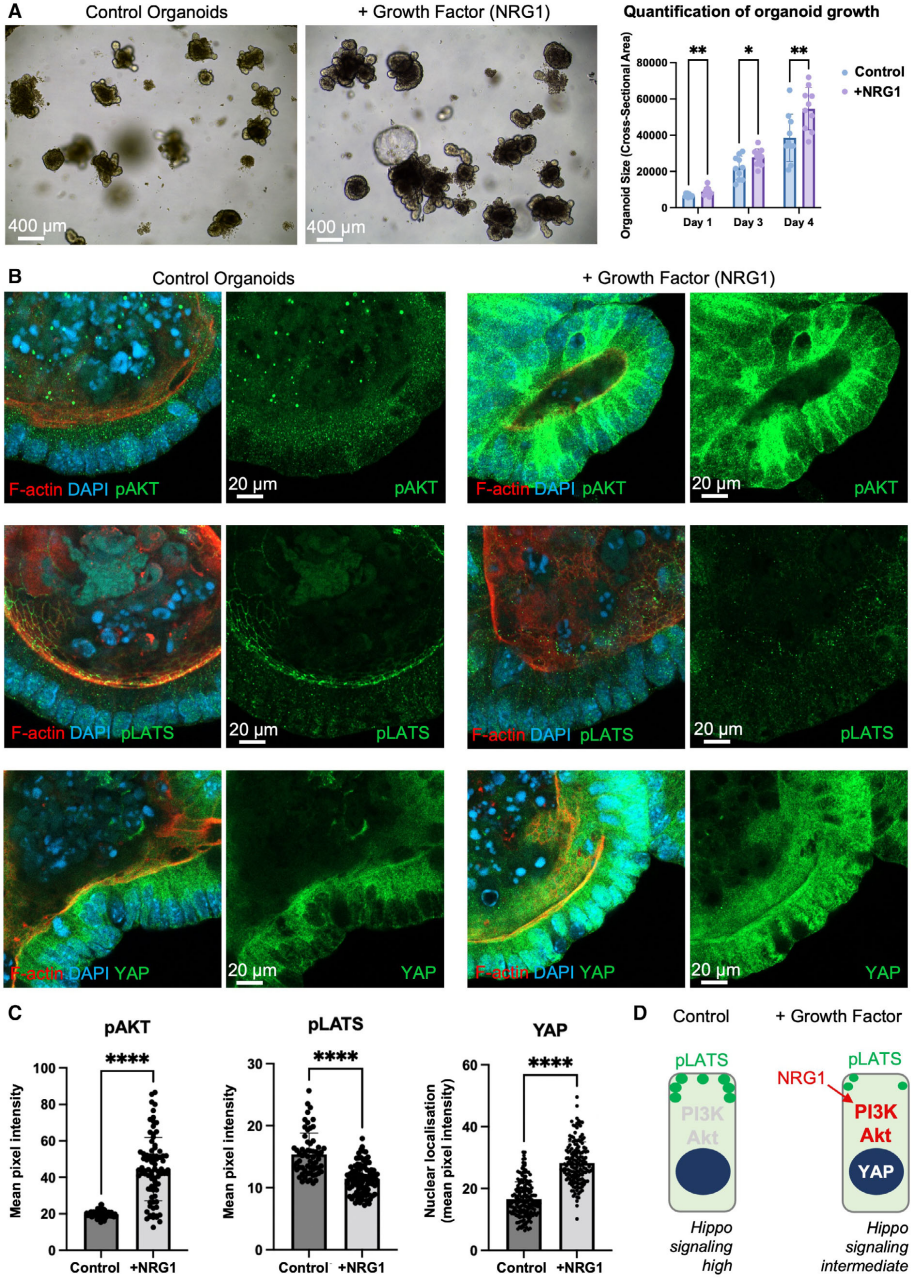
Growth Factor signalling via Akt inhibits pLATS punctae and increases nuclear YAP Murine small intestinal organoids cultured in control medium or medium supplemented with the NRG1 growth factor, which stimulates organoid growth (see graph, right). Biological replicates are plotted as individual data points (*n* > 9 per sample), error bars represent one standard deviation from the mean, statistical significance ****P < 0.0001 ***P < 0.001 **P < 0.01 *P < 0.05.High‐resolution imaging of intestinal organoids cultured in control medium or medium supplemented with the NRG1 growth factor for 1 h which strongly stimulates pAkt, reduces pLATS staining, and drives YAP to the nucleus.Quantification of pAKT, pLATS and YAP immunostaining from (B).Schematic diagram of columnar intestinal epithelial cells with and without growth factor stimulation. Biological replicates are plotted as individual data points (*n* > 40 per sample), error bars represent one standard deviation from the mean, statistical significance was determined using a *t*‐test ****P < 0.0001 ***P < 0.001 **P < 0.01 *P < 0.05. Source data are available online for this figure.

Finally, we sought to test whether growth factor signalling via PI3K‐Akt is sufficient to limit endogenous pLATS punctae formation in mammalian intestinal epithelial organoids, similar to its role in *Drosophila*. We find that the intensity of pLATS punctae at the apical domain is reduced upon activation of Akt by treatment of intestinal organoids with the intestinal growth factor neuregulin (NRG1), which thereby increases YAP nuclear localisation (Fig 8A–D). We selected the growth factor NRG1 because previous evidence indicates that it is a more potent growth factor and activator of pAkt than other growth factors in intestinal organoids (Jarde *et al*, 2020). Our results confirm that growth factor signalling via PI3K‐Akt inhibits Hpo signalling punctae formation and signalling in both *Drosophila* and mammals and is a second fundamentally conserved regulatory principle of Hpo signalling.

## Discussion

Our results indicate that Hpo kinase complexes undergo clustering and phase separation to form biomolecular condensates and suggest condensation as an important mechanism of Hpo signalling. Phase separation into biomolecular condensates is emerging as an important organising principle for compartmentalisation of the cytoplasm and nucleus (Hyman *et al*, 2014; Zhu & Brangwynne, 2015; Banani *et al*, 2017; Shin & Brangwynne, 2017); and recent evidence supports a further role for phase separation in clustering plasma membrane proteins (Li *et al*, 2012; Banjade & Rosen, 2014; Su *et al*, 2016; Tracy *et al*, 2016), including vertebrate tight junctions (Beutel *et al*, 2019; Schwayer *et al*, 2019). In contrast, the possible functional role of biomolecular condensates in signal transduction from the plasma membrane is only beginning to be appreciated (Su *et al*, 2016; Case *et al*, 2019), with Wnt signalling one clearly demonstrated example (Gammons & Bienz, 2018; Case *et al*, 2019; Schaefer & Peifer, 2019). In the case of the Hippo pathway, the downstream nuclear effector YAP was recently shown to undergo phase separation, which may influence its transcriptional activity in the nucleus (Cai *et al*, 2019). However, a role for phase separation in activation of the core Hpo kinase cassette was not previously explored.

Consistent with our observation that Hpo kinase complexes can form condensates *in vitro* and *in vivo*, several components of these complexes (Ex, Kib, Hpo, Sav and Wts) contain intrinsically disordered regions, and undergo multivalent interactions (including via a network of PPXY motif ‐ WW domain binding), both of which are fundamental drivers of polymeric clustering and phase separation in other systems (Li *et al*, 2012; Banjade & Rosen, 2014; Banani *et al*, 2017; Kato & McKnight, 2017; Case *et al*, 2019; Schaefer & Peifer, 2019; Bienz, 2020). While overexpression of Ex or Kib can induce the formation of large spherical Hpo kinase punctae in the cytoplasm, the endogenous Ex and Kib proteins localise to the apical plasma membrane, as Crb‐Ex complexes (at apical cell–cell junctions) or Mer‐Kib complexes (at the medial‐apical region as well as at apical cell–cell junctions) to cluster Hpo kinase complexes. These findings are consistent with recent observations that phase separation can occur via clustering of proteins at the inner surface of the plasma membrane (Case *et al*, 2019), including vertebrate tight junctions (Beutel *et al*, 2019; Schwayer *et al*, 2019), where many upstream Hippo components localise (Karaman & Halder, 2018). Notably, phase separation at vertebrate tight junctions is driven by the ZO‐1 protein, which in *Drosophila* localises to adherens junctions, so cannot explain the behaviour of *Drosophila* Hpo kinase complexes at the apical plasma membrane. Thus, Hpo kinase complexes have an autonomous capacity to cluster, independently of ZO‐1, with either Crb‐Ex or Mer‐Kib at the apical membrane.

Genetic analysis of the Crb‐Ex and Mer‐Kib complexes indicates that they function semi‐redundantly to activate Hpo signalling in *Drosophila* (McCartney *et al*, 2000; Hamaratoglu *et al*, 2006; Baumgartner *et al*, 2010; Genevet *et al*, 2010; Yu *et al*, 2010). Genetic analysis also shows that the spectrin cytoskeleton promotes signalling by both Crb‐Ex and Mer‐Kib complexes in all tissues (Fletcher *et al*, 2015) as well as apical membrane trafficking and formation of microvilli (Khanal *et al*, 2016). Our results show that the spectrin cytoskeleton is also essential to promote the formation of Kib clusters at the membrane and the formation of Hpo kinase punctae apically. Thus, in the follicular epithelium, the spectrin cytoskeleton acts apically with Crb‐Ex and Mer‐Kib to regulate Hpo signalling, where it forms a densely packed network at both apical cell–cell junctions and at the microvillar terminal web. In epithelial tissues that lack conspicuous microvilli, such as the developing *Drosophila* wing epithelium, the apical spectrin cytoskeleton localises primarily to apical cell–cell junctions, where it overlaps with both Crb‐Ex and Mer‐Kib complexes – which largely co‐localise in an apical ring in this tissue (Fletcher *et al*, 2015). Despite these minor differences between the wing epithelium and follicular epithelium, both tissues respond similarly to mechanical strain (stretching epithelial cells into a flattened shape that expands the apical surface area), with Crb‐Ex and Mer‐Kib complexes becoming diluted and Hpo kinase complexes becoming de‐clustered to reduce Hpo signalling and thereby activate Yki‐driven transcriptional responses (Fletcher *et al*, 2018). Thus, the upstream pathway components promote clustering of Hpo kinase punctae at the apical domain and, by virtue of the spectrin cytoskeleton being a mechanically deformable network, these punctae respond to mechanical strain.

Mechanical strain is therefore well established as a key physiological signal regulating Hpo signalling and, importantly, our data suggest that formation of endogenous Hpo biomolecular condensates is regulated by mechanical strain. We have demonstrated that endogenous Hpo‐YFP punctae become de‐clustered in response to flattening of developmentally stretched epithelial cells in *Drosophila*. Our data also show that the ability of mechanical strain to regulate Hpo kinase punctae formation and signalling is conserved between *Drosophila* and mammalian epithelia. We found that endogenous pLATS‐containing punctae form an apical ring in columnar epithelial cells of mouse intestinal organoids and are de‐clustered upon flattening of these cells, which also causes re‐localisation of YAP to the nucleus. These observations suggest that regulation of clustering and phase separation of Hpo complexes by mechanical strain is conserved across animal species.

In addition to sensing mechanical strain, Hippo signalling can also respond to nutritional input, which induces hormonal Insulin/IGF‐1 Receptor (InR) signalling via the PI3K‐Akt pathway (Strassburger *et al*, 2012; Fan *et al*, 2013; Borreguero‐Munoz *et al*, 2019). Starvation reduces PI3K‐Akt activity and thereby leads to increased Hippo signalling to restrict cell proliferation (Borreguero‐Munoz *et al*, 2019). Our results show that inhibition of PI3K‐Akt promotes the formation of large Hpo kinase punctae, mimicking the effect of starvation. Ectopic activation of Akt is sufficient to prevent the formation of large Hpo kinase punctae under conditions of starvation. Thus, the physiological effects of nutrient restriction on Hpo kinase condensates are mediated by Akt. This role of Akt appears to be direct, rather than mediated via the Rheb‐TORC1 pathway, as ectopic activation of Rheb‐TORC1 signalling is not sufficient to prevent the formation of large Hpo kinase condensates under conditions of starvation. These findings are consistent with our previous observation that Hippo signalling is unaffected in *rheb* mutant clones in the *Drosophila* ovarian follicle cell epithelium (Borreguero‐Munoz *et al*, 2019). Importantly, our data confirm that growth factor regulation of Hippo signalling is a conserved principle between *Drosophila* and mammalian epithelial cells. We have shown that activation of Akt upon growth factor stimulation of mouse intestinal organoids is sufficient to reduce formation of pLATS‐containing apical punctae and consequently promote re‐localisation of YAP to the nucleus. Thus, the results suggest that regulation of Hpo clustering and phase separation by growth factor‐induced PI3K‐Akt signalling are conserved across animal species.

The human Hpo kinase complex can be observed to undergo phase separation *in vitro*, with purified MST2‐SAV1 condensates, as well as observed to form punctae in the cytoplasm. In human epithelial cells, Hpo signalling punctae containing active pLATS form at the apical domain. Ectopic expression of KIB generates large micron‐scale punctae in cells that activate pLATS and also induce signalling to retain YAP in the cytoplasm. The ability of KIB to form these large spherical punctae depends upon its intrinsically disordered regions (IDRs), in accordance with the notion that punctae formation is mediated by phase separation. In human epithelial cells, endogenous pLATS‐containing punctae form strongly at the apical domain of densely packed cells in culture, while forming less frequently and at smaller sizes in flattened epithelial cells. In human cells that completely lack an apical domain (such as U2OS cells), Hippo pathway components form much smaller and dispersed clusters in the cytoplasm. Thus, as in *Drosophila*, the formation of human Hpo signalling punctae correlates with the clustering of upstream pathway components and is able to control Hpo signalling activity to YAP in human cells. Collectively, our results support a “Hpo signalosome” model in which clustering and phase separation promotes kinase domain proximity to drive auto‐phosphorylation and signal transduction (Fig EV6D).

To explore the implications of our findings beyond epithelial tissues, we considered one cell type that shares common features with epithelial cells: neurons. In particular, “postsynaptic densities” are membrane domains at which phase separation of localised membrane‐associated proteins such as PSD‐95 can occur to generate discrete clusters along a dendrite that influence the stability of dendritic spines (Cane *et al*, 2014; Zeng *et al*, 2016). At least one upstream Hippo pathway component, Kibra, localises to post‐synaptic densities via WW‐PPXY motif interactions with the post‐synaptic density enriched protein Dendrin (Heitz *et al*, 2016; Tracy *et al*, 2016; Ji *et al*, 2019). Inhibiting the Kibra‐Dendrin interaction blocked long‐term potentiation and impaired spatial learning and memory in mice (Ji *et al*, 2019), while *kibra* knockout mice altered the morphology of dendritic spines (Blanque *et al*, 2015). Thus, the ability of upstream Hippo pathway components to induce phase separation and condensation may be a universal feature of their molecular mechanism in both neurons and epithelial cells. Finally, while this paper was under review, Wang *et al* (2022) reported similar findings.

## Materials and Methods

### *Drosophila* genetics

Expression of UAS‐driven transgenes in ovarian follicle cells was performed with the *TJ*.*Gal4* driver. Fly crosses were kept at 25°C. Ovaries were dissected and immunostained from adult females 3–4 days after eclosion. For starvation experiments, females of the desired genotype were fed on wetted yeast for 2 days (with males for company) before being transferred to vials containing cotton balls soaked in a 20% sucrose solution for 24 h.

For the induction of mutant clones, adult females carrying the heat shock inducible FLP recombinase enzyme (hs.flp) and homozygous FLP recombinase target (FRT) sites were heat shocked in a 37°C water bath for 1 h to induce sister chromatid recombination during mitosis, allowing for the generation of homozygous mutant daughter cell lacking GFP and a homozygous GFP^+^ “twin spot” daughter cell. The mutant clone and twin were allowed to proliferate for 2–3 days prior to dissection of adult females and fixation of ovaries.

### *Drosophila* ovary treatment with 1,6‐hexanediol or sorbitol

For treatment with 1,6‐hexanediol, ovaries were first dissected into PBS and subsequently transferred into 10%(w/v) 1,6‐hexanediol (Sigma) prepared in PBS (or PBS alone for control) for 5 min prior to fixing. For *in vivo* molecular crowding experiments, ovaries were dissected into room temperature (RT) Schneider's media, then incubated in 1 M D‐Sorbitol (Sigma) prepared in Schneider's media (or Schneider's alone for control) for 1 h before fixing.

### *Drosophila* immunofluorescence

Ovaries were dissected in PBS, fixed for 20 min in 4% paraformaldehyde (PFA) in PBS, washed for 45 min in PBS/0.1% Triton X‐100 (PBST) and blocked for 30 min in 5% normal goat serum (NGS)/PBST. Primary antibodies were diluted in NGS/PBST, and samples were incubated overnight at 4°C.

Primary antibodies used were FITC‐conjugated anti‐GFP (1:400; Abcam), rat anti‐Crb (1:300, a gift from U.Tepass), anti‐Ex (gift from R. Fehon), rabbit anti‐Kib (1:200, Genevet *et al*, 2010), rabbit anti‐aPKCζ (C20) (1:250 Santa Cruz), anti‐pWts (gift from K. Irvine), anti‐Rab5 (DSHB), anti‐Rab7 (DSHB), anti‐Rab11 (DSHB), anti‐alpha‐spec (DSHB). Secondary antibodies (goat Alexa Fluor 488, 546, 647; Invitrogen) were used at 1:500 for 2 h at RT, and samples were mounted in Vectashield (Vector Laboratories).

### Image acquisition of fixed *Drosophila* egg chambers

Confocal images were taken with a Leica SP5 or Zeiss LSM780 confocal microscope using 40× oil immersion objectives and processed with Adobe Photoshop and Fiji. Optical cross‐sections through the middle of the egg chamber or through the apical domain are shown in all figures.

### Live imaging of *Drosophila* egg chambers

*Drosophila* ovaries from starved females were dissected into Schneider's media and egg chambers manually removed from the muscular sheath. Egg chambers of the correct stage were transferred to a 29 mm imaging dish (Cellvis) in 200 μl fresh Schneider's media (without supplementation to reproduce starvation conditions). Live confocal imaging was performed with a Leica SP5, with frames collected every 10 s.

### FRAP in *Drosophila* follicular epithelium

FRAP experiments were performed on HpoKD‐Venus puncta formed under starvation conditions, using the Leica SP5 FRAP wizard application. Individual puncta were selected by a circular region (~2 μm) and subject to three bleaching iterations using the 488‐argon laser set to 100% and the Zoom In function of the FRAP wizard. The 514 laser at 20% laser power was used to acquire six pre‐bleach frames (1 s/frame) and 150 post‐bleach frames (50 at 1 s/frame, 50 at 3 s/frame, 50 at 10 s/frame). Raw fluorescent intensity values were measured using the Leica Application Suite X (LAS X) software, and data were subject to the following normalisation: (i) background intensity was subtracted from bleached ROI. Background was determined by placing an identically sized ROI in the nucleus. (ii) The background corrected bleached ROI was normalised to the pre‐bleach frames. (iii) Two background corrected, non‐bleached ROI's were selected to correct for acquisition bleaching.

### *Drosophila* genotypes

All *Drosophila* strains are described in Flybase (www.flybase.org) and are available from the Bloomington *Drosophila* Stock Centre (BDSC) or upon request. The *Drosophila* genotypes used are listed in Table 1.

**Table 1:** Summary of *Drosophila* genotypes, corresponding to figure number.

Fig 1	A, B	TJ.Gal4/HpoKD VC; +/HpoKD VN
	D	+/TJ.Gal4, HpoKD VC; +/HpoKD VN UAS.p60/TJ.Gal4, HpoKD VC; +/HpoKD VN +/TJ.Gal4, HpoKD VC; UAS.myrAkt/HpoKD VN
Fig 2	A–E	TJ.Gal4/HpoKD VC; +/HpoKD VN
	G, H	TJ.Gal4/HpoKD VC; +/HpoKD VN
Fig 3	A, C	w; Hpo‐YFP; +
Fig 4	A	w; Ex‐YFP (R.Fehon)
	B	+/TJ.Gal4, HpoKD VC; +/HpoKD VN
	C, F	TJ.Gal4/UAS.Ex; +
	D, E	UAS.Ex/TJ.Gal4, HpoKD VC; +/HpoKD VN
	G	UAS.Kib/TJ.Gal4, HpoKD VC; +/HpoKD VN
	H	TJ.Gal4/UAS.KibGFP; +
Fig EV1	A	Crb‐GFP
	B	+; Kibra‐GFP
	C	+; GFP‐Wts/TM6b (K.Irvine)
	D, J	w; Hpo‐YFP; + (R.Fehon)
	E, K	TJ.Gal4/HpoKD VC; +/HpoKD VN
	F, G	w; Hpo‐YFP; +
	H, I	TJ.Gal4/HpoKD VC; +/Sav VN8
Fig EV2	B	+/TJ.Gal4, HpoKD VC; +/HpoKD VN +/TJ.Gal4, HpoKD VC; UAS.myrAkt/HpoKD VN +/TJ.Gal4, HpoKD VC; UAS.Rheb/HpoKD VN
	E	+/TJ.Gal4, HpoKD VC; UAS.Atg8‐mCherry/HpoKD VN
Fig EV3	A, B	+/TJ.Gal4, HpoKD VC; +/HpoKD VN UAS.Ex/TJ.Gal4, HpoKD VC; +/HpoKD VN
	C	TJ.Gal4/HpoKD VC; +/HpoKD VN
	D, E	w; Hpo‐YFP; +
Fig EV4	A	+/TJ.Gal4, HpoKD VC; +/HpoKD VN UAS.α‐spec RNAi (VDRC 25387)/TJ.Gal4, HpoKD VC; +/HpoKD VN
	C	βH‐spec‐YFP
	D, E	w1118 hsflp; FRT80B GFP / FRT80B α‐spec
	F	UAS.Kib RNAi (VDRC 106507)/TJ.Gal4, HpoKD VC; +/HpoKD VN

### Mammalian cell culture

Human Caco‐2 adenocarcinoma colon cells were grown in MEM (Gibco), supplemented with MEM non‐essential amino acids (Gibco), 1 mM sodium pyruvate (Gibco), 10% fetal bovine serum (FBS, Sigma) and 100 units/ml penicillin/streptomycin (Gibco). HEK293T cells were grown in DMEM (Gibco), supplemented with 2 mM L‐glutamine (Gibco), 10% FBS and 100 units/ml penicillin/streptomycin. Cells were maintained in a 37°C incubator at 5% atmospheric CO_2_. Cells were subject to mycoplasma testing.

HEK293T and Caco‐2 cells were grown on glass coverslips and transfected using Lipofectamine 3000 Reagent according to manufacturer's instructions. Cells were fixed 24 and 48 h post‐transfection, respectively. The following plasmids were used; Origene: hWWC1(Kibra)‐myc‐DDK (RC228642), Addgene: pEGFP‐C3‐LATS1 (19053), VectorBuilder: EGFP‐hWWC1 FL (aa 1–1119), EGFP‐hWWC1ΔC (aa 1–789), EGFP‐hWWC1ΔM + C (aa 1–429 + 657–789), hSav1‐EGFP (aa 1–383).

The following culture and transfection conditions were used for hSav1‐YFP and mCherry‐MST2 experiments. U‐2 OS cells (gift from Lingfeng Chen) were cultured in DMEM supplemented with 10% FBS (Gibco), 2 mM L‐glutamine (Corning) and 100 units/ml penicillin/streptomycin (Corning) at 37°C and 5% CO_2_. Caco‐2 cells were cultured in EMEM (ATCC‐30‐2003) supplemented with 20% FBS (Gibco) at 37°C and 5% CO_2_. For confocal imaging, cells were cultured on 8‐well LabTek chambered coverglass dishes (Thermo Scientific) and transfected with 100 ng hSav1‐YFP (Addgene) and/or 100 ng mCherry‐MST2 (Addgene) with Lipofectamine 3000 Reagent (Thermo Fisher, U‐2 OS) or TransfeXTM (ATCC, Caco‐2) according to the manufacturer's instructions. Cells were imaged 18 h after transfection on a Zeiss LSM 880 confocal microscope at 37°C with Plan‐Apochromat 63×/1.40 Oil objective.

### Cell culture immunofluorescence

For 2D culture, Caco‐2 cells were grown on glass coverslips for 72 h prior to fixing. Cells were washed twice with PBS (containing CaCl_2_ and MgCl_2_) and fixed in 4% PFA in PBS for 20 min. Cells were permeabilised with 0.2% Triton X‐100 for 10 min. Thoroughly washed cells were blocked with 5% NGS in PBS (blocking buffer) for 1 h at RT. Primary and secondary antibodies were diluted in blocking buffer. Coverslips were incubated with primary antibody overnight at 4°C. Following washes with PBS, Alexa Fluor secondary antibody was applied for 2 h at RT. Coverslips were mounted using Prolong Diamond (Invitrogen).

To grow Caco‐2 cells as 3D spheroids, coverslips were placed in a 24‐well plate and coated with undiluted Matrigel (Corning). Cells were plated in 2% Matrigel in complete media at a density of 1 × 10^4^ cells/well. Media was changed every second day. Spheroids were fixed on day 5 of culture and processed for immunofluorescence as described for organoids.

Primary antibodies used: rabbit anti‐LATS1 (3477, Cell Signaling), rabbit anti‐pLATS1 (9157, Cell Signaling) and mouse anti‐YAP (sc‐101199, SCBT).

### Organoid culture

Crypts were isolated from the small intestine of C57Bl6 mice. Once removed from the mouse, the intestinal segment was cleaned thoroughly in cold PBS, opened longitudinally and the villi mechanically removed. The cleaned tissue was cut into small pieces, rinsed multiple times in ice cold PBS before being resuspended in 25 ml Gentle Cell Dissociation Reagent (Stemcell Technologies) at RT for 15 min with rocking. Tissue segments were settled by gravity, the supernatant discarded and the tissue resuspended in ice cold PBS containing 0.1% BSA, pipetting up and down three times. The tissue segments were allowed to settle and the supernatant was removed and passed through a 70 μm filter. The filtrate was centrifuged and the pellet resuspended in a final volume of RT complete IntestiCult Organoid Growth Medium (Stemcell Technologies) and Matrigel Growth Factor Reduced Basement Membrane Matrix (Corning) added at equal ratios. The crypt suspension was pipetted onto glass coverslips in a 24‐well plate (50 μl per well) and the Matrigel allowed to solidify for 10 min at 37°C. RT complete IntestiCult Medium was then added to each well (750 μl per well). The medium was changed every 2 days, and the organoids were fixed and processed on day 4–5 of culture. For NRG1‐treated organoids, recombinant human NRG1 (100 ng/ml, R&D Systems) was added to complete IntestiCult Medium from day 0 of culture (for analysis of organoid growth) or 1 h before fixing (for acute stimulation).

### Organoid immunofluorescence

Organoids were washed once in PBS and fixed with 4% PFA in PBS for 30 min. Fixative was added directly to the Matrigel dome‐containing organoids, resulting in dissolution of the matrix and subsequent fixation of the organoids to the coverslip. Organoids were permeabilised with 0.5% Triton X‐100/PBS for 20 min and blocked with 5% NGS in PBS/0.2% Triton X‐100/0.05% Tween‐20 (blocking buffer) for 30 min. Primary antibodies were diluted in blocking buffer and incubated with organoids overnight at 4°C. Secondary antibody containing Phalloidin‐555 was incubated for 2 h at RT, followed by a counterstain with DAPI. Coverslips were mounted in Aqua/Poly‐Mount (Polysciences, Inc.), and organoids were imaged with a Zeiss LSM780 confocal microscope, 40× oil immersion objective.

Primary antibodies were identical to those described for cell line immunofluorescence with the addition of rabbit anti‐pAKT (4060, Cell Signaling).

### Expression and purification of MST2:SAV1 complexes

DNA sequences corresponding to human MST2 (residues 1–484) and MBP‐tagged λ‐phosphatase were cloned into separate opening readings frames in pRSF‐Duet (Novagen). Nucleotides corresponding to human SAV1‐FL (residues 1–383) or a truncated variant, SAV1ΔN, (residues 196–383) were cloned into a modified pGEX‐2TK (Sigma‐Aldrich) encoding an N‐terminal His8‐StrepTagII‐mYFP tag.

T7 Express cells (New England Biolabs) were co‐transformed with both plasmids. Cells were grown in Terrific Broth to an OD600 of 2.0, protein expression induced by 0.25 mM IPTG and cells were additionally grown overnight at 16°C. Cells were lysed in 50 mM Tris pH 8.5, 400 mM NaCl and 10%(v/v) Glycerol. MST2:SAV1 complexes were purified by a combination of immobilised metal affinity chromatograph (IMAC; BioRad), streptactin affinity chromatography (Cytiva) and size‐exclusion chromatography (Superose 200; GE Healthcare). Complex was concentrated to approximately 10 μM in 10 mM Tris pH 8.5, 400 mM NaCl, 5% Glycerol and 5 mM βME and used immediately.

### *In vitro* droplet‐formation assay

MST2:SAV1 complexes were diluted in 10 mM Tris, pH 8.5, 400 mM NaCl and 5 mM βME to the concentrations indicated in the presence or absence of 5% (w/v) 1,6‐hexanediol, incubated for 2 h and 7.5 μL loaded into a chamber made from clean glass slide with a 120‐μm double‐sided sticker (Grace Biolabs). Two micrometer Z sections of each well were taken on a Nikon Eclipse Ti2 using a Hamamatsu digital camera C13440 with a 60× air‐immersion lens. ImageJ was used to compile the Z‐stacks and calculate the total area corresponding to the droplets. Data were graphed and analysed in Prism (GraphPad, La Jolla, California).

### Human cancer biopsy data

Immunostaining of colorectal cancer epithelial cells was mined from the Human Protein Atlas database (proteinatlas.org).

## Author contributions

**Teresa T Bonello:** Data curation; formal analysis; investigation; methodology; writing – review and editing. **Danfeng Cai:** Investigation. **Georgina C Fletcher:** Investigation. **Kyler Wiengartner:** Investigation. **Victoria Pengilly:** Investigation. **Kimberly S Lange:** Investigation. **Zhe Liu:** Investigation. **Jennifer Lippincott‐Schwartz:** Supervision; funding acquisition; project administration. **Jennifer M Kavran:** Supervision; funding acquisition; methodology; project administration. **Barry J Thompson:** Conceptualization; supervision; funding acquisition; writing – original draft; project administration; writing – review and editing.

## Disclosure and competing interests statement

The authors declare that they have no conflict of interest.

## Supporting information



Expanded View Figures PDF

Source Data for Expanded View

PDF+

Source Data for Figure 1

Source Data for Figure 2

Source Data for Figure 3

Source Data for Figure 4

Source Data for Figure 5

Source Data for Figure 6

Source Data for Figure 7

Source Data for Figure 8

## Data Availability

This study includes no data deposited in external repositories.
