# Supplementary material for: Phase separation of Hippo signalling complexes
Source: EMBO J. 2023 Feb 20;42(6):e112863. doi: 10.15252/embj.2022112863 (PMC10015380; doi:10.15252/embj.2022112863)
Supplement: Supplementary file 1 — Expanded View Figures PDF [file EMBJ-42-e112863-s007.pdf]

Expanded View Figures

Figure EV1. Phase separation of Hippo signalling complexes at the apical domain is enhanced by starvation or overexpression.

A Crb-GFP localises apically in cuboidal and columnar epithelial cells of the ovarian follicular epithelium.
B Kibra-GFP localises apically in cuboidal and columnar epithelial cells of the ovarian follicular epithelium.
C Wts-GFP localises apically in cuboidal and columnar epithelial cells of the ovarian follicular epithelium.
D Hpo-YFP protein is mostly cytoplasmic, although weak apical signal is detectable in columnar epithelial cells.
E A HpoKD-Venus dimerization sensor can be detected at the apical domain of densely packed columnar follicle cells, similar to Crb, Kib and Wts.
F Hpo-YFP localises to the apical junctions in the follicular epithelium of control (fed) egg chambers and becomes strongly enriched into apical junction puncta under conditions of nutrient restriction.
G HpoKD-Venus is apically enriched in columnar cells but not in stretch cells (high mag view of right-hand panel in E).
H Hpo-Sav Venus bimolecular fluorescence complementation (BiFC) sensor based on split-Venus proteins fused to Hpo and Sav. Puncta formation in the cytoplasm is evident. Elevated signal at the apical domain is evident.
I Hpo-Sav Venus BiFC sensor levels are elevated upon starvation for 24 h, producing an increase in puncta formation.
J Endogenously tagged Hpo-YFP localises to apical punctae, which are more easily detected when the tagged allele is homozygous, rather than heterozygous.
K Ectopically expressed HpoKD-Venus BiFC sensor forms larger puncta when expressed at higher levels. Quantification of puncta number and size is shown below. Biological replicates are plotted as individual data points.

Source data are available online for this figure.

© 2023 The Authors

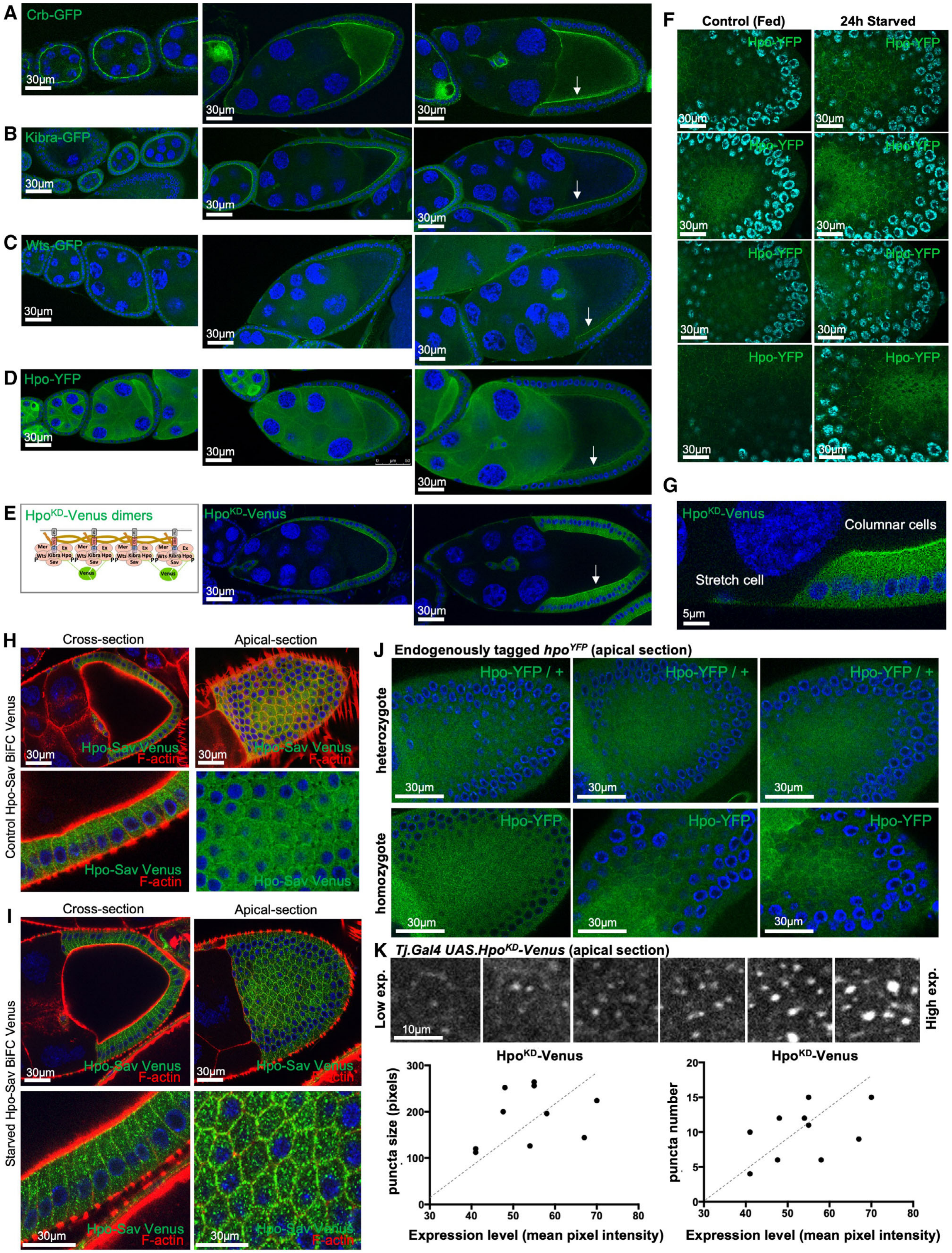

Figure EV1.

 © 2023 The Authors

Figure EV2. Hpo-Venus dimers are phosphorylated at Thr183 and condensate formation is inhibited by ectopic expression of active Akt but not Rheb.

A Clonal expression of HpoKD-Venus dimers stain positively for phospho-Thr183, indicating phosphorylation of the kinase activation loop. Clonal expression of HpoKD-Venus dimers fails to enrich apically in the follicular epithelium when co-expressed with constitutively active Akt. Clonal expression of active Hpo-Venus dimers localises to large puncta within the cytoplasm which stain positive for phospho Thr183.

B Hpo punctae induced by nutrient restriction are diminished by constitutively active Akt signalling in the follicular epithelium, but not by expression of Rheb (which activates TOR).

C Quantification of Hpo punctae size in the clonal conditions described in (A). Biological replicates are plotted as individual data points ($n > 8$ per sample), error bars represent one standard deviation from the mean, statistical significance was determined using a *t*-test ****$P < 0.0001$ ***$P < 0.001$ **$P < 0.01$ *$P < 0.05$.

D Quantification of Hpo punctae size under nutrient restriction in the presence or absence of constitutively active Akt signalling. Biological replicates are plotted as individual data points ($n > 15$ per sample), error bars represent one standard deviation from the mean, statistical significance was determined using a *t*-test ****$P < 0.0001$ ***$P < 0.001$ **$P < 0.01$ *$P < 0.05$.

E Minimal autophagic activity is observed in the follicular epithelium of egg chambers from well-fed females. Nutrient restriction promotes a strong autophagic response in the follicular epithelium. The autophagosome compartment, marked my mCherry-Atg8a, does not colocalise with Hpo puncta.

Source data are available online for this figure.

© 2023 The Authors

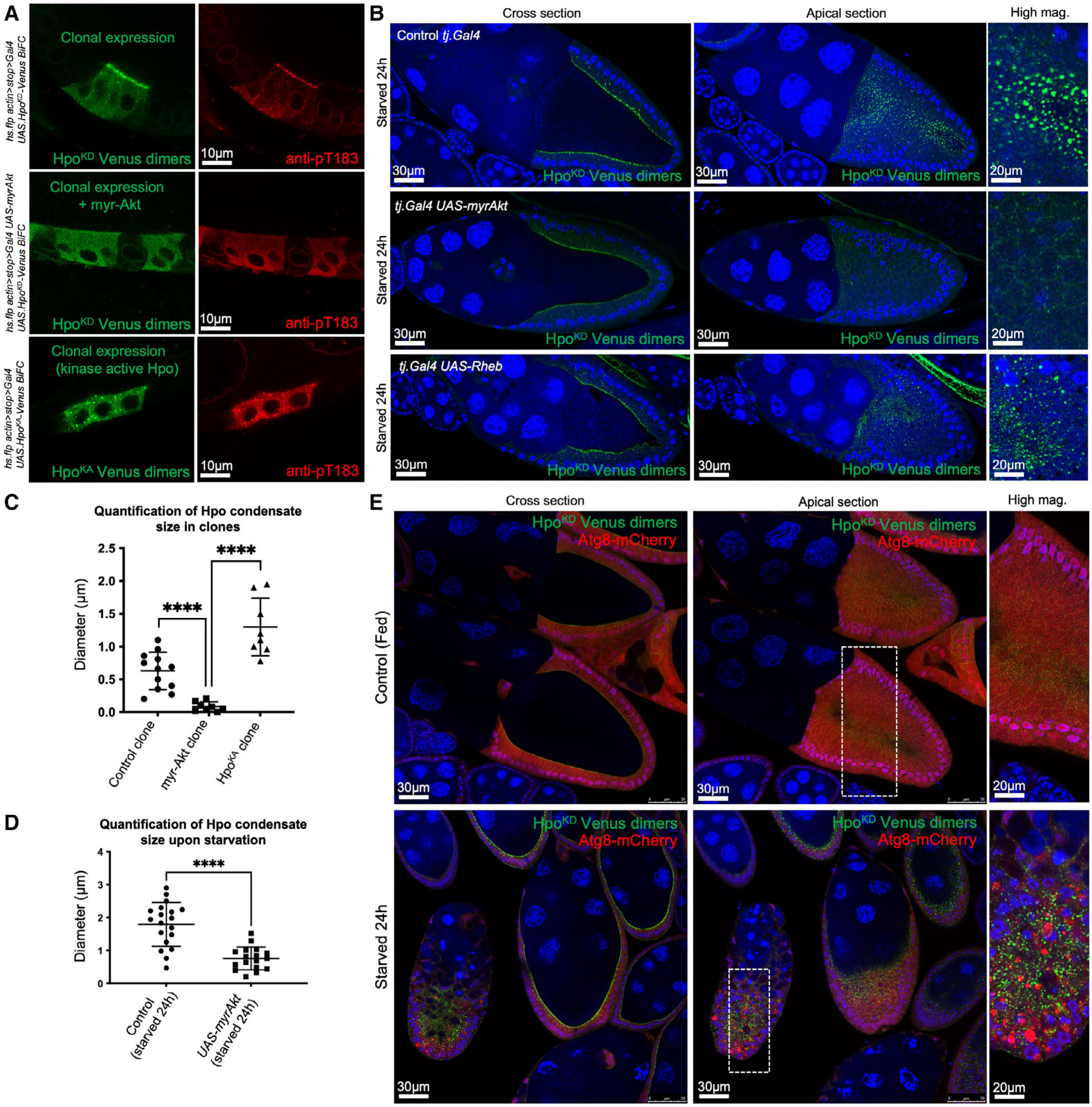

Figure EV2.

Figure EV3. Hpo kinase condensates do not co-localise with endosomal markers and are reversibly sensitive to 1,6-hexanediol treatment.

A Hpo puncta formed at the apical domain of the follicular epithelium under wild-type conditions or strongly recruited into puncta by Ex overexpression, do not co-localise with markers for early (Rab5) or late (Rab7) endosomal compartments.

B Hpo puncta formed at the apical domain of the follicular epithelium under wild-type conditions or Ex overexpression, do not co-localise with Rab11, a marker of recycling endosomes.

C 1,6-hexanediol treatment reversibly disperses HpoKD-Venus apical clusters.

D, E 1,6-hexanediol treatment also disperses endogenous Hpo-YFP clusters that appear under starvation.

Source data are available online for this figure.

 © 2023 The Authors

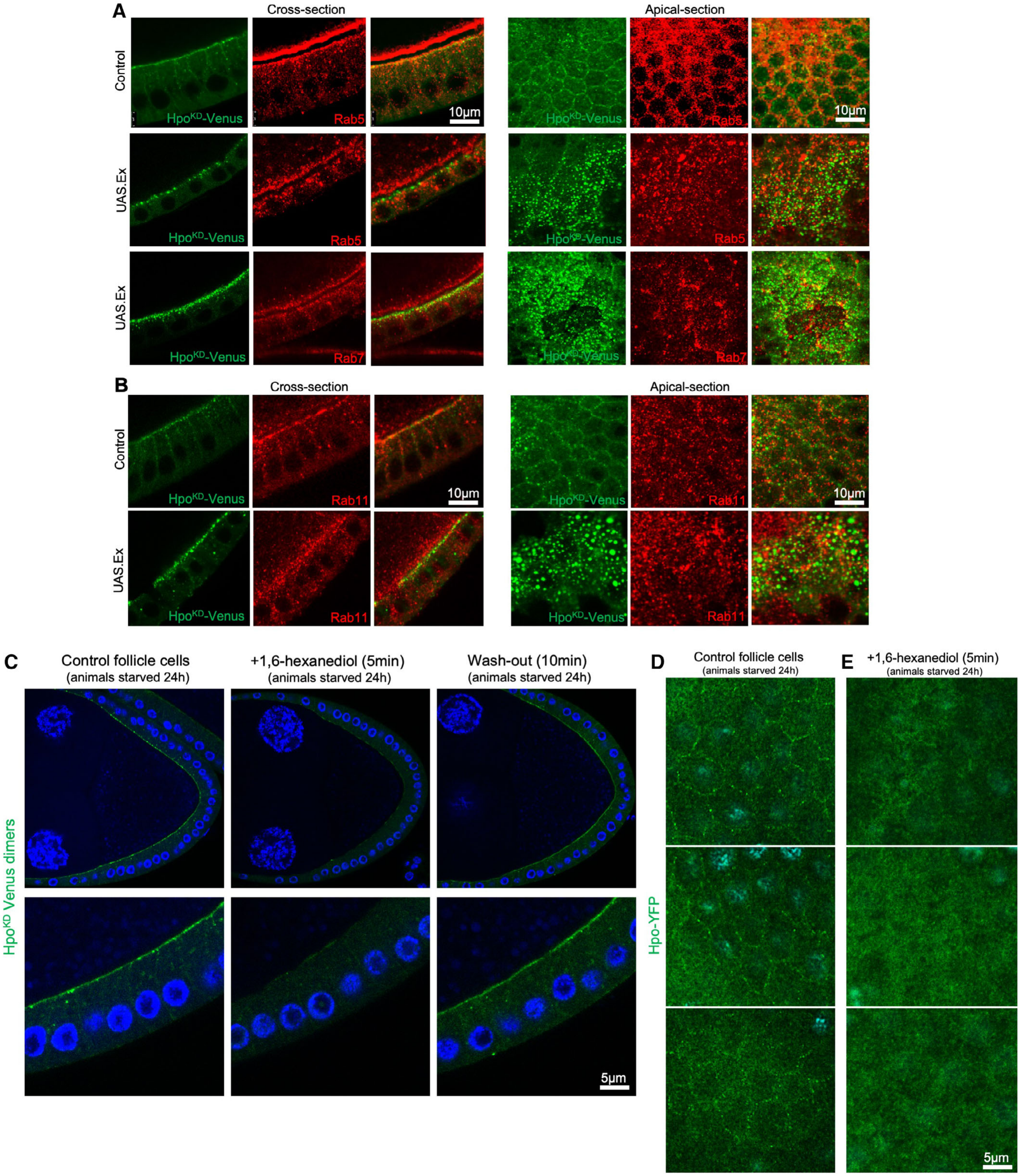
A
Cross-section
Apical-section
Control
UAS.Ex
UAS.Ex
Hpo^{KD}-Venus
Rab5
Rab7
10μm
B
Cross-section
Apical-section
Control
UAS.Ex
Hpo^{KD}-Venus
Rab11
10μm
C
Control follicle cells
(animals starved 24h)
+1,6-hexanediol (5min)
(animals starved 24h)
Wash-out (10min)
(animals starved 24h)
Hpo^{KD} Venus dimers
5μm
D
Control follicle cells
(animals starved 24h)
E
+1,6-hexanediol (5min)
(animals starved 24h)
Hpo-YFP
5μm

Figure EV3.

Figure EV4. The spectrin cytoskeleton promotes assembly of Hippo signalling condensates via local clustering at the apical plasma membrane.

A α-Spectrin is required for active Hpo signalling at the apical domain of follicular epithelial cells.

B Loss of α-Spectrin significantly reduces Hpo puncta size. Quantification of Hpo punctae size in the presence or absence of α-Spectrin-RNAi. Biological replicates are plotted as individual data points ($n > 6$ per sample), error bars represent one standard deviation from the mean, statistical significance was determined using a *t*-test ****$P < 0.0001$ ***$P < 0.001$ **$P < 0.01$ *$P < 0.05$.

C Endogenously tagged β_H-Spectrin forms a dense mesh at the apical domain of follicular epithelial cells in stage 9/10 egg chambers.

D Kibra localises predominantly to the medial-apical region of follicular epithelial cells, and this localisation is dependent on α-Spectrin.

E Mislocalisation of Kibra in α-Spectrin clones is not a consequence of reduced Kibra levels at the apical domain.

F Kibra is required for active Hpo signalling at the apical domain of follicular epithelial cells.

G Disorder prediction of *Drosophila* Hpo pathway proteins, using two independent algorithms. For meta-predictor PONDR-FIT, residues with a score above 0.5 are predicted disordered. The per-residue confidence score (pLDDT) generated by AlphaFold predicts regions below 50 pLDDT to be unstructured in isolation. AlphaFold structure prediction models for Hpo pathway proteins, colour-coded according to model confidence. Orange regions correspond to very low model confidence (pLDDT < 50), predicted to represent intrinsically disordered protein sequence.

H Molecular modelling of *Drosophila* Hpo components using the protein structure prediction server Phyre2. Where protein structures were incomplete or could not be predicted (e.g. Sav, Kib), protein sequences were submitted to I-TASSER. The PDB outputs from both servers were uploaded to Illustrate (https://ccsb.scripps.edu/illustrate/) to generate the graphics. Predictive reconstructions of the Crbs-Ex complexes at the apical junctions and Kib-Mer complexes at the medial-apical domain, highlighting the contribution of intrinsically disordered protein structures to both networks. Ex and Kib nucleate Hpo condensate formation *in vivo*, a process which may be driven by their intrinsically disordered domains.

Source data are available online for this figure.

© 2023 The Authors

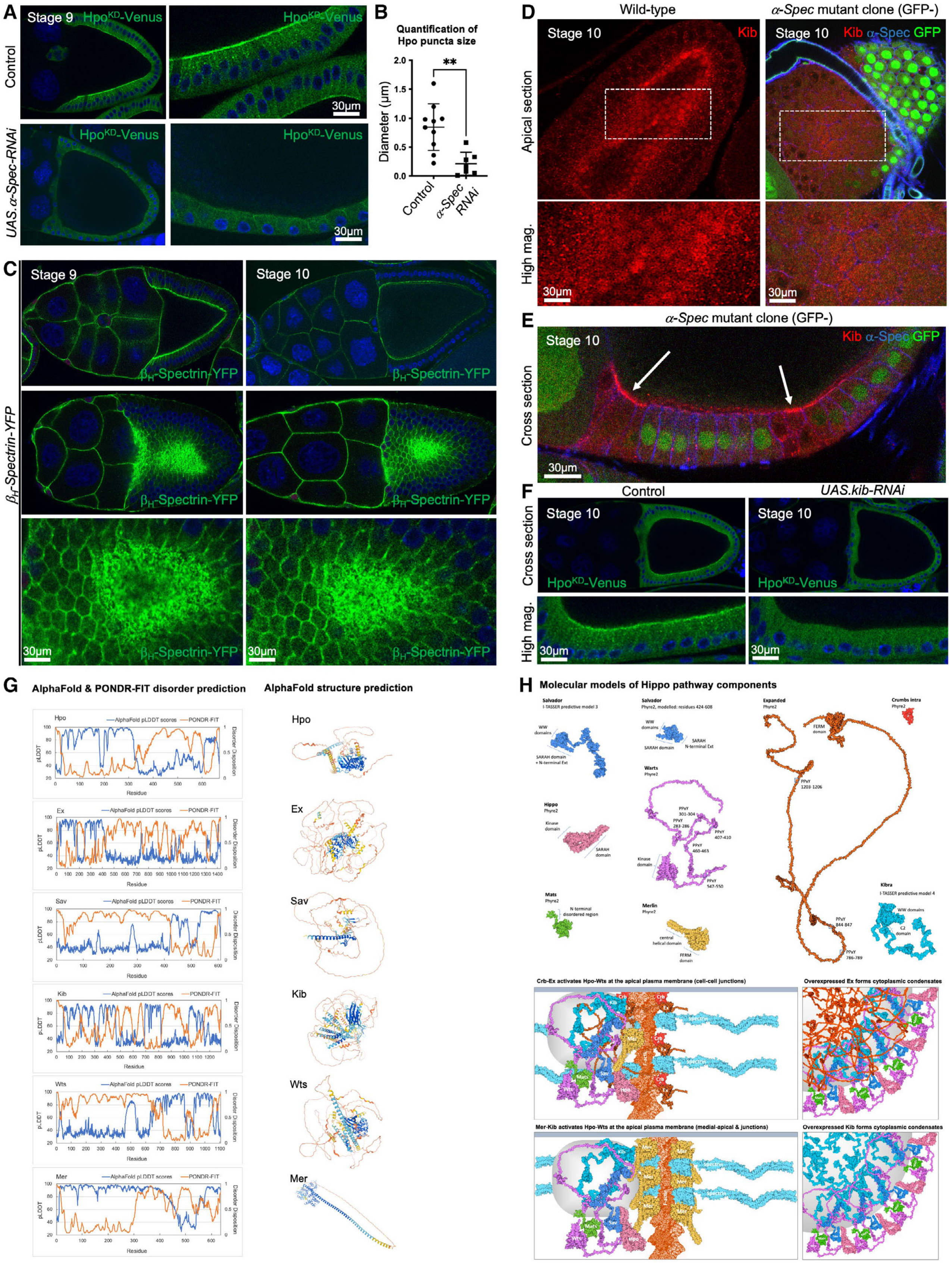
A
Stage 9
Hpo^KD-Venus
Control
UAS.α-Spec-RNAi
30μm
B
Quantification of Hpo puncta size
Diameter (μm)
**
Control
α-Spec RNAi
C
Stage 9
Stage 10
β_H-Spectrin-YFP
D
Wild-type
α-Spec mutant clone (GFP-)
Stage 10
Kib
α-Spec
GFP
Apical section
High mag.
E
Cross section
F
Control
UAS.kib-RNAi
G
AlphaFold & PONDR-FIT disorder prediction
AlphaFold structure prediction
Hpo
Ex
Sav
Kib
Wts
Mer
H
Molecular models of Hippo pathway components
Crb-Ex activates Hpo-Wts at the apical plasma membrane (cell-cell junctions)
Overexpressed Ex forms cytoplasmic condensates
Mer-Kib activates Hpo-Wts at the apical plasma membrane (medial-apical & junctions)
Overexpressed Kib forms cytoplasmic condensates

Figure EV4.

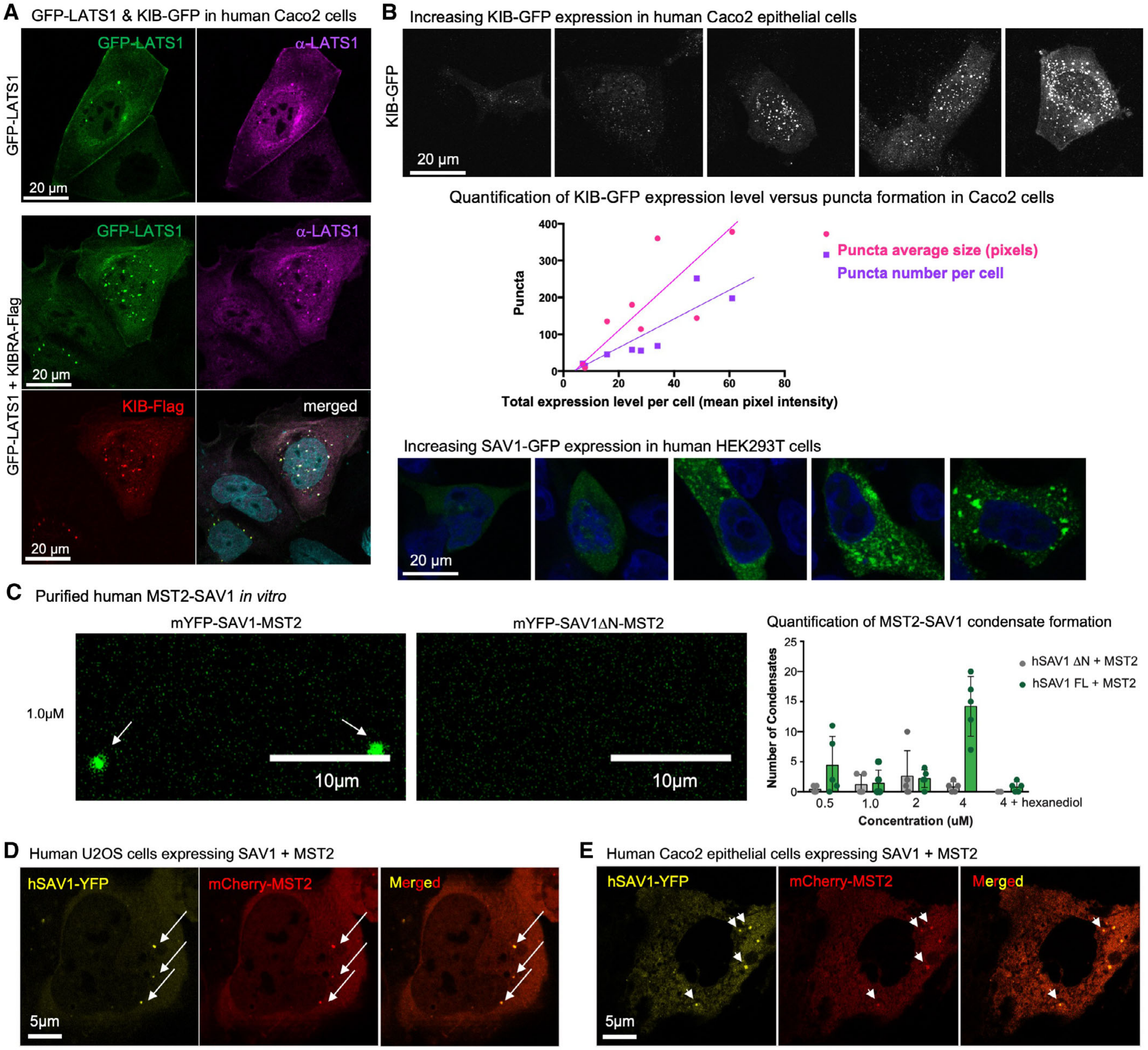

Figure EV5. Phase separation of mammalian Hippo signalling condensates *in vitro* and *in vivo*.

A GFP-LATS1 forms cytoplasmic punctae when expressed in Caco2 epithelial cells. KIB-Flag colocalises with GFP-LATS1 in cytoplasmic punctae when co-expressed in Caco2 epithelial cells.

B Ectopic KIB-GFP expression in human Caco2 epithelial cells causes formation of punctae in the cytoplasm. Quantification of KIB puncta size and number reveals a linear correlation with the total expression level of KIB in the cell. Ectopic SAV1-GFP expression in human Caco2 epithelial cells causes the formation of punctae in the cytoplasm.

C Purified mYFP-SAV1:MST2 complex undergoes phase separation to form distinct puncta in a concentration-dependent manner. Additionally, fewer condensates are observed for complexes containing a variant of SAV1 that lacks the predicted disordered regions, or upon treatment with 1,6-hexanediol. Biological replicates are plotted as individual data points ($n > 4$ per sample), error bars represent one standard deviation from the mean, statistical significance was determined using a *t*-test ****$P < 0.0001$ ***$P < 0.001$ **$P < 0.01$ *$P < 0.05$.

D In U-2 OS cells, expression of monomeric YFP-tagged SAV1 together with mCherry-tagged MST2 was sufficient to form condensates in which both proteins co-localise.

E In Caco-2 epithelial cells, expression of monomeric YFP-tagged Sav1 together with mCherry-tagged MST2 was sufficient to form condensates in which both proteins co-localise.

Source data are available online for this figure.

 © 2023 The Authors

Figure EV6. Apical localisation of pLATS and other Hippo pathway components in intestinal organoids and colorectal epithelial cells *in vivo*.

A LATS1 and p-LATS localise apically in intestinal organoids.

B Loss of apical p-LATS enrichment (white arrow) in mouse intestinal organoids treated with 10% 1,6-hexanediol for 5 min.

C Hippo pathway components localise apically in colorectal cancer epithelial cells from patient biopsies. Data were mined from the Human Protein Atlas (proteinatlas.org).

D Schematic diagram of Hippo condensate/signalosome formation at the apical domain and its regulation by mechanical strain and growth factor signals.

Source data are available online for this figure.

© 2023 The Authors

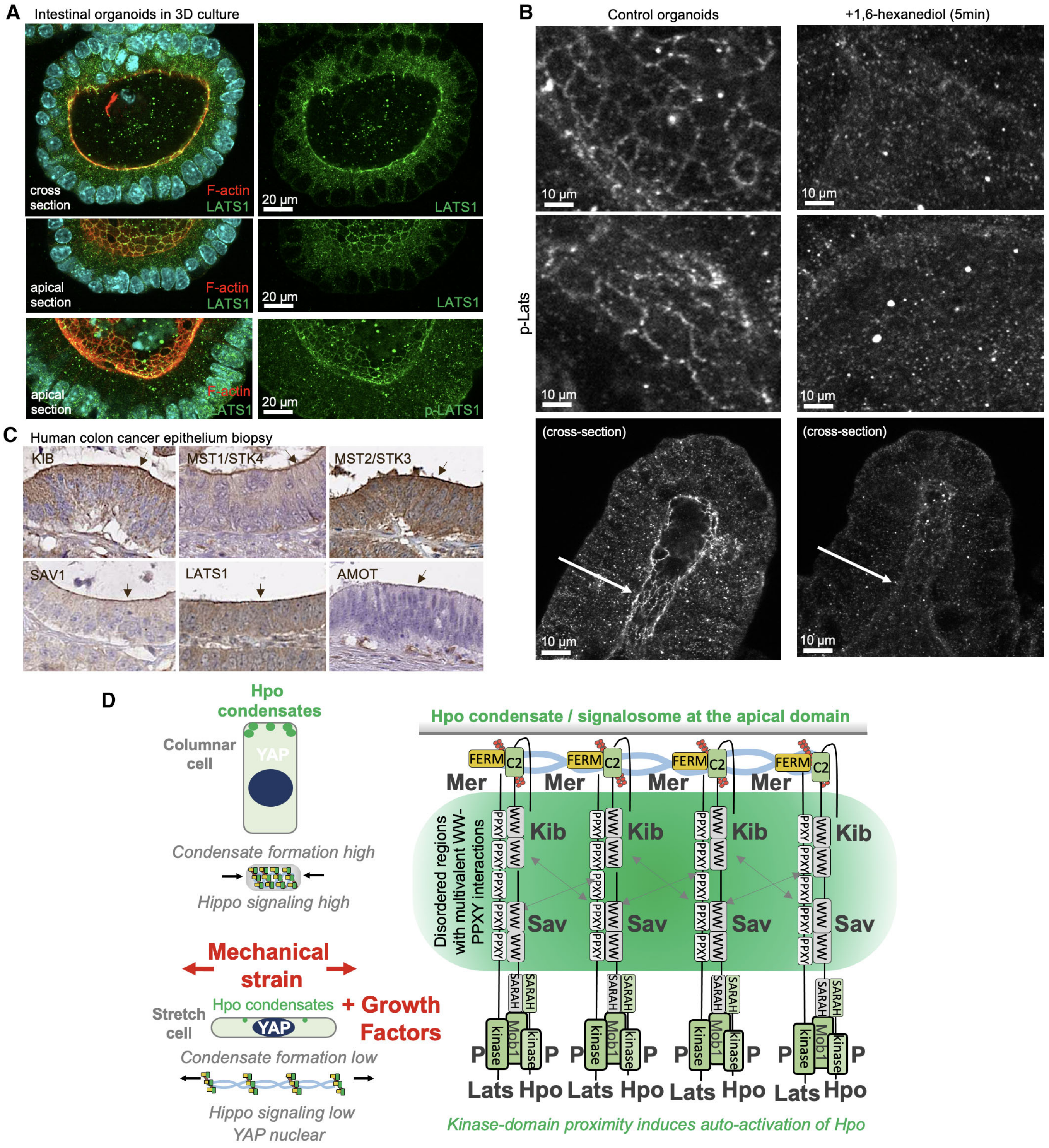

Figure EV6.

 © 2023 The Authors
