# Supplementary material for: Phase separation of Hippo signalling complexes
Source: EMBO J. 2023 Feb 20;42(6):e112863. doi: 10.15252/embj.2022112863 (PMC10015380; doi:10.15252/embj.2022112863)
Supplement: Supplementary file 3 — PDF+ [file EMBJ-42-e112863-s005.pdf]

Article

SOURCE DATA | TRANSPARENT PROCESS | OPEN ACCESS

THE EMBO JOURNAL

Phase separation of Hippo signalling complexes

Teresa T Bonello[1], Danfeng Cai[2,3], Georgina C Fletcher[4], Kyler Wiengartner[3], Victoria Pengilly[1], Kimberly S Lange[3], Zhe Liu[2], Jennifer Lippincott-Schwartz[2], Jennifer M Kavran[3,5] & Barry J Thompson[1,4,*]

Abstract

The Hippo pathway was originally discovered to control tissue growth in *Drosophila* and includes the Hippo kinase (Hpo; MST1/2 in mammals), scaffold protein Salvador (Sav; SAV1 in mammals) and the Warts kinase (Wts; LATS1/2 in mammals). The Hpo kinase is activated by binding to Crumbs-Expanded (Crb-Ex) and/or Merlin-Kibra (Mer-Kib) proteins at the apical domain of epithelial cells. Here we show that activation of Hpo also involves the formation of supramolecular complexes with properties of a biomolecular condensate, including concentration dependence and sensitivity to starvation, macromolecular crowding, or 1,6-hexanediol treatment. Overexpressing Ex or Kib induces formation of micron-scale Hpo condensates in the cytoplasm, rather than at the apical membrane. Several Hippo pathway components contain unstructured low-complexity domains and purified Hpo-Sav complexes undergo phase separation *in vitro*. Formation of Hpo condensates is conserved in human cells. We propose that apical Hpo kinase activation occurs in phase separated "signalosomes" induced by clustering of upstream pathway components.

Keywords condensates; epithelia; Hippo signalling; mechanobiology
Subject Categories Development; Signal Transduction
DOI 10.15252/embj.2022112863 | Received 18 October 2022 | Revised 12 January 2023 | Accepted 23 January 2023
The EMBO Journal (2023) e112863

Introduction

The Hippo signalling pathway was discovered in *Drosophila* to restrict the growth of proliferating epithelial tissues (Boggiano & Fehon, 2012; Irvine & Harvey, 2015; Karaman & Halder, 2018; Misra & Irvine, 2018; Davis & Tapon, 2019; Zheng & Pan, 2019) and has important roles in tissue regeneration and cancer (Moya & Halder, 2019; Dey *et al*, 2020; Thompson, 2020). The core components of the pathway include the upstream kinase Hippo (Hpo; MST1/2 in mammals; Harvey *et al*, 2003; Pantalacci *et al*, 2003; Udan *et al*, 2003; Wu *et al*, 2003), its adaptor protein Salvador (Sav; SAV1 in mammals; Kango-Singh *et al*, 2002; Tapon *et al*, 2002), the downstream kinase Warts (Wts; LATS1/2 in mammals; Justice *et al*, 1995; Xu *et al*, 1995) and its adaptor protein Mats (MOB1 in mammals; Wei *et al*, 2007; Praskova *et al*, 2008).

Activation of the Hpo kinase involves binding to Sav and autophosphorylation in its activation loop (Creasy *et al*, 1996; Praskova *et al*, 2004; Jin *et al*, 2012; Deng *et al*, 2013; Ni *et al*, 2013) and linker region (Ni *et al*, 2015) in order to bind Mats/MOB1 and activate Wts/LATS (Ni *et al*, 2015). Activated Wts then phosphorylates the nuclear transcriptional co-activator Yorkie (Yki; YAP/TAZ in mammals) to retain it in the cytoplasm by inducing interaction with 14-3-3 proteins (Kanai *et al*, 2000; Huang *et al*, 2005; Zhao *et al*, 2007; Oh & Irvine, 2008, 2009). Hpo kinase autophosphorylation can also be promoted by the Tao-1 kinase (Boggiano *et al*, 2011; Poon *et al*, 2011) and can be antagonised by the STRIPAK PP2A phosphatase complex (Ribeiro *et al*, 2010; Zheng *et al*, 2017). Recent work revealed that the fundamental principle governing activation of the Hpo/MST kinase and autophosphorylation is the physical proximity of the kinase domains (Tran *et al*, 2020). Thus, the biochemistry of the core Hpo-Sav-Wts-Mats kinase complex and its ability to regulate Yki/YAP/TAZ are well understood, but the upstream signalling mechanisms that control activation of the Hpo kinase remain unclear.

Hpo kinase activation also depends upon several key upstream pathway components that localise to the apical plasma membrane of epithelial cells, including the Crumbs-Expanded (Crb-Ex) and Merlin-Kibra (Mer-Kib) complexes (McCartney *et al*, 2000; Hamaratoglu *et al*, 2006; Baumgartner *et al*, 2010; Chen *et al*, 2010; Genevet *et al*, 2010; Ling *et al*, 2010; Robinson *et al*, 2010; Yu *et al*, 2010), which interact with the spectrin cytoskeleton (Medina *et al*, 2002; Deng *et al*, 2015; Fletcher *et al*, 2015). Crb-Ex complexes localise primarily in an apical-lateral ring of cell–cell junctions (similar to vertebrate tight junctions), while Mer-Kib complexes localise both to the apical-lateral ring and to the medial-apical region (Sun *et al*, 2015; Su *et al*, 2017). In addition, Sav is localised both to apical-lateral cell–cell junctions (Yue *et al*, 2012) and to the medial-apical region (Sun *et al*, 2015; Su *et al*, 2017). In contrast, Hpo is broadly distributed in the cytoplasm, although with some enrichment apically that overlaps with both apical-lateral cell–cell

1 EMBL Australia, John Curtin School of Medical Research, Australian National University, Canberra, ACT, Australia
2 HHMI Janelia Research Campus, Ashburn, VA, USA
3 Department of Biochemistry and Molecular Biology, Bloomberg School of Public Health, Baltimore, MD, USA
4 Epithelial Biology Laboratory, The Francis Crick Institute, London, UK
5 Department of Biophysics and Biophysical Chemistry, and Department of Oncology, Johns Hopkins School of Medicine, Baltimore, MD, USA
*Corresponding author. Tel: +61 436 838 584; E-mail: barry.thompson@anu.edu.au

© 2023 The Authors. Published under the terms of the CC BY 4.0 license

junctions and the medial-apical region (Sun *et al*, 2015; Su *et al*, 2017). Similarly, the bulk distribution of Wts is largely cytoplasmic, yet its signalling activity is strongly induced by recruitment to the apical plasma membrane (Yin *et al*, 2013).

Importantly, signal transduction can be activated simply by overexpression of the upstream activators Ex or Kib (Hamaratoglu *et al*, 2006; Baumgartner *et al*, 2010; Sun *et al*, 2015; Su *et al*, 2017). For example, increasing the level of Crb-Ex complexes is sufficient to recruit Hpo kinase complexes (including Wts and Mats) to the apical-lateral junctions (Sun *et al*, 2015), while increasing the level of Mer-Kib complexes (by overexpression of Kib) recruits Hpo kinase complexes (including Sav and Wts) to the medial-apical region (Su *et al*, 2017). Thus, apically localised upstream regulators are crucial for the activation of Hpo kinase complexes, yet precisely how they activate the Hpo kinase, and how this activation is physiologically regulated, needs further investigation.

Several different physiological stimuli can regulate the Hippo pathway in *Drosophila*, including mechanical, polarity and hormonal inputs. Mechanical strain (cell shape change) induced by stretching of the apical domain can dilute Crb-Ex and Kib-Mer complexes and reduce Hpo kinase activation (Fletcher *et al*, 2018). Mechanical stress (tension) arising from Rho-mediated actomyosin contractility at E-cadherin-based adherens junctions can recruit the Ajuba protein to inhibit Wts activity (Rauskolb *et al*, 2014; Pan *et al*, 2016, 2018; Alegot *et al*, 2019). Fat-Dachsous cadherins also planar polarise the Dachs myosin to increase junctional tension and inhibit Wts activity in certain tissues (Mao *et al*, 2006, 2011; Bosch *et al*, 2014; Rodrigues-Campos & Thompson, 2014; Vrabioiu & Struhl, 2015). Finally, nutritionally induced circulating growth factors of the Insulin/IGF-1 family signal via the PI3K-Akt pathway to inhibit Hpo activity (Strassburger *et al*, 2012; Fan *et al*, 2013; Borreguero-Munoz *et al*, 2019). Consequently, Hippo signalling is normally weakest in mechanically stretched epithelial cells and normally strongest in densely packed columnar epithelial cells – such as the ovarian follicular epithelium that surrounds the oocyte at stage 10 of oogenesis (Fletcher *et al*, 2018). In addition, Hpo signalling is further elevated upon nutrient deprivation (starvation) via reduced PI3K-Akt signalling in follicle cells (Borreguero-Munoz *et al*, 2019). Precisely how these different physiological inputs are integrated at the level of Hpo kinase activation remains poorly understood.

A common observation is that Hpo kinase complexes at the apical membrane often appear punctate in nature (Sun *et al*, 2015; Su *et al*, 2017; Fletcher *et al*, 2018), suggestive of higher order organisation rather than simple stochiometric complex formation, although the mechanistic basis for this appearance is not understood. One possibility is that Hpo kinase complexes may be localised to endosomal compartments adjacent to the apical membrane, as the upstream regulator Crb is a transmembrane protein that traffics through apically localised Rab11-positive or Rab5-positive endosomes (Blankenship *et al*, 2007; Roeth *et al*, 2009; Fletcher *et al*, 2012; Aguilar-Aragon *et al*, 2020). An alternative possibility is that Hpo signalling complexes may undergo clustering and phase separation to form biomolecular condensates, as observed in other molecular systems (Hyman *et al*, 2014; Banani *et al*, 2017; Case *et al*, 2019). While phase separation is typically associated with cytoplasmic condensates, recent work identified a role for phase separation in transmembrane protein clustering (Banjade & Rosen, 2014; Su *et al*, 2016; Zeng *et al*, 2016; Case *et al*, 2019) and in formation of vertebrate tight junctions (Beutel *et al*, 2019; Schwayer *et al*, 2019), where many upstream Hippo components localise (Karaman & Halder, 2018). Furthermore, in the case of Wnt signalling, cytoplasmic condensates are recruited to the plasma membrane during signal transduction (Gammons & Bienz, 2018; Case *et al*, 2019; Schaefer & Peifer, 2019). Finally, the downstream nuclear transcriptional co-activator YAP was also recently found to undergo phase-separation in the nucleus and cytoplasm (Cai *et al*, 2019). Thus, phase separation of Hpo kinase complexes provides a plausible signalling mechanism.

Here we report that supramolecular assembly of Hpo kinase complexes can also occur by the formation of biomolecular condensates both *in vitro* and *in vivo*. The formation of Hpo condensates is strongly enhanced by starvation-induced loss of PI3K-Akt signalling, or by overexpression of the upstream components Ex and Kib – conditions that activate Hpo signalling. We further identify a key role for the mechanoresponsive spectrin cytoskeleton in promoting clustering of Crb-Ex and Kib-Mer complexes to induce Hpo kinase punctae formation, thereby rendering signal transduction sensitive to mechanical strain. Our findings lead us to propose clustering and phase separation as a unifying biophysical mechanism that enables integration of mechanics, polarity and nutrition by the Hpo signalling pathway.

Results

The Hpo kinase can form cytoplasmic condensates in *Drosophila*

In the *Drosophila* ovarian follicle cell epithelium, Hpo signalling is physiologically activated in densely packed columnar follicle cells that surround the oocyte at stage 9–10 of oogenesis (Fletcher *et al*, 2018) and Hpo signal activation is further enhanced by nutrient deprivation (starvation) which reduces PI3K-Akt signalling (Strassburger *et al*, 2012; Borreguero-Munoz *et al*, 2019). We sought to visualise Hpo kinase activation in this model system using both an endogenously tagged Hpo-YFP knockin allele, to detect bulk Hpo protein, as well as a split-Venus bimolecular fluorescence complementation (BiFC) reporter, in which dimers/multimers of Hpo reconstitute the Venus molecule to induce fluorescence, to detect active Hpo (Fletcher *et al*, 2018). A kinase-dead version of this BiFC reporter (Hpo^{KD}-Venus) can be expressed without inducing ectopic Hpo signalling, avoiding abnormal phenotypes (Fletcher *et al*, 2018). Hpo^{KD}-Venus complexes are most strongly detectable at the apical plasma membrane of densely packed columnar follicle cells at stage 10 of oogenesis, similar to other upstream Hippo pathway components (Figs 1A and EV1A–E; Fletcher *et al*, 2018). Hpo^{KD}-Venus complexes exhibit a punctate appearance at the apical membrane (Fig 1A), which we previously showed overlaps with both Crb-Ex and Mer-Kib complexes in follicle cells (Sherrard & Fehon, 2015; Fletcher *et al*, 2018).

Upon nutrient restriction, or reduced PI3K-Akt signalling, we noticed the striking appearance of large spherical Hpo^{KD}-Venus supramolecular complexes both at the apical membrane and in the cytoplasm (Fig 1A–E; Borreguero-Munoz *et al*, 2019). We confirmed these findings using the endogenously tagged Hpo-YFP reporter line as well as an alternative Hpo-Sav split-Venus BiFC interaction

 © 2023 The Authors

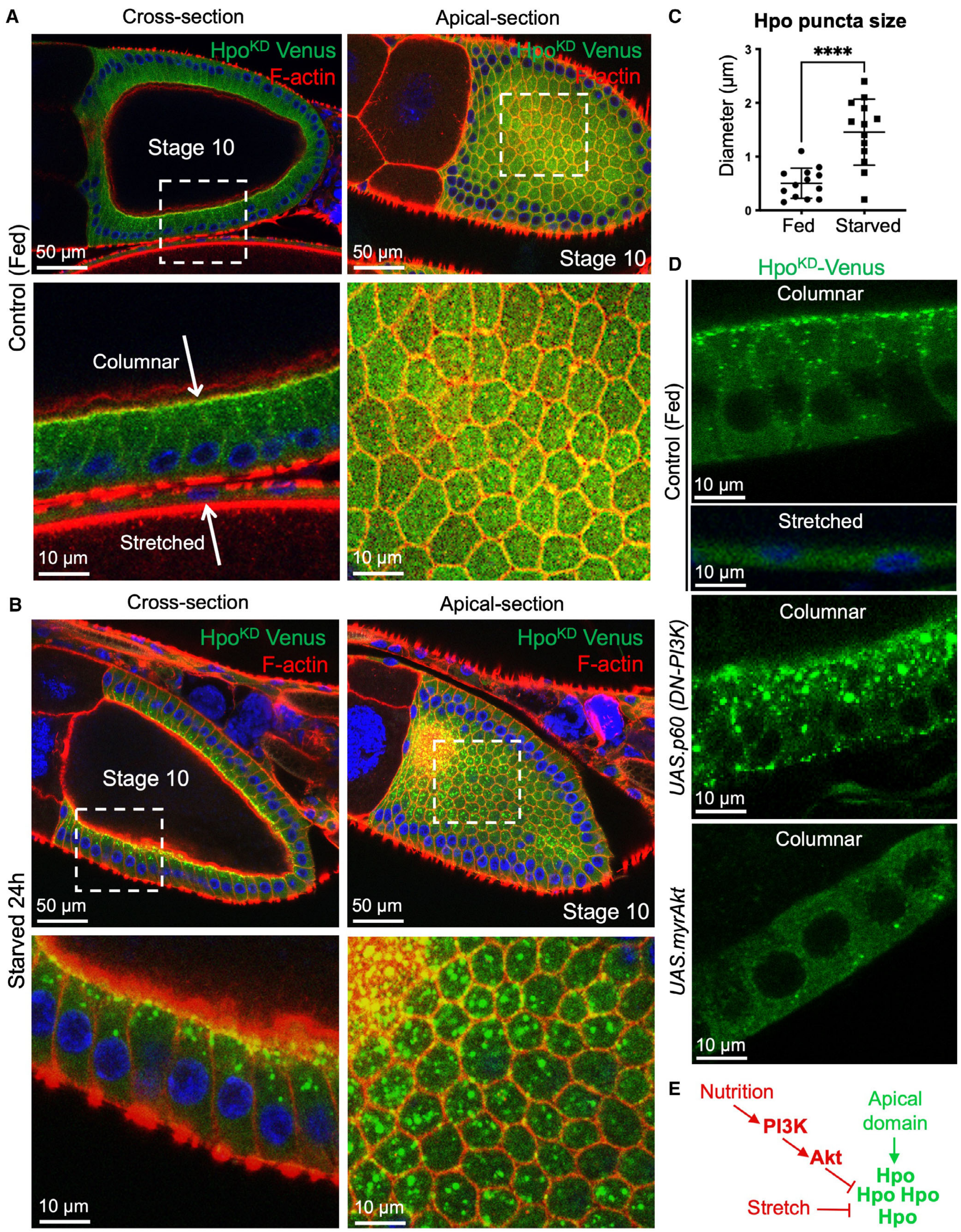
A
Cross-section
Apical-section
HpoKD Venus
F-actin
Stage 10
50 μm
Control (Fed)
Columnar
Stretched
10 μm
B
Starved 24h
C
Hpo puncta size
Diameter (μm)
****
Fed
Starved
D
HpoKD-Venus
Columnar
Stretched
UAS.p60 (DN-PI3K)
UAS.myrAkt
E
Nutrition
PI3K
Akt
Stretch
Apical domain
Hpo
Hpo Hpo
Hpo

Figure 1.

Figure 1. Hpo-Venus can form cytoplasmic condensates in a mechanically- and nutritionally dependent manner.

A *Drosophila* egg chambers are composed of a large oocyte surrounded by a columnar follicle cell epithelium that activates a Hpo kinase bimolecular fluorescence complementation (BiFC) dimerization sensor (HpoKD-Venus; green) apically in small punctae. Note that the Hpo sensor is activated in columnar cells but not in mechanically stretched cells.

B Nutrient restriction (starvation) of adult females for 24 h causes formation of large micron-scale Hpo sensor punctae.

C Quantification of the size of Hpo sensor punctae from (A) and (B). Biological replicates are plotted as individual data points ($n = 13$ fed; $n = 13$ starved), error bars represent one standard deviation from the mean, statistical significance was determined using a *t*-test ****$P < 0.0001$ ***$P < 0.001$ **$P < 0.01$ *$P < 0.05$.

D Inhibition of PI3K by overexpression of a dominant-negative version (the p60 subunit alone) is sufficient to induce formation of large micron-scale Hpo sensor punctae, while activation of Akt by overexpression of a myristylated form of the protein is sufficient to reduce formation of the small apical Hpo sensor punctae that are normally observed in control animals.

E Schematic diagram of pathways regulating formation of supramolecular Hpo kinase punctae *in vivo*.

Source data are available online for this figure.

sensor (Fig EV1F–I). Upon starvation, the endogenous Hpo-YFP forms punctae and formation of these supramolecular complexes is concentration-dependent (Fig EV1J and K). The Hpo^{KD}-Venus complexes are phosphorylated in their activation loop (anti-pT195, equivalent to pT183 in MST1), confirming that they are phosphorylated by endogenous Hpo kinase (Fig EV2A). Furthermore, expression of a kinase-active version of the Hpo-Venus dimerization sensor, which induces signalling, is sufficient to induce large spherical complexes in the cytoplasm even in normally fed animals (Fig EV2A). Overexpression of constitutively active myr-Akt reduced the formation of Hpo^{KD}-Venus complexes (Figs 1D and E, and EV2A–D). These findings show that starvation-induced loss of PI3K-Akt signalling can promote formation of large Hpo kinase complexes, while activation of PI3K-Akt signalling reduces their formation.

To characterise the biophysical nature of these Hpo kinase complexes, we first considered the possibility that they may be bound to autophagosomes or other endosomes. Starvation, or loss of PI3K-Akt signalling, is known to cause reduced TORC1 activity that drives the formation of autophagosomes via activation of the kinase Atg1 (ULK1 in mammals; Noda & Ohsumi, 1998; Arico *et al*, 2001; Scott *et al*, 2004, 2007). To examine this possibility, we made use of an Atg8-mCherry sensor that localises strongly to autophagosomes (Mauvezin *et al*, 2014). We find that the formation of large Hpo^{KD}-Venus complexes appears to precede formation of large autophagosomes in ovarian follicle cells following starvation of adult females, and even when large autophagosomes do form in severely affected egg chambers, they rarely overlap with Hpo kinase complexes (Fig EV2E). Similarly, endosomal markers do not colocalise with Hpo kinase complexes (Fig EV3A and B). These results indicate that large Hpo kinase complexes induced upon nutrient restriction, or reduced PI3K-Akt signalling, are generally distinct from both autophagosomes and endosomes.

We next considered the possibility that large Hpo kinase complexes may instead form by phase separation into biomolecular condensates (Hyman *et al*, 2014; Banani *et al*, 2017; Shin & Brangwynne, 2017; Case *et al*, 2019). Starvation of cells is known to induce phase separation of various condensates in the cytoplasm (van Leeuwen & Rabouille, 2019). Typically, biomolecular condensates can be disrupted by treatment with the compound 1,6-hexanediol, which disrupts phase separation, and enhanced by macromolecular crowding induced by the osmotic withdrawal of water from cells upon treatment with PEG or Sorbitol (Cai *et al*, 2019; McSwiggen *et al*, 2019). Accordingly, we find that treatment of egg chambers with 10% 1,6-hexanediol for 5 min strongly inhibits Hpo^{KD}-Venus punctae from coalescing at the apical membrane of follicle cells (Fig 2A and B). Conversely, treatment of egg chambers with 1 M Sorbitol to induce macromolecular crowding was sufficient to drive the formation of large spherical Hpo^{KD}-Venus complexes, even in well fed animals (Fig 2C). Crowding-induced Hpo^{KD}-Venus complexes are similar in size and number to those induced by starvation of adult females (Fig 2D). Furthermore, the large starvation-induced Hpo^{KD}-Venus complexes do not form in the presence of 10% 1,6-hexanediol (Figs 2E and F, and EV3C). These findings suggest that Hpo kinase punctae display several properties of biomolecular condensates, including phase separation driven by increasing protein concentrations and sensitivity to crowding/hexanediol treatment.

Two further properties of biomolecular condensates include the ability of two condensates to undergo dynamic fusion within the cytoplasm and to exhibit dynamic exchange of proteins between the condensate and the cytoplasm. To test these two key properties, we performed live imaging of Hpo^{KD}-Venus punctae formed under starvation conditions in *Drosophila* follicular epithelium. We observed multiple fusion events between two or three punctae in the cytoplasm of follicle cells over a time-scale of seconds (Fig 2G). In addition, we performed photobleaching of Hpo^{KD}-Venus punctae and measured the recovery of fluorescence by dynamic exchange of protein from the surrounding cytoplasm (Fig 2H). Quantification of fluorescence recovery after photobleaching (FRAP) indicates rapid recovery of punctae over a time-scale of seconds (Fig 2I). Note that FRAP recovery is not able to return to full fluorescence intensity, and this might reflect generalised mobility of the punctae in the *z*-axis that we observed during live-imaging (Fig 2I). Together, these results confirm that Hpo punctae share two key characteristics of biomolecular condensates: dynamic fusion events and dynamic exchange with the cytoplasm.

Endogenously tagged Hpo-YFP also forms puncta apically (Fig 3A), where upstream signalling components are known to localise. Furthermore, puncta formation by endogenous Hpo-YFP is also regulated by both nutritional and mechanical inputs, as well as being sensitive to treatment with 1,6-hexanediol (Figs 3A–D, and EV3D and E). These results provide further support for the notion that phase separation of endogenous Hpo signalling complexes is a physiological signalling mechanism induced by upstream apical cues in *Drosophila*.

Hpo kinase condensates can be nucleated by upstream pathway components in *Drosophila*

A fourth key property of biomolecular condensates is their ability to be “nucleated” by regulatory proteins in a concentration-dependent

© 2023 The Authors

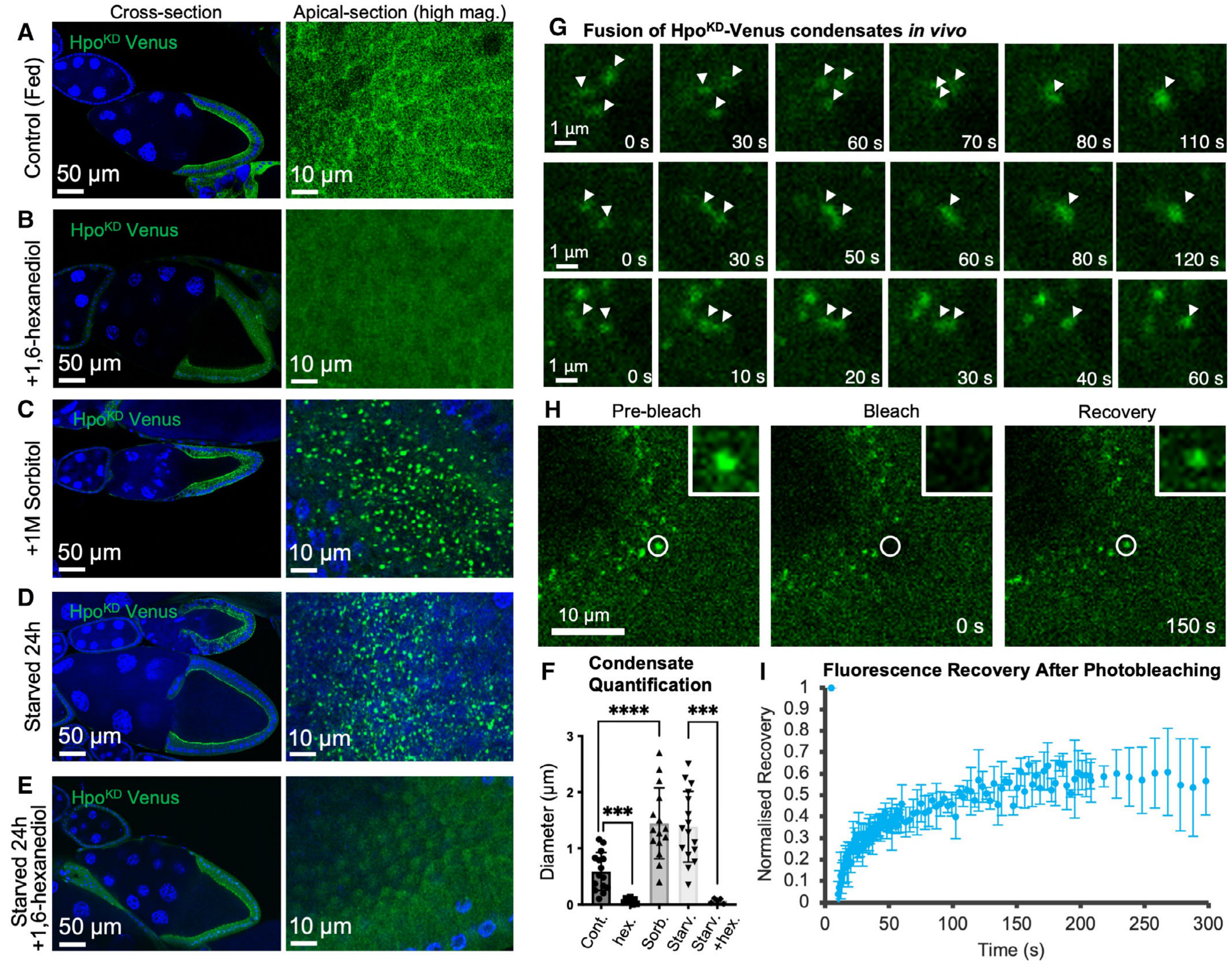

Figure 2. Hpo-Venus punctae are sensitive to 1,6-hexanediol, enhanced by macromolecular crowding, and undergo fusion and dynamic exchange with the cytoplasm.

- A *Drosophila* egg chambers at stages 9/10 of oogenesis expressing a Hpo kinase dimerization sensor that forms small punctae at the apical domain of the follicle cell epithelium that surrounds the oocyte.
- B Treatment of egg chambers with 1,6-hexanediol (10%) for 5 min abolishes formation of Hpo sensor punctae.
- C Treatment of egg chambers with 1 M Sorbitol to osmotically withdraw water, and thereby induce macromolecular crowding, is sufficient to induce formation of large micron-scale Hpo sensor punctae.
- D Nutrient restriction (starvation) of adult females for 24 h induces large micron-scale Hpo sensor punctae that are similar in size to those in (C).
- E Treatment of egg chambers from adult females starved for 24 h with 1,6-hexanediol (10%) for 5 min abolishes formation of Hpo sensor punctae.
- F Quantification of the size of Hpo sensor punctae from (A–E). Biological replicates are plotted as individual data points ($n = 15$ control; $n = 8$ hex; $n = 15$ sorb; $n = 16$ starv; $n = 5$ starv + hex), error bars represent one standard deviation from the mean, statistical significance was determined using a t-test ****$P < 0.0001$ ***$P < 0.001$ **$P < 0.01$ *$P < 0.05$.
- G Live-imaging of HpoKD-Venus (green) condensates within follicle cells at high magnification reveals fusion events, occurring over the timescale of seconds. White arrowheads point to individual punctae undergoing fusion.
- H Fluorescence Recovery After Photobleaching (FRAP) experiments reveal that photobleaching of individual condensates is followed by rapid recovery after 150 s.
- I Quantification of FRAP recovery as a fraction of the initial fluorescence intensity. The rapid initial slope of recovery suggests dynamic exchange between the condensate and the cytoplasmic pool of the proteins. Temporal averages are plotted as individual data points ($n > 3$ biological replicates), error bars represent one standard deviation from the mean.

Source data are available online for this figure.

manner (Hyman *et al*, 2014; Banani *et al*, 2017). Since Hpo signalling can be induced simply by raising the expression level of the upstream regulators Ex or Kib, we tested whether overexpression of these proteins would be sufficient to induce Hpo kinase punctae in the cytoplasm. In the case of Ex, we find that endogenously tagged Ex-YFP exhibits a similar or overlapping punctate pattern to that

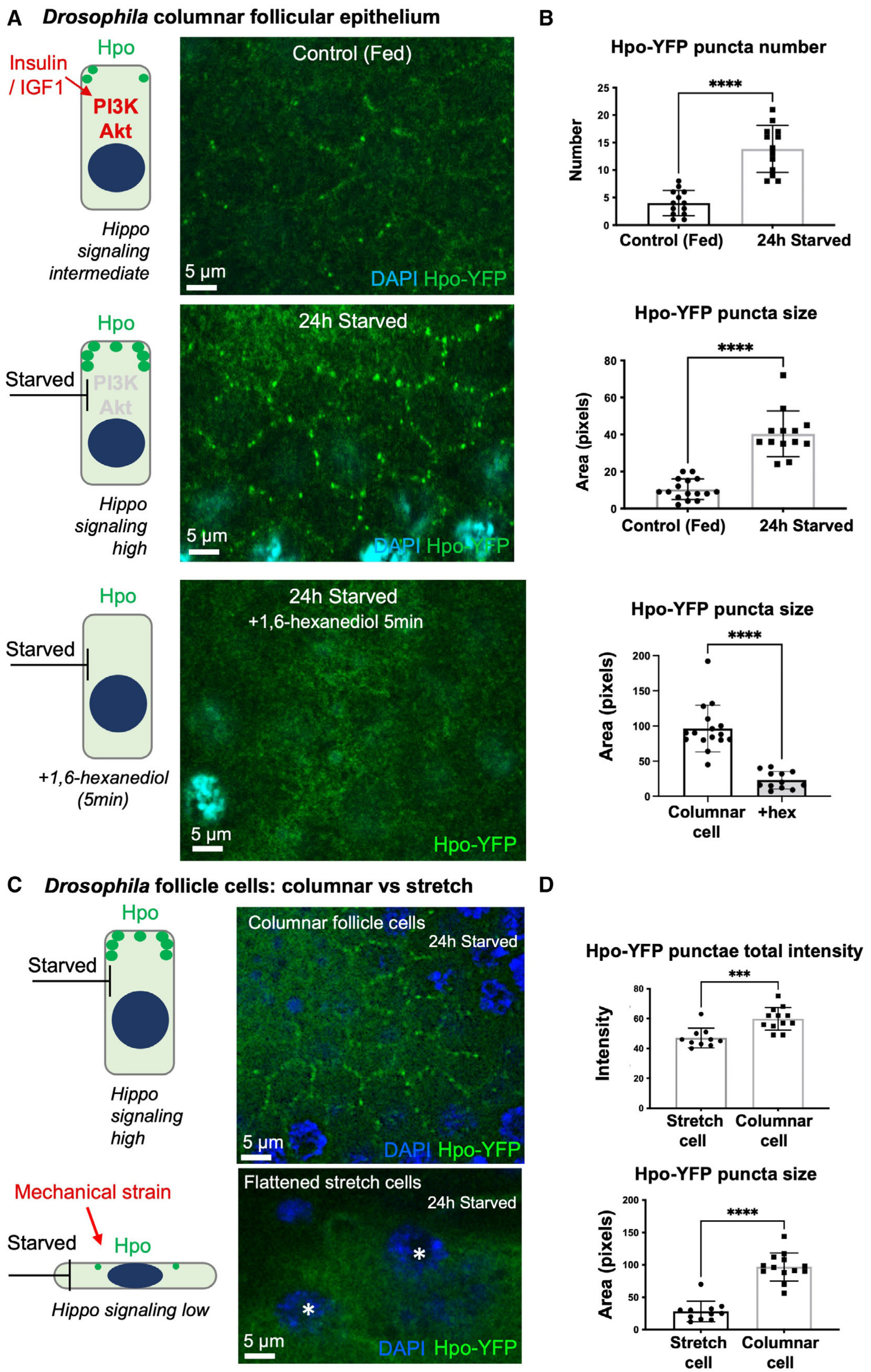

Figure 3.

© 2023 The Authors

Figure 3. Endogenous Hpo-YFP punctae are regulated by nutritional and mechanical stimuli.

A Apical cross-sections of *Drosophila* ovarian follicle cell epithelia at high magnification, showing accumulation of Hpo-YFP punctae at the apical domain, which is strongly enhanced upon 24 h starvation to reduce Insulin/IGF-1 signalling. Hpo puncta are lost following a short treatment with 1,6-hexanediol. Samples were fixed before imaging.

B Quantification of Hpo-YFP puncta number and size in (A). Biological replicates are plotted as individual data points ($n > 13$ for all samples), error bars represent one standard deviation from the mean, statistical significance was determined using a *t*-test ****$P < 0.0001$ ***$P < 0.001$ **$P < 0.01$ *$P < 0.05$.

C Apical cross-sections of *Drosophila* ovarian follicle cell epithelia at high magnification, showing loss of Hpo-YFP punctae upon mechanical strain in "stretch cells". The *Drosophila* follicle cell epithelium at stage 10 is composed of anterior stretch cells (flat) and columnar cells. Endogenously tagged Hpo punctae are strongly enriched at the apical domain of columnar cells but not in flattened stretch cells. White asterisks demarcate nuclei of individual stretch cells.

D Quantification of Hpo-YFP total intensity and size in (C). Biological replicates are plotted as individual data points ($n > 10$ for all samples), error bars represent one standard deviation from the mean, statistical significance was determined using a *t*-test ****$P < 0.0001$ ***$P < 0.001$ **$P < 0.01$ *$P < 0.05$.

formed by Hpo^{KD}-Venus punctae at the apical membrane domain (Fig 4A and B) and that Ex overexpression is sufficient to drive formation of large spherical Ex and Hpo^{KD}-Venus punctae in the cytoplasm (Fig 4C and D). The Ex and Hpo^{KD}-Venus proteins co-localise in the same punctae in the cytoplasm, which do not contain the Crb transmembrane protein, which largely remains at the apical membrane (Fig 4E). Furthermore, the Ex-induced Hpo^{KD}-Venus complexes do not co-localise with endosomes through which Crb is known to traffic, such as Rab5, Rab7, or Rab11 (Fig EV3A). The Ex-induced punctae also stain positively for active phosphorylated Wts/Lats (p-Wts), consistent with the notion that they are actively signalling via recruitment of the endogenous Hpo kinase (Fig 4F). In the case of Kib, we find that overexpression of Kib-GFP was sufficient to induce large spherical punctae of Hpo^{KD}-Venus and Kib-GFP itself (Fig 4G and H). These results suggest that Ex or Kib overexpression is sufficient to promote formation of active Hpo kinase condensates.

Crb, Ex, Kib and Mer interact with the spectrin cytoskeleton, which forms a spring-like mesh that links F-actin to the plasma membrane, to regulate Hpo signalling in *Drosophila* (Medina *et al*, 2002; Deng *et al*, 2015, 2020; Fletcher *et al*, 2015). Accordingly, we find that silencing of *alpha-Spectrin* (*α-Spec*) expression by *tj.Gal4*-driven *UAS.α-Spec RNAi* strongly reduces apical localisation of Hpo^{KD}-Venus complexes (Fig EV4A and B). The localisation of the apical spectrin cytoskeleton can be readily visualised with a YFP-tagged betaHeavy-Spectrin ($ß_H$-Spec-YFP) allele (Fig EV4C). In addition, mutant clones of *α-Spec* induced in columnar follicle cells at stage 10 cause a loss of Kib clusters from the medial-apical region (Fig EV4D). This loss of Kib clustering is not due to reduced Kib levels, which are actually transcriptionally induced as a consequence of the loss of Hpo signalling that occurs in *α-Spec* mutant clones (Fig EV4E). Furthermore, silencing of *kib* itself via *tj.Gal4*-driven *UAS.Kib RNAi* strongly reduces apical Hpo^{KD}-Venus punctae (Fig EV4F). These findings further validate the Hpo^{KD}-Venus sensor as a physiological readout of Hippo signalling whose activity depends strictly on endogenous upstream Hippo pathway components. In addition, these results demonstrate an important role for the spectrin cytoskeleton in regulating clustering of Hpo kinase complexes by apical Crb-Ex and Mer-Kib to induce signalling.

The major upstream Hpo pathway components all contain intrinsically disordered regions, identified using two independent algorithms for disorder prediction (Fig EV4G). Molecular modelling of these proteins suggests that such unstructured regions could engage in multivalent interactions, in part by the presence of multiple PPXY motifs that mediate PPXY-WW interaction networks (Fig EV4H). Intrinsically disordered protein regions are characteristic of both the Crb-Ex network at apical junctions and the Mer-Kib complexes at the medial-apical region/microvilli and at apical junctions (Fig EV4H). Notably, the long intrinsically disordered regions in both Ex and Kib may enable these proteins to induce condensate formation in the cytoplasm when overexpressed (Fig EV4H). Thus, the key upstream regulators of Hippo signalling can promote the formation of large Hpo kinase punctae as well as inducing signal transduction, suggesting that these two properties may be linked.

Hpo/MST kinase condensates can be nucleated by upstream pathway components in human cells

Endogenous human Hpo pathway components are typically found in very small cytoplasmic puncta by immunostaining in flattened human cells in culture, which reflects a cellular state in which signalling activity is low (Fig 5A). Activation of signalling by transfecting Caco2 epithelial cells with upstream apical Hpo pathway components, as shown for human Kibra (KIB), promotes formation of large spherical cytoplasmic puncta (Fig 5B), with these puncta incorporating active Hpo kinase as indicated by pLATS immunostaining (Fig 5B) and co-localization with GFP-LATS1 (Fig EV5A). The number and size of cytoplasmic puncta induced by KIB expression increases linearly with the concentration of KIB in the cell (Fig EV5B). Crucially, the number and size of KIB puncta, and their ability to activate pLATS, depend upon the presence of two intrinsically disordered regions (IDRs) within KIB (Fig 6A–D). Thus, the ability of KIB to activate signalling depends upon its IDRs and its ability to promote phase separation in the cytoplasm.

Despite the key nucleating role of upstream pathway components, overexpression of the Hpo kinase itself is sufficient to induce both signalling and formation of punctae in *Drosophila*, which can be observed in the cytoplasm far from the apical membrane where the upstream pathway components normally concentrate (Fig 1A–D). Thus, our results predict that the core Hpo-Sav complex alone may have the capacity to phase separate without upstream pathway components. To test the notion that Hpo-Sav complexes themselves are able to induce phase separation at sufficiently high concentrations, we sought to analyse purified complexes *in vitro*. We first expressed and purified a soluble complex of human mYFP-SAV1: MST2. We find that the complex undergoes phase separation to form distinct puncta in a concentration-dependent manner and these puncta can be disrupted by 1,6-hexanediol (Fig EV5C). Additionally, fewer condensates are observed for complexes containing a ΔN variant of SAV1 that lacks the predicted disordered regions (Fig EV5B and C). In cell culture, expression of monomeric YFP-tagged SAV1 together with mCherry-tagged MST2 was sufficient to

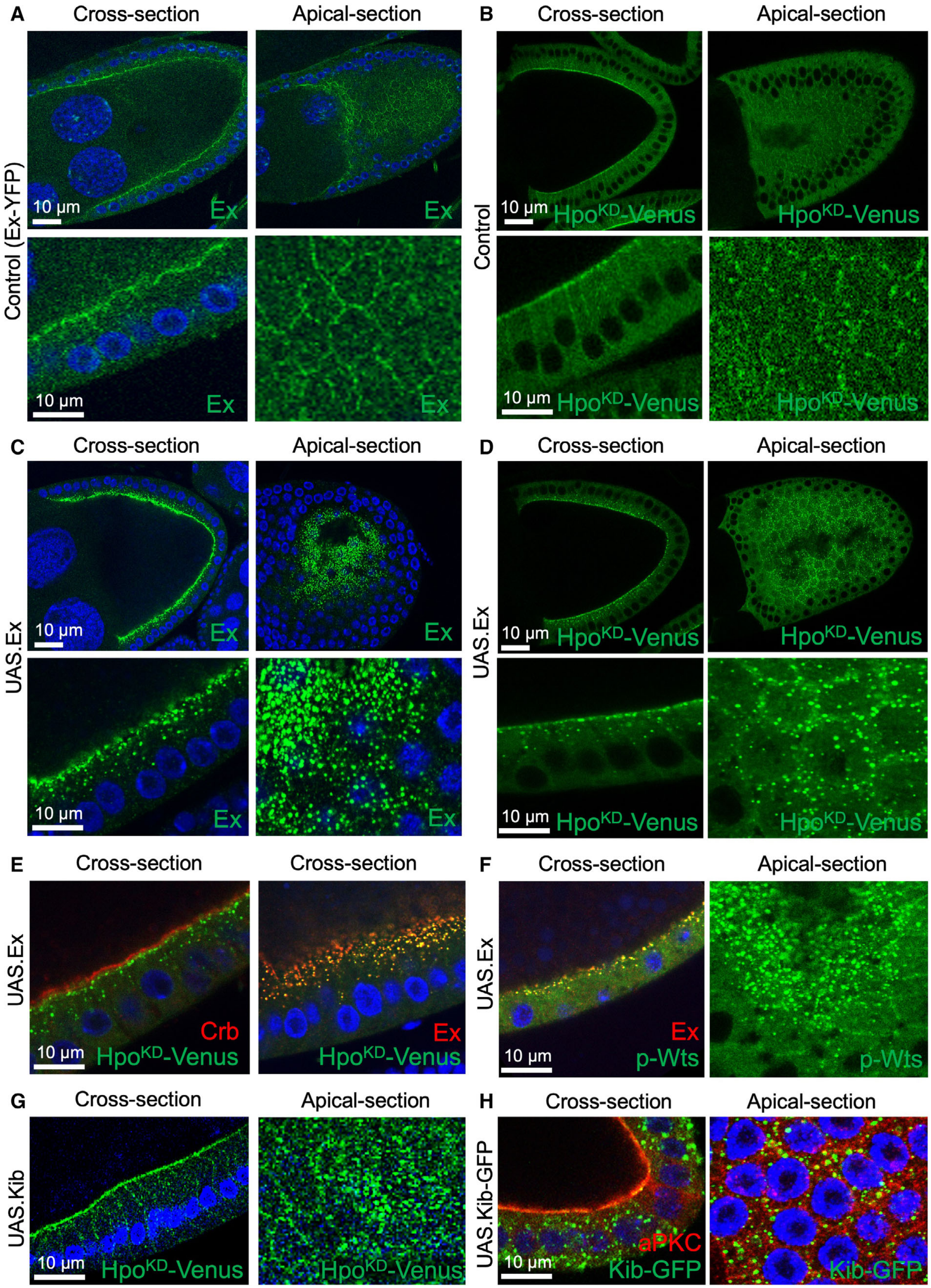

Figure 4.

© 2023 The Authors

Figure 4. Expanded & Kibra overexpression induces formation of large Hpo punctae in the cytoplasm.

A Expanded (Ex; endogenously tagged with YFP) localises to the apical domain of columnar follicle cells at stages 9/10 of oogenesis.
B The Hpo dimerization sensor localises in a similar pattern to Ex.
C Overexpression of Ex is sufficient to induce formation of large micron-scale condensates in the cytoplasm.
D Overexpression of Ex is sufficient to recruit the Hpo sensor into large micron-scale condensates in the cytoplasm.
E Condensates induced by high levels of Ex in the cytoplasm co-localise with the Hpo sensor but do not co-localise with Crb, which remains apical.
F Condensates induced by high levels of Ex in the cytoplasm immunostain positively for phosphorylated Wts.
G Moderate overexpression of untagged Kib is sufficient to induce the formation of large micron-scale Hpo^{KD}-Venus condensates that primarily localise apically. DAPI marks nuclei (blue).
H Overexpression of Kib-GFP with the Gal4/UAS system is sufficient to induce formation of large micron-scale cytoplasmic condensates.

Source data are available online for this figure.

form punctae in which both proteins co-localise (Fig EV5D and E). These results demonstrate that MST2-SAV1 complexes can form biomolecular condensates either *in vitro* or upon overexpression in cultured cells.

We next sought to examine whether endogenous Hpo signalling complexes form punctae in polarised human epithelial cells. Endogenous pLATS-containing punctae localise to the apical surface in 2D cultured Caco2 epithelial cells, particularly with increasing cell density, and even more strikingly upon 3D culture of Caco2 intestinal epithelial cells, which strongly activates signalling and therefore causes YAP retention in the cytoplasm (Fig 7A). In 3D cultured intestinal organoids, which have a highly columnar morphology, pLATS-containing punctae localise strongly to an apical ring (Fig 7B) and are also sensitive to treatment for 5 min with 1,6-hexanediol (Fig EV6A and B). Similarly, in biopsies of human colon cancer, endogenous Hpo pathway components concentrate at the apical surface as detected by immunostaining of histological sections (Fig EV6C). Together, these results indicate that endogenous mammalian Hpo signalling complexes can cluster and form condensates that contain active, phosphorylated, LATS kinase at the apical domain of polarised epithelial cells. Thus, mammalian Hpo signalling complexes can undergo phase separation in a similar manner to those in *Drosophila* (Fig EV6D).

Next, we asked whether physiological regulation of Hpo pathway punctae formation by mechanical strain is conserved between *Drosophila* and mammalian cells. We find that stretching of the apical domain (mechanical strain that increases apical area) is sufficient to reduce endogenous pLATS punctae formation in mammalian intestinal epithelial organoids. pLATS-containing signalling punctae are readily visible at the apical domain of highly columnar cells in mammalian organoids, whereas Hpo signalling punctae are smaller and dimmer when cells are stretched into a flattened morphology, when Hpo signalling is reduced and YAP becomes nuclear localised (Fig 7C and D). This observation indicates that mechanical regulation of Hpo kinase complex formation and signalling at the apical domain is a fundamentally conserved regulatory principle of Hpo signalling.

Finally, we sought to test whether growth factor signalling via PI3K-Akt is sufficient to limit endogenous pLATS punctae formation in mammalian intestinal epithelial organoids, similar to its role in *Drosophila*. We find that the intensity of pLATS punctae at the apical domain is reduced upon activation of Akt by treatment of intestinal organoids with the intestinal growth factor neuregulin (NRG1), which thereby increases YAP nuclear localisation (Fig 8A–D). We selected the growth factor NRG1 because previous evidence indicates that it is a more potent growth factor and activator of pAkt than other growth factors in intestinal organoids (Jarde *et al*, 2020). Our results confirm that growth factor signalling via PI3K-Akt inhibits Hpo signalling punctae formation and signalling in both *Drosophila* and mammals and is a second fundamentally conserved regulatory principle of Hpo signalling.

Discussion

Our results indicate that Hpo kinase complexes undergo clustering and phase separation to form biomolecular condensates and suggest condensation as an important mechanism of Hpo signalling. Phase separation into biomolecular condensates is emerging as an important organising principle for compartmentalisation of the cytoplasm and nucleus (Hyman *et al*, 2014; Zhu & Brangwynne, 2015; Banani *et al*, 2017; Shin & Brangwynne, 2017); and recent evidence supports a further role for phase separation in clustering plasma membrane proteins (Li *et al*, 2012; Banjade & Rosen, 2014; Su *et al*, 2016; Tracy *et al*, 2016), including vertebrate tight junctions (Beutel *et al*, 2019; Schwayer *et al*, 2019). In contrast, the possible functional role of biomolecular condensates in signal transduction from the plasma membrane is only beginning to be appreciated (Su *et al*, 2016; Case *et al*, 2019), with Wnt signalling one clearly demonstrated example (Gammons & Bienz, 2018; Case *et al*, 2019; Schaefer & Peifer, 2019). In the case of the Hippo pathway, the downstream nuclear effector YAP was recently shown to undergo phase separation, which may influence its transcriptional activity in the nucleus (Cai *et al*, 2019). However, a role for phase separation in activation of the core Hpo kinase cassette was not previously explored.

Consistent with our observation that Hpo kinase complexes can form condensates *in vitro* and *in vivo*, several components of these complexes (Ex, Kib, Hpo, Sav and Wts) contain intrinsically disordered regions, and undergo multivalent interactions (including via a network of PPXY motif - WW domain binding), both of which are fundamental drivers of polymeric clustering and phase separation in other systems (Li *et al*, 2012; Banjade & Rosen, 2014; Banani *et al*, 2017; Kato & McKnight, 2017; Case *et al*, 2019; Schaefer & Peifer, 2019; Bienz, 2020). While overexpression of Ex or Kib can induce the formation of large spherical Hpo kinase punctae in the cytoplasm, the endogenous Ex and Kib proteins localise to the apical plasma membrane, as Crb-Ex complexes (at apical cell–cell junctions) or Mer-Kib complexes (at the medial-apical region as well as at apical cell–cell junctions) to cluster Hpo kinase complexes. These findings are consistent with recent observations that phase separation can occur via clustering of proteins at the inner surface of the plasma membrane (Case *et al*, 2019), including vertebrate tight

© 2023 The Authors

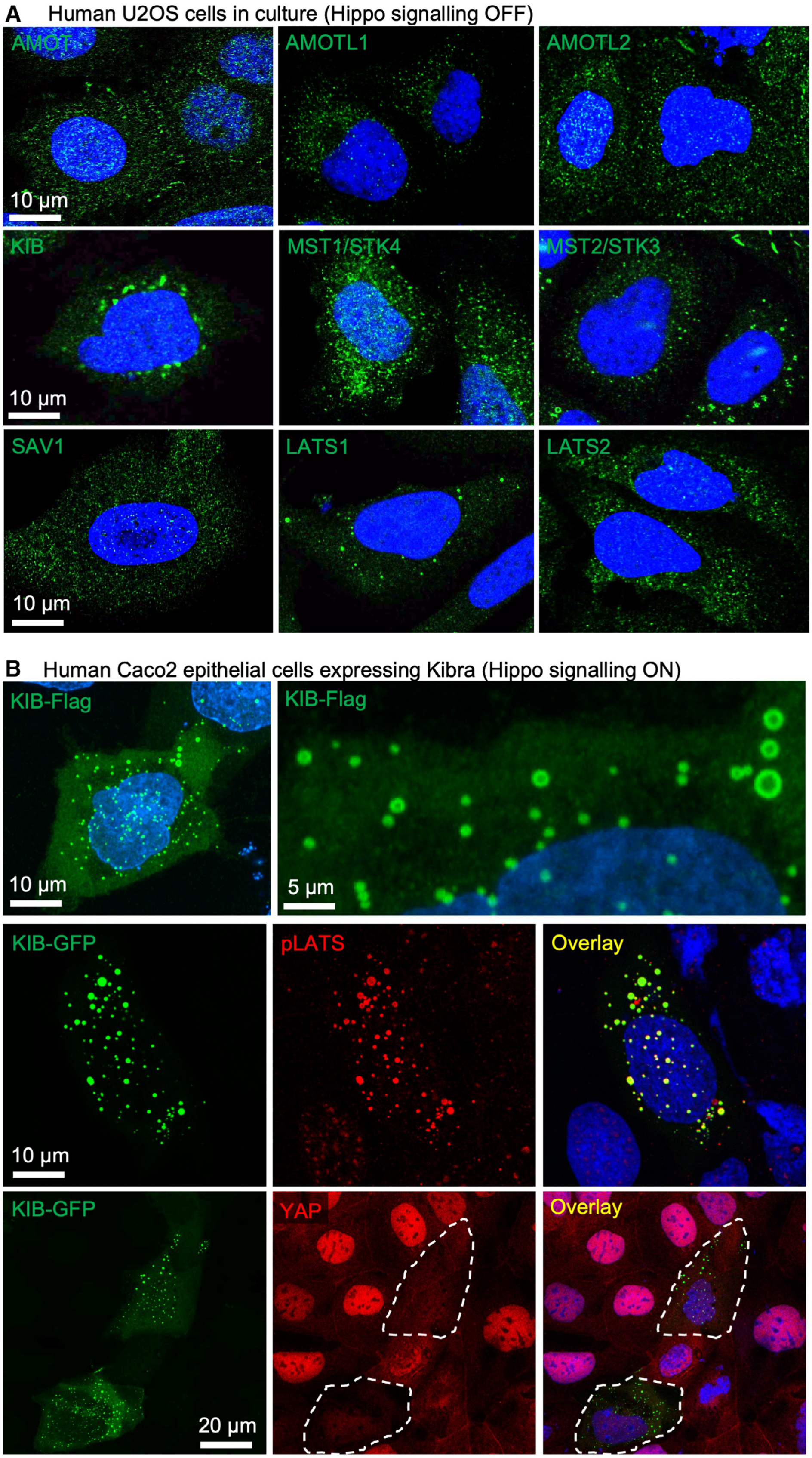

Figure 5.

 © 2023 The Authors

Figure 5. Human Kibra overexpression induces formation of large pLATS-containing punctae in the cytoplasm and induces signalling.

A Human U2OS cells in culture immunostained for endogenous Hippo pathway components reveals punctate subcellular localisation within the cytoplasm.

B Ectopic expression of Flag-tagged human Kibra (KIB-Flag) generates large spherical punctae in the cytoplasm of human Caco2 epithelial cells (top). Co-localisation of KIB-GFP with active phosphorylated LATS (pLATS) in the cytoplasm of human Caco2 epithelial cells (middle). Note that cells expressing KIB-GFP have increased Hippo signalling as indicated by reduced levels of nuclear YAP localisation (bottom). Dashed lines demarcate transfected cells.

Source data are available online for this figure.

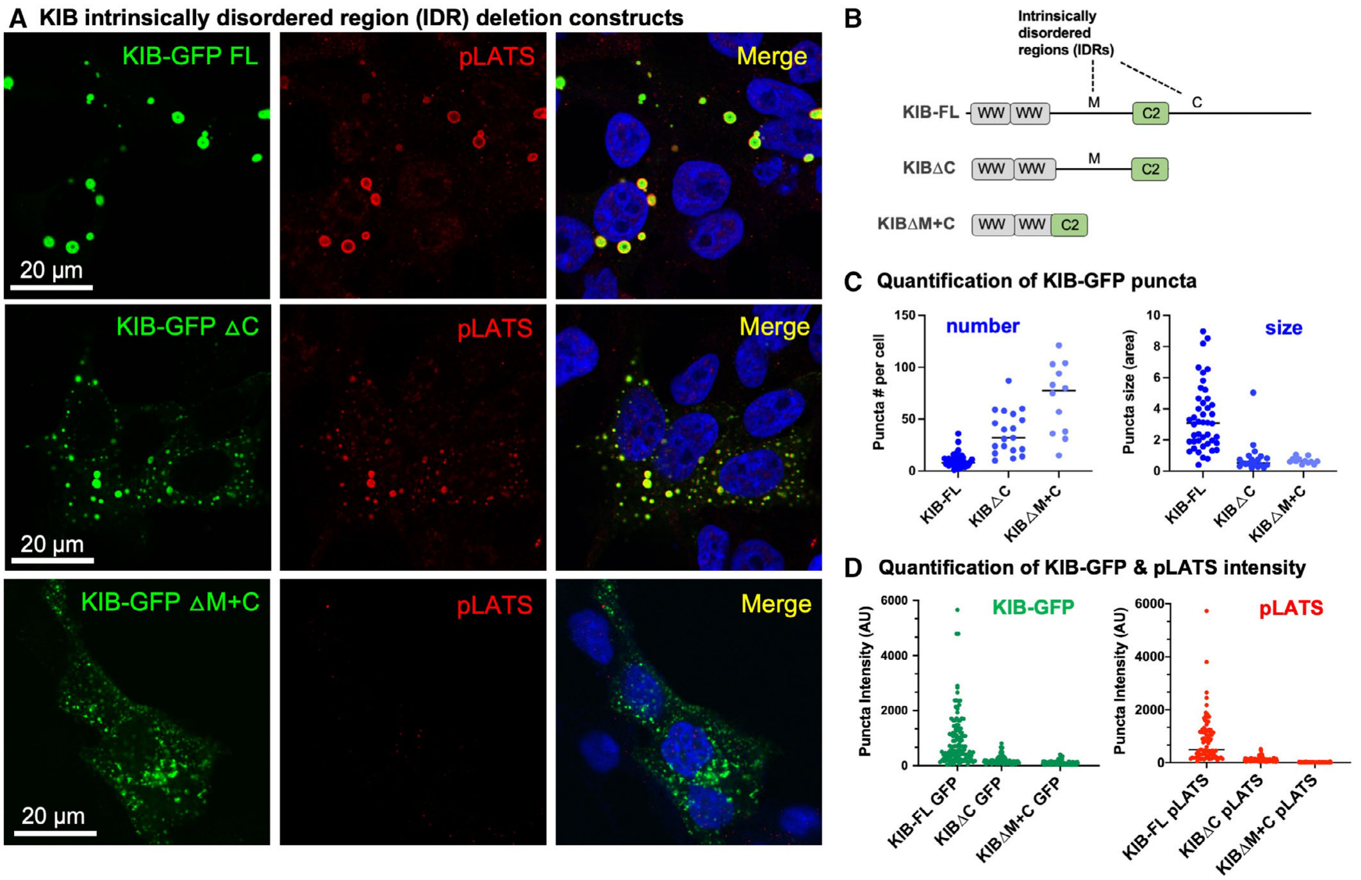

Figure 6. Intrinsically disordered regions (IDRs) are required for KIB to form large spherical punctae and to activate pLATS.

A Expression of KIB-GFP, KIBΔC-GFP or KIBΔM + C-GFP in human HEK293T cells and co-staining for pLATS. DAPI marks nuclei in blue.

B Schematic diagram of KIB intrinsically disordered regions (M) and (C) and the ΔC and ΔM + C deletion constructs.

C Quantification of KIB-GFP puncta number and size showing dependence upon the IDRs. Biological replicates are plotted as individual data points ($n > 12$ for all samples), error bars represent one standard deviation from the mean, statistical significance was determined using a *t*-test ****$P < 0.0001$ ***$P < 0.001$ **$P < 0.01$ *$P < 0.05$.

D Quantification of KIB-GFP and pLATS intensity showing dependence of both condensate formation and signalling upon the IDRs. Biological replicates are plotted as individual data points ($n > 40$ for all samples), error bars represent one standard deviation from the mean, and statistical significance was determined using a *t*-test ****$P < 0.0001$ ***$P < 0.001$ **$P < 0.01$ *$P < 0.05$.

Source data are available online for this figure.

junctions (Beutel *et al*, 2019; Schwayer *et al*, 2019), where many upstream Hippo components localise (Karaman & Halder, 2018). Notably, phase separation at vertebrate tight junctions is driven by the ZO-1 protein, which in *Drosophila* localises to adherens junctions, so cannot explain the behaviour of *Drosophila* Hpo kinase complexes at the apical plasma membrane. Thus, Hpo kinase complexes have an autonomous capacity to cluster, independently of ZO-1, with either Crb-Ex or Mer-Kib at the apical membrane.

Genetic analysis of the Crb-Ex and Mer-Kib complexes indicates that they function semi-redundantly to activate Hpo signalling in *Drosophila* (McCartney *et al*, 2000; Hamaratoglu *et al*, 2006; Baumgartner *et al*, 2010; Genevet *et al*, 2010; Yu *et al*, 2010). Genetic analysis also shows that the spectrin cytoskeleton promotes signalling by both Crb-Ex and Mer-Kib complexes in all tissues (Fletcher *et al*, 2015) as well as apical membrane trafficking and formation of microvilli (Khanal *et al*, 2016). Our results show that the spectrin

© 2023 The Authors

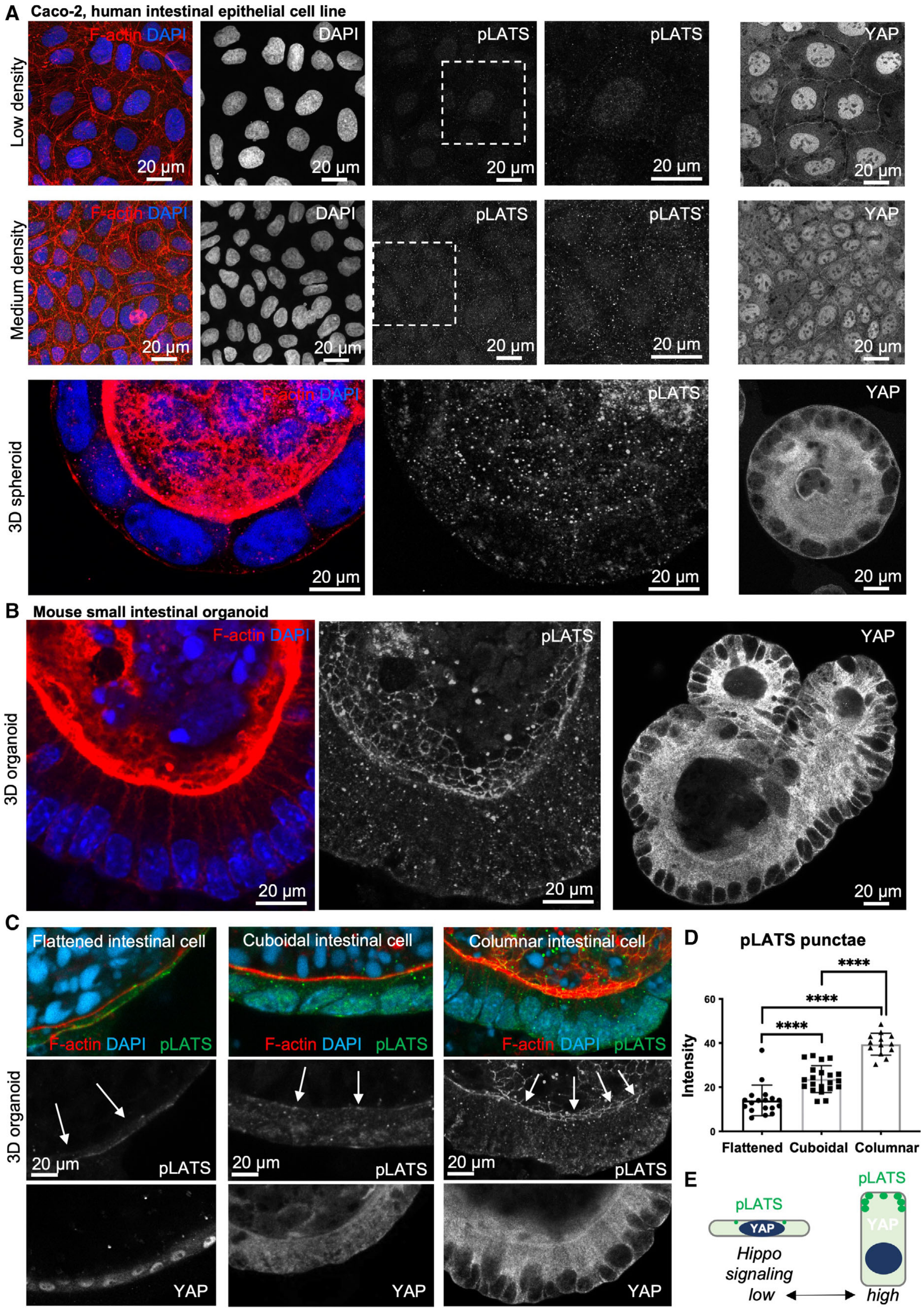

Figure 7.

 © 2023 The Authors

Figure 7. Mechanical strain inhibits the formation of pLATS-containing punctae and drives YAP nuclear localisation.

A Human Caco2 intestinal epithelial cells are relatively flat when cultured as cell lines in 2D (Caco-2 cells, top, middle) and cuboidal when cultured in 3D (spheroid, bottom). Punctae of pLATS (green) are strongly enriched at the apical domain of cuboidal cells grown in 3D culture but not in flattened cells grown in 2D culture. Note the increased apical enrichment in medium-density culture in 2D, where cells become more cuboidal than at low density. Phalloidin staining marks the actin cytoskeleton in red. DAPI marks nuclei in blue.

B Mouse small intestinal organoids exhibit highly columnar cells which feature a striking concentration of pLATS-containing punctae into an apical ring and high levels of signalling (YAP becomes largely cytoplasmic).

C Mouse small intestinal organoids can become stretched upon growth as inflated spheres, which leads to mechanical strain in epithelial cells and reduced pLATS localisation apically, which correlates with reduced Hippo signalling (leading to increased YAP nuclear localisation). White arrows highlight the apical domain of epithelial cells.

D Quantification of pLATS punctae total intensity in mouse small intestinal organoids from (C). Biological replicates are plotted as individual data points ($n > 17$ for all samples), error bars represent one standard deviation from the mean, statistical significance was determined using a t-test ****$P < 0.0001$ ***$P < 0.001$ **$P < 0.01$ *$P < 0.05$.

E Schematic diagram of YAP nuclear localisation in mechanically stretched cells and YAP cytoplasmic localisation in columnar epithelial cells.

Source data are available online for this figure.

cytoskeleton is also essential to promote the formation of Kib clusters at the membrane and the formation of Hpo kinase punctae apically. Thus, in the follicular epithelium, the spectrin cytoskeleton acts apically with Crb-Ex and Mer-Kib to regulate Hpo signalling, where it forms a densely packed network at both apical cell–cell junctions and at the microvillar terminal web. In epithelial tissues that lack conspicuous microvilli, such as the developing *Drosophila* wing epithelium, the apical spectrin cytoskeleton localises primarily to apical cell–cell junctions, where it overlaps with both Crb-Ex and Mer-Kib complexes – which largely co-localise in an apical ring in this tissue (Fletcher *et al*, 2015). Despite these minor differences between the wing epithelium and follicular epithelium, both tissues respond similarly to mechanical strain (stretching epithelial cells into a flattened shape that expands the apical surface area), with Crb-Ex and Mer-Kib complexes becoming diluted and Hpo kinase complexes becoming de-clustered to reduce Hpo signalling and thereby activate Yki-driven transcriptional responses (Fletcher *et al*, 2018). Thus, the upstream pathway components promote clustering of Hpo kinase punctae at the apical domain and, by virtue of the spectrin cytoskeleton being a mechanically deformable network, these punctae respond to mechanical strain.

Mechanical strain is therefore well established as a key physiological signal regulating Hpo signalling and, importantly, our data suggest that formation of endogenous Hpo biomolecular condensates is regulated by mechanical strain. We have demonstrated that endogenous Hpo-YFP punctae become de-clustered in response to flattening of developmentally stretched epithelial cells in *Drosophila*. Our data also show that the ability of mechanical strain to regulate Hpo kinase punctae formation and signalling is conserved between *Drosophila* and mammalian epithelia. We found that endogenous pLATS-containing punctae form an apical ring in columnar epithelial cells of mouse intestinal organoids and are de-clustered upon flattening of these cells, which also causes re-localisation of YAP to the nucleus. These observations suggest that regulation of clustering and phase separation of Hpo complexes by mechanical strain is conserved across animal species.

In addition to sensing mechanical strain, Hippo signalling can also respond to nutritional input, which induces hormonal Insulin/IGF-1 Receptor (InR) signalling via the PI3K-Akt pathway (Strassburger *et al*, 2012; Fan *et al*, 2013; Borreguero-Munoz *et al*, 2019). Starvation reduces PI3K-Akt activity and thereby leads to increased Hippo signalling to restrict cell proliferation (Borreguero-Munoz *et al*, 2019). Our results show that inhibition of PI3K-Akt promotes the formation of large Hpo kinase punctae, mimicking the effect of starvation. Ectopic activation of Akt is sufficient to prevent the formation of large Hpo kinase punctae under conditions of starvation. Thus, the physiological effects of nutrient restriction on Hpo kinase condensates are mediated by Akt. This role of Akt appears to be direct, rather than mediated via the Rheb-TORC1 pathway, as ectopic activation of Rheb-TORC1 signalling is not sufficient to prevent the formation of large Hpo kinase condensates under conditions of starvation. These findings are consistent with our previous observation that Hippo signalling is unaffected in *rheb* mutant clones in the *Drosophila* ovarian follicle cell epithelium (Borreguero-Munoz *et al*, 2019). Importantly, our data confirm that growth factor regulation of Hippo signalling is a conserved principle between *Drosophila* and mammalian epithelial cells. We have shown that activation of Akt upon growth factor stimulation of mouse intestinal organoids is sufficient to reduce formation of pLATS-containing apical punctae and consequently promote re-localisation of YAP to the nucleus. Thus, the results suggest that regulation of Hpo clustering and phase separation by growth factor-induced PI3K-Akt signalling are conserved across animal species.

The human Hpo kinase complex can be observed to undergo phase separation *in vitro*, with purified MST2-SAV1 condensates, as well as observed to form punctae in the cytoplasm. In human epithelial cells, Hpo signalling punctae containing active pLATS form at the apical domain. Ectopic expression of KIB generates large micron-scale punctae in cells that activate pLATS and also induce signalling to retain YAP in the cytoplasm. The ability of KIB to form these large spherical punctae depends upon its intrinsically disordered regions (IDRs), in accordance with the notion that punctae formation is mediated by phase separation. In human epithelial cells, endogenous pLATS-containing punctae form strongly at the apical domain of densely packed cells in culture, while forming less frequently and at smaller sizes in flattened epithelial cells. In human cells that completely lack an apical domain (such as U2OS cells), Hippo pathway components form much smaller and dispersed clusters in the cytoplasm. Thus, as in *Drosophila*, the formation of human Hpo signalling punctae correlates with the clustering of upstream pathway components and is able to control Hpo signalling activity to YAP in human cells. Collectively, our results support a "Hpo signalosome" model in which clustering and phase separation promotes kinase domain proximity to drive autophosphorylation and signal transduction (Fig EV6D).

© 2023 The Authors

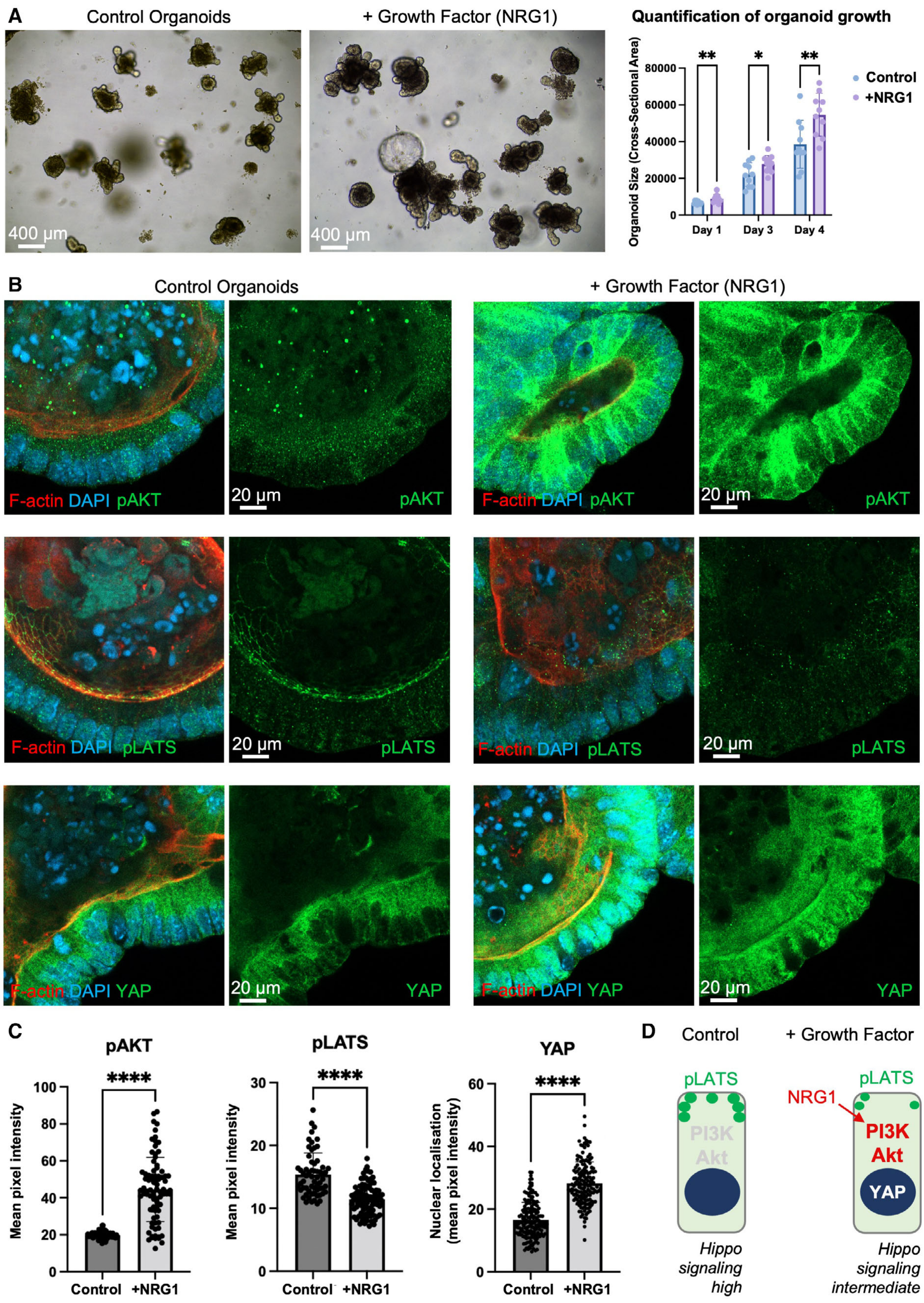

Figure 8.

© 2023 The Authors

Figure 8. Growth Factor signalling via Akt inhibits pLATS punctae and increases nuclear YAP.

A Murine small intestinal organoids cultured in control medium or medium supplemented with the NRG1 growth factor, which stimulates organoid growth (see graph, right). Biological replicates are plotted as individual data points ($n > 9$ per sample), error bars represent one standard deviation from the mean, statistical significance ****$P < 0.0001$ ***$P < 0.001$ **$P < 0.01$ *$P < 0.05$.

B High-resolution imaging of intestinal organoids cultured in control medium or medium supplemented with the NRG1 growth factor for 1 h which strongly stimulates pAkt, reduces pLATS staining, and drives YAP to the nucleus.

C Quantification of pAKT, pLATS and YAP immunostaining from (B).

D Schematic diagram of columnar intestinal epithelial cells with and without growth factor stimulation.

Biological replicates are plotted as individual data points ($n > 40$ per sample), error bars represent one standard deviation from the mean, statistical significance was determined using a *t*-test ****$P < 0.0001$ ***$P < 0.001$ **$P < 0.01$ *$P < 0.05$.
Source data are available online for this figure.

To explore the implications of our findings beyond epithelial tissues, we considered one cell type that shares common features with epithelial cells: neurons. In particular, "postsynaptic densities" are membrane domains at which phase separation of localised membrane-associated proteins such as PSD-95 can occur to generate discrete clusters along a dendrite that influence the stability of dendritic spines (Cane *et al*, 2014; Zeng *et al*, 2016). At least one upstream Hippo pathway component, Kibra, localises to post-synaptic densities via WW-PPXY motif interactions with the post-synaptic density enriched protein Dendrin (Heitz *et al*, 2016; Tracy *et al*, 2016; Ji *et al*, 2019). Inhibiting the Kibra-Dendrin interaction blocked long-term potentiation and impaired spatial learning and memory in mice (Ji *et al*, 2019), while *kibra* knockout mice altered the morphology of dendritic spines (Blanque *et al*, 2015). Thus, the ability of upstream Hippo pathway components to induce phase separation and condensation may be a universal feature of their molecular mechanism in both neurons and epithelial cells. Finally, while this paper was under review, Wang *et al* (2022) reported similar findings.

Materials and Methods

Drosophila genetics

Expression of UAS-driven transgenes in ovarian follicle cells was performed with the *TJ.Gal4* driver. Fly crosses were kept at 25°C. Ovaries were dissected and immunostained from adult females 3–4 days after eclosion. For starvation experiments, females of the desired genotype were fed on wetted yeast for 2 days (with males for company) before being transferred to vials containing cotton balls soaked in a 20% sucrose solution for 24 h.

For the induction of mutant clones, adult females carrying the heat shock inducible FLP recombinase enzyme (hs.flp) and homozygous FLP recombinase target (FRT) sites were heat shocked in a 37°C water bath for 1 h to induce sister chromatid recombination during mitosis, allowing for the generation of homozygous mutant daughter cell lacking GFP and a homozygous GFP^+ "twin spot" daughter cell. The mutant clone and twin were allowed to proliferate for 2–3 days prior to dissection of adult females and fixation of ovaries.

Drosophila ovary treatment with 1,6-hexanediol or sorbitol

For treatment with 1,6-hexanediol, ovaries were first dissected into PBS and subsequently transferred into 10%(w/v) 1,6-hexanediol (Sigma) prepared in PBS (or PBS alone for control) for 5 min prior to fixing. For *in vivo* molecular crowding experiments, ovaries were dissected into room temperature (RT) Schneider's media, then incubated in 1 M D-Sorbitol (Sigma) prepared in Schneider's media (or Schneider's alone for control) for 1 h before fixing.

Drosophila immunofluorescence

Ovaries were dissected in PBS, fixed for 20 min in 4% paraformaldehyde (PFA) in PBS, washed for 45 min in PBS/0.1% Triton X-100 (PBST) and blocked for 30 min in 5% normal goat serum (NGS)/PBST. Primary antibodies were diluted in NGS/PBST, and samples were incubated overnight at 4°C.

Primary antibodies used were FITC-conjugated anti-GFP (1:400; Abcam), rat anti-Crb (1:300, a gift from U.Tepass), anti-Ex (gift from R. Fehon), rabbit anti-Kib (1:200, Genevet *et al*, 2010), rabbit anti-aPKCζ (C20) (1:250 Santa Cruz), anti-pWts (gift from K. Irvine), anti-Rab5 (DSHB), anti-Rab7 (DSHB), anti-Rab11 (DSHB), anti-alpha-spec (DSHB). Secondary antibodies (goat Alexa Fluor 488, 546, 647; Invitrogen) were used at 1:500 for 2 h at RT, and samples were mounted in Vectashield (Vector Laboratories).

Image acquisition of fixed *Drosophila* egg chambers

Confocal images were taken with a Leica SP5 or Zeiss LSM780 confocal microscope using 40× oil immersion objectives and processed with Adobe Photoshop and Fiji. Optical cross-sections through the middle of the egg chamber or through the apical domain are shown in all figures.

Live imaging of *Drosophila* egg chambers

Drosophila ovaries from starved females were dissected into Schneider's media and egg chambers manually removed from the muscular sheath. Egg chambers of the correct stage were transferred to a 29 mm imaging dish (Cellvis) in 200 µl fresh Schneider's media (without supplementation to reproduce starvation conditions). Live confocal imaging was performed with a Leica SP5, with frames collected every 10 s.

FRAP in *Drosophila* follicular epithelium

FRAP experiments were performed on HpoKD-Venus puncta formed under starvation conditions, using the Leica SP5 FRAP wizard application. Individual puncta were selected by a circular region (~2 µm) and subject to three bleaching iterations using the 488-argon laser

© 2023 The Authors

set to 100% and the Zoom In function of the FRAP wizard. The 514 laser at 20% laser power was used to acquire six pre-bleach frames (1 s/frame) and 150 post-bleach frames (50 at 1 s/frame, 50 at 3 s/frame, 50 at 10 s/frame). Raw fluorescent intensity values were measured using the Leica Application Suite X (LAS X) software, and data were subject to the following normalisation: (i) background intensity was subtracted from bleached ROI. Background was determined by placing an identically sized ROI in the nucleus. (ii) The background corrected bleached ROI was normalised to the pre-bleach frames. (iii) Two background corrected, non-bleached ROI's were selected to correct for acquisition bleaching.

Drosophila genotypes

All *Drosophila* strains are described in Flybase (www.flybase.org) and are available from the Bloomington *Drosophila* Stock Centre (BDSC) or upon request. The *Drosophila* genotypes used are listed in Table 1.

Mammalian cell culture

Human Caco-2 adenocarcinoma colon cells were grown in MEM (Gibco), supplemented with MEM non-essential amino acids (Gibco), 1 mM sodium pyruvate (Gibco), 10% fetal bovine serum (FBS, Sigma) and 100 units/ml penicillin/streptomycin (Gibco). HEK293T cells were grown in DMEM (Gibco), supplemented with 2 mM L-glutamine (Gibco), 10% FBS and 100 units/ml penicillin/streptomycin. Cells were maintained in a 37°C incubator at 5% atmospheric CO_2. Cells were subject to mycoplasma testing.

HEK293T and Caco-2 cells were grown on glass coverslips and transfected using Lipofectamine 3000 Reagent according to manufacturer's instructions. Cells were fixed 24 and 48 h post-transfection, respectively. The following plasmids were used; Origene: hWWC1(Kibra)-myc-DDK (RC228642), Addgene: pEGFP-C3-LATS1 (19053), VectorBuilder: EGFP-hWWC1 FL (aa 1–1119), EGFP-hWWC1ΔC (aa 1–789), EGFP-hWWC1ΔM + C (aa 1–429 + 657–789), hSav1-EGFP (aa 1–383).

The following culture and transfection conditions were used for hSav1-YFP and mCherry-MST2 experiments. U-2 OS cells (gift from Lingfeng Chen) were cultured in DMEM supplemented with 10% FBS (Gibco), 2 mM L-glutamine (Corning) and 100 units/ml penicillin/streptomycin (Corning) at 37°C and 5% CO_2. Caco-2 cells were cultured in EMEM (ATCC-30-2003) supplemented with 20% FBS (Gibco) at 37°C and 5% CO_2. For confocal imaging, cells were cultured on 8-well LabTek chambered coverglass dishes (Thermo Scientific) and transfected with 100 ng hSav1-YFP (Addgene) and/or 100 ng mCherry-MST2 (Addgene) with Lipofectamine 3000 Reagent (Thermo Fisher, U-2 OS) or TransfeXTM (ATCC, Caco-2) according to the manufacturer's instructions. Cells were imaged 18 h after transfection on a Zeiss LSM 880 confocal microscope at 37°C with Plan-Apochromat 63×/1.40 Oil objective.

Cell culture immunofluorescence

For 2D culture, Caco-2 cells were grown on glass coverslips for 72 h prior to fixing. Cells were washed twice with PBS (containing $CaCl_2$ and $MgCl_2$) and fixed in 4% PFA in PBS for 20 min. Cells were permeabilised with 0.2% Triton X-100 for 10 min. Thoroughly washed cells were blocked with 5% NGS in PBS (blocking buffer) for 1 h at RT. Primary and secondary antibodies were diluted in blocking buffer. Coverslips were incubated with primary antibody overnight at 4°C. Following washes with PBS, Alexa Fluor secondary antibody was applied for 2 h at RT. Coverslips were mounted using Prolong Diamond (Invitrogen).

To grow Caco-2 cells as 3D spheroids, coverslips were placed in a 24-well plate and coated with undiluted Matrigel (Corning). Cells were plated in 2% Matrigel in complete media at a density of 1×10^4 cells/well. Media was changed every second day. Spheroids were fixed on day 5 of culture and processed for immunofluorescence as described for organoids.

Primary antibodies used: rabbit anti-LATS1 (3477, Cell Signaling), rabbit anti-pLATS1 (9157, Cell Signaling) and mouse anti-YAP (sc-101199, SCBT).

Table 1. Summary of *Drosophila* genotypes, corresponding to figure number.

Fig 1	A, B	TJ.Gal4/HpoKD VC; +/HpoKD VN
	D	+/TJ.Gal4, HpoKD VC; +/HpoKD VN<br>UAS.p60/TJ.Gal4, HpoKD VC; +/HpoKD VN<br>+/TJ.Gal4, HpoKD VC; UAS.myrAkt/HpoKD VN
Fig 2	A–E	TJ.Gal4/HpoKD VC; +/HpoKD VN
	G, H	TJ.Gal4/HpoKD VC; +/HpoKD VN
Fig 3	A, C	w; Hpo-YFP; +
Fig 4	A	w; Ex-YFP (R.Fehon)
	B	+/TJ.Gal4, HpoKD VC; +/HpoKD VN
	C, F	TJ.Gal4/UAS.Ex; +
	D, E	UAS.Ex/TJ.Gal4, HpoKD VC; +/HpoKD VN
	G	UAS.Kib/TJ.Gal4, HpoKD VC; +/HpoKD VN
	H	TJ.Gal4/UAS.KibGFP; +
Fig EV1	A	Crb-GFP
	B	+; Kibra-GFP
	C	+; GFP-Wts/TM6b (K.Irvine)
	D, J	w; Hpo-YFP; + (R.Fehon)
	E, K	TJ.Gal4/HpoKD VC; +/HpoKD VN
	F, G	w; Hpo-YFP; +
	H, I	TJ.Gal4/HpoKD VC; +/Sav VN8
Fig EV2	B	+/TJ.Gal4, HpoKD VC; +/HpoKD VN<br>+/TJ.Gal4, HpoKD VC; UAS.myrAkt/HpoKD VN<br>+/TJ.Gal4, HpoKD VC; UAS.Rheb/HpoKD VN
	E	+/TJ.Gal4, HpoKD VC; UAS.Atg8-mCherry/HpoKD VN
Fig EV3	A, B	+/TJ.Gal4, HpoKD VC; +/HpoKD VN<br>UAS.Ex/TJ.Gal4, HpoKD VC; +/HpoKD VN
	C	TJ.Gal4/HpoKD VC; +/HpoKD VN
	D, E	w; Hpo-YFP; +
Fig EV4	A	+/TJ.Gal4, HpoKD VC; +/HpoKD VN<br>UAS.α-spec RNAi (VDRC 25387)/TJ.Gal4, HpoKD VC; +/HpoKD VN
	C	βH-spec-YFP
	D, E	w1118<br>hsflp; FRT80B GFP / FRT80B α-spec
	F	UAS.Kib RNAi (VDRC 106507)/TJ.Gal4, HpoKD VC; +/HpoKD VN

 © 2023 The Authors

Organoid culture

Crypts were isolated from the small intestine of C57Bl6 mice. Once removed from the mouse, the intestinal segment was cleaned thoroughly in cold PBS, opened longitudinally and the villi mechanically removed. The cleaned tissue was cut into small pieces, rinsed multiple times in ice cold PBS before being resuspended in 25 ml Gentle Cell Dissociation Reagent (Stemcell Technologies) at RT for 15 min with rocking. Tissue segments were settled by gravity, the supernatant discarded and the tissue resuspended in ice cold PBS containing 0.1% BSA, pipetting up and down three times. The tissue segments were allowed to settle and the supernatant was removed and passed through a 70 μm filter. The filtrate was centrifuged and the pellet resuspended in a final volume of RT complete IntestiCult Organoid Growth Medium (Stemcell Technologies) and Matrigel Growth Factor Reduced Basement Membrane Matrix (Corning) added at equal ratios. The crypt suspension was pipetted onto glass coverslips in a 24-well plate (50 μl per well) and the Matrigel allowed to solidify for 10 min at 37°C. RT complete IntestiCult Medium was then added to each well (750 μl per well). The medium was changed every 2 days, and the organoids were fixed and processed on day 4–5 of culture. For NRG1-treated organoids, recombinant human NRG1 (100 ng/ml, R&D Systems) was added to complete IntestiCult Medium from day 0 of culture (for analysis of organoid growth) or 1 h before fixing (for acute stimulation).

Organoid immunofluorescence

Organoids were washed once in PBS and fixed with 4% PFA in PBS for 30 min. Fixative was added directly to the Matrigel dome-containing organoids, resulting in dissolution of the matrix and subsequent fixation of the organoids to the coverslip. Organoids were permeabilised with 0.5% Triton X-100/PBS for 20 min and blocked with 5% NGS in PBS/0.2% Triton X-100/0.05% Tween-20 (blocking buffer) for 30 min. Primary antibodies were diluted in blocking buffer and incubated with organoids overnight at 4°C. Secondary antibody containing Phalloidin-555 was incubated for 2 h at RT, followed by a counterstain with DAPI. Coverslips were mounted in Aqua/Poly-Mount (Polysciences, Inc.), and organoids were imaged with a Zeiss LSM780 confocal microscope, 40× oil immersion objective.

Primary antibodies were identical to those described for cell line immunofluorescence with the addition of rabbit anti-pAKT (4060, Cell Signaling).

Expression and purification of MST2:SAV1 complexes

DNA sequences corresponding to human MST2 (residues 1–484) and MBP-tagged λ-phosphatase were cloned into separate opening readings frames in pRSF-Duet (Novagen). Nucleotides corresponding to human SAV1-FL (residues 1–383) or a truncated variant, SAV1ΔN, (residues 196–383) were cloned into a modified pGEX-2TK (Sigma-Aldrich) encoding an N-terminal His8-StrepTagII-mYFP tag.

T7 Express cells (New England Biolabs) were co-transformed with both plasmids. Cells were grown in Terrific Broth to an OD600 of 2.0, protein expression induced by 0.25 mM IPTG and cells were additionally grown overnight at 16°C. Cells were lysed in 50 mM Tris pH 8.5, 400 mM NaCl and 10%(v/v) Glycerol. MST2:SAV1 complexes were purified by a combination of immobilised metal affinity chromatograph (IMAC; BioRad), streptactin affinity chromatography (Cytiva) and size-exclusion chromatography (Superose 200; GE Healthcare). Complex was concentrated to approximately 10 μM in 10 mM Tris pH 8.5, 400 mM NaCl, 5% Glycerol and 5 mM βME and used immediately.

In vitro droplet-formation assay

MST2:SAV1 complexes were diluted in 10 mM Tris, pH 8.5, 400 mM NaCl and 5 mM βME to the concentrations indicated in the presence or absence of 5% (w/v) 1,6-hexanediol, incubated for 2 h and 7.5 μL loaded into a chamber made from clean glass slide with a 120-μm double-sided sticker (Grace Biolabs). Two micrometer Z sections of each well were taken on a Nikon Eclipse Ti2 using a Hamamatsu digital camera C13440 with a 60× air-immersion lens. ImageJ was used to compile the Z-stacks and calculate the total area corresponding to the droplets. Data were graphed and analysed in Prism (GraphPad, La Jolla, California).

Human cancer biopsy data

Immunostaining of colorectal cancer epithelial cells was mined from the Human Protein Atlas database (proteinatlas.org).

Data availability

This study includes no data deposited in external repositories.

Expanded View for this article is available online.

Acknowledgements
The authors thank Kevin Slep for helpful advice on design of the Kibra IDR mutants. This work was funded by the Australian National University, an Australian Research Council Future Fellowship FT200100242 and by European Molecular Biology Laboratory Australia. Open access publishing facilitated by Australian National University, as part of the Wiley - Australian National University agreement via the Council of Australian University Librarians.

Author contributions
Teresa T Bonello: Data curation; formal analysis; investigation; methodology; writing – review and editing. **Danfeng Cai:** Investigation. **Georgina C Fletcher:** Investigation. **Kyler Wiengartner:** Investigation. **Victoria Pengilly:** Investigation. **Kimberly S Lange:** Investigation. **Zhe Liu:** Investigation. **Jennifer Lippincott-Schwartz:** Supervision; funding acquisition; project administration. **Jennifer M Kavran:** Supervision; funding acquisition; methodology; project administration. **Barry J Thompson:** Conceptualization; supervision; funding acquisition; writing – original draft; project administration; writing – review and editing.

Disclosure and competing interests statement
The authors declare that they have no conflict of interest.

References

Aguilar-Aragon M, Fletcher G, Thompson BJ (2020) The cytoskeletal motor proteins dynein and MyoV direct apical transport of crumbs. *Dev Biol* 459: 126–137

© 2023 The Authors

Alegot H, Markosian C, Rauskolb C, Yang J, Kirichenko E, Wang YC, Irvine KD (2019) Recruitment of Jub by alpha-catenin promotes Yki activity and *Drosophila* wing growth. *J Cell Sci* 132: jcs222018

Arico S, Petiot A, Bauvy C, Dubbelhuis PF, Meijer AJ, Codogno P, Ogier-Denis E (2001) The tumor suppressor PTEN positively regulates macroautophagy by inhibiting the phosphatidylinositol 3-kinase/protein kinase B pathway. *J Biol Chem* 276: 35243 – 35246

Banani SF, Lee HO, Hyman AA, Rosen MK (2017) Biomolecular condensates: organizers of cellular biochemistry. *Nat Rev Mol Cell Biol* 18: 285 – 298

Banjade S, Rosen MK (2014) Phase transitions of multivalent proteins can promote clustering of membrane receptors. *Elife* 3: e04123

Baumgartner R, Poernbacher I, Buser N, Hafen E, Stocker H (2010) The WW domain protein Kibra acts upstream of hippo in *Drosophila*. *Dev Cell* 18: 309 – 316

Beutel O, Maraspini R, Pombo-Garcia K, Martin-Lemaitre C, Honigmann A (2019) Phase separation of zonula Occludens proteins drives formation of tight junctions. *Cell* 179: 923 – 936

Bienz M (2020) Head-to-tail polymerization in the assembly of biomolecular condensates. *Cell* 182: 799 – 811

Blankenship JT, Fuller MT, Zallen JA (2007) The *Drosophila* homolog of the Exo84 exocyst subunit promotes apical epithelial identity. *J Cell Sci* 120: 3099 – 3110

Blanque A, Repetto D, Rohlmann A, Brockhaus J, Duning K, Pavenstadt H, Wolff I, Missler M (2015) Deletion of KIBRA, protein expressed in kidney and brain, increases filopodial-like long dendritic spines in neocortical and hippocampal neurons in vivo and in vitro. *Front Neuroanat* 9: 13

Boggiano JC, Fehon RG (2012) Growth control by committee: intercellular junctions, cell polarity, and the cytoskeleton regulate hippo signaling. *Dev Cell* 22: 695 – 702

Boggiano JC, Vanderzalm PJ, Fehon RG (2011) Tao-1 phosphorylates hippo/MST kinases to regulate the hippo-Salvador-warts tumor suppressor pathway. *Dev Cell* 21: 888 – 895

Borreguero-Munoz N, Fletcher GC, Aguilar-Aragon M, Elbediwy A, Vincent-Mistiaen ZI, Thompson BJ (2019) The hippo pathway integrates PI3K-Akt signals with mechanical and polarity cues to control tissue growth. *PLoS Biol* 17: e3000509

Bosch JA, Sumabat TM, Hafezi Y, Pellock BJ, Gandhi KD, Hariharan IK (2014) The *Drosophila* F-box protein Fbxl7 binds to the protocadherin fat and regulates Dachs localization and hippo signaling. *Elife* 3: e03383

Cai D, Feliciano D, Dong P, Flores E, Gruebele M, Porat-Shliom N, Sukenik S, Liu Z, Lippincott-Schwartz J (2019) Phase separation of YAP reorganizes genome topology for long-term YAP target gene expression. *Nat Cell Biol* 21: 1578 – 1589

Cane M, Maco B, Knott G, Holtmaat A (2014) The relationship between PSD-95 clustering and spine stability in vivo. *J Neurosci* 34: 2075 – 2086

Case LB, Ditlev JA, Rosen MK (2019) Regulation of transmembrane signaling by phase separation. *Annu Rev Biophys* 48: 465 – 494

Chen CL, Gajewski KM, Hamaratoglu F, Bossuyt W, Sansores-Garcia L, Tao C, Halder G (2010) The apical-basal cell polarity determinant crumbs regulates hippo signaling in *Drosophila*. *Proc Natl Acad Sci USA* 107: 15810 – 15815

Creasy CL, Ambrose DM, Chernoff J (1996) The Ste20-like protein kinase, Mst1, dimerizes and contains an inhibitory domain. *J Biol Chem* 271: 21049 – 21053

Davis JR, Tapon N (2019) Hippo signalling during development. *Development* 146: dev167106

Deng Y, Matsui Y, Zhang Y, Lai ZC (2013) Hippo activation through homodimerization and membrane association for growth inhibition and organ size control. *Dev Biol* 375: 152 – 159

Deng H, Wang W, Yu J, Zheng Y, Qing Y, Pan D (2015) Spectrin regulates hippo signaling by modulating cortical actomyosin activity. *Elife* 4: e06567

Deng H, Yang L, Wen P, Lei H, Blount P, Pan D (2020) Spectrin couples cell shape, cortical tension, and hippo signaling in retinal epithelial morphogenesis. *J Cell Biol* 219: e201907018

Dey A, Varelas X, Guan KL (2020) Targeting the hippo pathway in cancer, fibrosis, wound healing and regenerative medicine. *Nat Rev Drug Discov* 19: 480 – 494

Fan R, Kim NG, Gumbiner BM (2013) Regulation of hippo pathway by mitogenic growth factors via phosphoinositide 3-kinase and phosphoinositide-dependent kinase-1. *Proc Natl Acad Sci USA* 110: 2569 – 2574

Fletcher GC, Lucas EP, Brain R, Tournier A, Thompson BJ (2012) Positive feedback and mutual antagonism combine to polarize crumbs in the *Drosophila* follicle cell epithelium. *Curr Biol* 22: 1116 – 1122

Fletcher GC, Elbediwy A, Khanal I, Ribeiro PS, Tapon N, Thompson BJ (2015) The Spectrin cytoskeleton regulates the hippo signalling pathway. *EMBO J* 34: 940 – 954

Fletcher GC, Diaz-de-la-Loza MD, Borreguero-Munoz N, Holder M, Aguilar-Aragon M, Thompson BJ (2018) Mechanical strain regulates the hippo pathway in *Drosophila*. *Development* 145: dev159467

Gammons M, Bienz M (2018) Multiprotein complexes governing Wnt signal transduction. *Curr Opin Cell Biol* 51: 42 – 49

Genevet A, Wehr MC, Brain R, Thompson BJ, Tapon N (2010) Kibra is a regulator of the Salvador/warts/hippo signaling network. *Dev Cell* 18: 300 – 308

Hamaratoglu F, Willecke M, Kango-Singh M, Nolo R, Hyun E, Tao C, Jafar-Nejad H, Halder G (2006) The tumour-suppressor genes NF2/Merlin and Expanded act through hippo signalling to regulate cell proliferation and apoptosis. *Nat Cell Biol* 8: 27 – 36

Harvey KF, Pfleger CM, Hariharan IK (2003) The *Drosophila* Mst ortholog, hippo, restricts growth and cell proliferation and promotes apoptosis. *Cell* 114: 457 – 467

Heitz FD, Farinelli M, Mohanna S, Kahn M, Duning K, Frey MC, Pavenstadt H, Mansuy IM (2016) The memory gene KIBRA is a bidirectional regulator of synaptic and structural plasticity in the adult brain. *Neurobiol Learn Mem* 135: 100 – 114

Huang J, Wu S, Barrera J, Matthews K, Pan D (2005) The hippo signaling pathway coordinately regulates cell proliferation and apoptosis by inactivating Yorkie, the *Drosophila* homolog of YAP. *Cell* 122: 421 – 434

Hyman AA, Weber CA, Julicher F (2014) Liquid-liquid phase separation in biology. *Annu Rev Cell Dev Biol* 30: 39 – 58

Irvine KD, Harvey KF (2015) Control of organ growth by patterning and hippo signaling in *Drosophila*. *Cold Spring Harb Perspect Biol* 7: a019224

Jarde T, Chan WH, Rossello FJ, Kaur Kahlon T, Theocharous M, Kurian Arackal T, Flores T, Giraud M, Richards E, Chan E *et al* (2020) Mesenchymal niche-derived Neuregulin-1 drives intestinal stem cell proliferation and regeneration of damaged epithelium. *Cell Stem Cell* 27: 646 – 662

Ji Z, Li H, Yang Z, Huang X, Ke X, Ma S, Lin Z, Lu Y, Zhang M (2019) Kibra modulates learning and memory via binding to Dendrin. *Cell Rep* 26: 2064 – 2077

Jin Y, Dong L, Lu Y, Wu W, Hao Q, Zhou Z, Jiang J, Zhao Y, Zhang L (2012) Dimerization and cytoplasmic localization regulate hippo kinase signaling activity in organ size control. *J Biol Chem* 287: 5784 – 5796

Justice RW, Zilian O, Woods DF, Noll M, Bryant PJ (1995) The *Drosophila* tumor suppressor gene warts encodes a homolog of human myotonic dystrophy kinase and is required for the control of cell shape and proliferation. *Genes Dev* 9: 534 – 546

Kanai F, Marignani PA, Sarbassova D, Yagi R, Hall RA, Donowitz M, Hisaminato A, Fujiwara T, Ito Y, Cantley LC *et al* (2000) TAZ: a novel

 © 2023 The Authors

transcriptional co-activator regulated by interactions with 14-3-3 and PDZ domain proteins. *EMBO J* 19: 6778 – 6791

Kango-Singh M, Nolo R, Tao C, Verstreken P, Hiesinger PR, Bellen HJ, Halder G (2002) Shar-pei mediates cell proliferation arrest during imaginal disc growth in *Drosophila*. *Development* 129: 5719 – 5730

Karaman R, Halder G (2018) Cell junctions in hippo signaling. *Cold Spring Harb Perspect Biol* 10: a028753

Kato M, McKnight SL (2017) Cross-beta polymerization of low complexity sequence domains. *Cold Spring Harb Perspect Biol* 9: a023598

Khanal I, Elbediwy A, de la Loza D, Mdel C, Fletcher GC, Thompson BJ (2016) Shot and Patronin polarise microtubules to direct membrane traffic and biogenesis of microvilli in epithelia. *J Cell Sci* 129: 2651 – 2659

Li P, Banjade S, Cheng HC, Kim S, Chen B, Guo L, Llaguno M, Hollingsworth JV, King DS, Banani SF *et al* (2012) Phase transitions in the assembly of multivalent signalling proteins. *Nature* 483: 336 – 340

Ling C, Zheng Y, Yin F, Yu J, Huang J, Hong Y, Wu S, Pan D (2010) The apical transmembrane protein crumbs functions as a tumor suppressor that regulates hippo signaling by binding to expanded. *Proc Natl Acad Sci USA* 107: 10532 – 10537

Mao Y, Rauskolb C, Cho E, Hu WL, Hayter H, Minihan G, Katz FN, Irvine KD (2006) Dachs: an unconventional myosin that functions downstream of fat to regulate growth, affinity and gene expression in *Drosophila*. *Development* 133: 2539 – 2551

Mao Y, Tournier AL, Bates PA, Gale JE, Tapon N, Thompson BJ (2011) Planar polarization of the atypical myosin Dachs orients cell divisions in *Drosophila*. *Genes Dev* 25: 131 – 136

Mauvezin C, Ayala C, Braden CR, Kim J, Neufeld TP (2014) Assays to monitor autophagy in *Drosophila*. *Methods* 68: 134 – 139

McCartney BM, Kulikauskas RM, LaJeunesse DR, Fehon RG (2000) The neurofibromatosis-2 homologue, Merlin, and the tumor suppressor expanded function together in *Drosophila* to regulate cell proliferation and differentiation. *Development* 127: 1315 – 1324

McSwiggen DT, Mir M, Darzacq X, Tjian R (2019) Evaluating phase separation in live cells: diagnosis, caveats, and functional consequences. *Genes Dev* 33: 1619 – 1634

Medina E, Williams J, Klipfell E, Zarnescu D, Thomas G, Le Bivic A (2002) Crumbs interacts with moesin and beta(heavy)-spectrin in the apical membrane skeleton of *Drosophila*. *J Cell Biol* 158: 941 – 951

Misra JR, Irvine KD (2018) The hippo signaling network and its biological functions. *Annu Rev Genet* 52: 65 – 87

Moya IM, Halder G (2019) Hippo-YAP/TAZ signalling in organ regeneration and regenerative medicine. *Nat Rev Mol Cell Biol* 20: 211 – 226

Ni L, Li S, Yu J, Min J, Brautigam CA, Tomchick DR, Pan D, Luo X (2013) Structural basis for autoactivation of human Mst2 kinase and its regulation by RASSF5. *Structure* 21: 1757 – 1768

Ni L, Zheng Y, Hara M, Pan D, Luo X (2015) Structural basis for Mob1-dependent activation of the core Mst-Lats kinase cascade in hippo signaling. *Genes Dev* 29: 1416 – 1431

Noda T, Ohsumi Y (1998) Tor, a phosphatidylinositol kinase homologue, controls autophagy in yeast. *J Biol Chem* 273: 3963 – 3966

Oh H, Irvine KD (2008) In vivo regulation of Yorkie phosphorylation and localization. *Development* 135: 1081 – 1088

Oh H, Irvine KD (2009) In vivo analysis of Yorkie phosphorylation sites. *Oncogene* 28: 1916 – 1927

Pan Y, Heemskerk I, Ibar C, Shraiman BI, Irvine KD (2016) Differential growth triggers mechanical feedback that elevates hippo signaling. *Proc Natl Acad Sci USA* 113: E6974 – E6983

Pan Y, Alegot H, Rauskolb C, Irvine KD (2018) The dynamics of hippo signaling during *Drosophila* wing development. *Development* 145: dev165712

Pantalacci S, Tapon N, Leopold P (2003) The Salvador partner hippo promotes apoptosis and cell-cycle exit in *Drosophila*. *Nat Cell Biol* 5: 921 – 927

Poon CL, Lin JI, Zhang X, Harvey KF (2011) The sterile 20-like kinase Tao-1 controls tissue growth by regulating the Salvador-warts-hippo pathway. *Dev Cell* 21: 896 – 906

Praskova M, Khoklatchev A, Ortiz-Vega S, Avruch J (2004) Regulation of the MST1 kinase by autophosphorylation, by the growth inhibitory proteins, RASSF1 and NORE1, and by Ras. *Biochem J* 381: 453 – 462

Praskova M, Xia F, Avruch J (2008) MOBKL1A/MOBKL1B phosphorylation by MST1 and MST2 inhibits cell proliferation. *Curr Biol* 18: 311 – 321

Rauskolb C, Sun S, Sun G, Pan Y, Irvine KD (2014) Cytoskeletal tension inhibits hippo signaling through an Ajuba-warts complex. *Cell* 158: 143 – 156

Ribeiro PS, Josue F, Wepf A, Wehr MC, Rinner O, Kelly G, Tapon N, Gstaiger M (2010) Combined functional genomic and proteomic approaches identify a PP2A complex as a negative regulator of hippo signaling. *Mol Cell* 39: 521 – 534

Robinson BS, Huang J, Hong Y, Moberg KH (2010) Crumbs regulates Salvador/warts/hippo signaling in *Drosophila* via the FERM-domain protein expanded. *Curr Biol* 20: 582 – 590

Rodrigues-Campos M, Thompson BJ (2014) The ubiquitin ligase FbxL7 regulates the Dachsous-fat-Dachs system in *Drosophila*. *Development* 141: 4098 – 4103

Roeth JF, Sawyer JK, Wilner DA, Peifer M (2009) Rab11 helps maintain apical crumbs and adherens junctions in the *Drosophila* embryonic ectoderm. *PLoS ONE* 4: e7634

Schaefer KN, Peifer M (2019) Wnt/Beta-catenin signaling regulation and a role for biomolecular condensates. *Dev Cell* 48: 429 – 444

Schwayer C, Shamipour S, Pranjic-Ferscha K, Schauer A, Balda M, Tada M, Matter K, Heisenberg CP (2019) Mechanosensation of tight junctions depends on ZO-1 phase separation and flow. *Cell* 179: 937 – 952

Scott RC, Schuldiner O, Neufeld TP (2004) Role and regulation of starvation-induced autophagy in the *Drosophila* fat body. *Dev Cell* 7: 167 – 178

Scott RC, Juhasz G, Neufeld TP (2007) Direct induction of autophagy by Atg1 inhibits cell growth and induces apoptotic cell death. *Curr Biol* 17: 1 – 11

Sherrard KM, Fehon RG (2015) The transmembrane protein crumbs displays complex dynamics during follicular morphogenesis and is regulated competitively by Moesin and aPKC. *Development* 142: 2226

Shin Y, Brangwynne CP (2017) Liquid phase condensation in cell physiology and disease. *Science* 357: eaaf4382

Strassburger K, Tiebe M, Pinna F, Breuhahn K, Teleman AA (2012) Insulin/IGF signaling drives cell proliferation in part via Yorkie/YAP. *Dev Biol* 367: 187 – 196

Su X, Ditlev JA, Hui E, Xing W, Banjade S, Okrut J, King DS, Taunton J, Rosen MK, Vale RD (2016) Phase separation of signaling molecules promotes T cell receptor signal transduction. *Science* 352: 595 – 599

Su T, Ludwig MZ, Xu J, Fehon RG (2017) Kibra and Merlin activate the hippo pathway spatially distinct from and independent of expanded. *Dev Cell* 40: 478 – 490

Sun S, Reddy BV, Irvine KD (2015) Localization of hippo signalling complexes and warts activation in vivo. *Nat Commun* 6: 8402

Tapon N, Harvey KF, Bell DW, Wahrer DC, Schiripo TA, Haber D, Hariharan IK (2002) Salvador promotes both cell cycle exit and apoptosis in *Drosophila* and is mutated in human cancer cell lines. *Cell* 110: 467 – 478

Thompson BJ (2020) YAP/TAZ: drivers of tumor growth, metastasis, and resistance to therapy. *BioEssays* 42: e1900162

Tracy TE, Sohn PD, Minami SS, Wang C, Min SW, Li Y, Zhou Y, Le D, Lo I, Ponnusamy R *et al* (2016) Acetylated tau obstructs KIBRA-mediated

© 2023 The Authors

signaling in synaptic plasticity and promotes Tauopathy-related memory loss. *Neuron* 90: 245–260

Tran T, Mitra J, Ha T, Kavran JM (2020) Increasing kinase domain proximity promotes MST2 autophosphorylation during hippo signaling. *J Biol Chem* 295: 16166–16179

Udan RS, Kango-Singh M, Nolo R, Tao C, Halder G (2003) Hippo promotes proliferation arrest and apoptosis in the Salvador/warts pathway. *Nat Cell Biol* 5: 914–920

van Leeuwen W, Rabouille C (2019) Cellular stress leads to the formation of membraneless stress assemblies in eukaryotic cells. *Traffic* 20: 623–638

Vrabioiu AM, Struhl G (2015) Fat/Dachsous signaling promotes *Drosophila* wing growth by regulating the conformational state of the NDR kinase warts. *Dev Cell* 35: 737–749

Wang L, Choi K, Su T, Li B, Wu X, Zhang R, Driskill JH, Li H, Lei H, Guo P *et al* (2022) Multiphase coalescence mediates hippo pathway activation. *Cell* 185: 4376–4393

Wei X, Shimizu T, Lai ZC (2007) Mob as tumor suppressor is activated by hippo kinase for growth inhibition in *Drosophila*. *EMBO J* 26: 1772–1781

Wu S, Huang J, Dong J, Pan D (2003) Hippo encodes a Ste-20 family protein kinase that restricts cell proliferation and promotes apoptosis in conjunction with Salvador and warts. *Cell* 114: 445–456

Xu T, Wang W, Zhang S, Stewart RA, Yu W (1995) Identifying tumor suppressors in genetic mosaics: the *Drosophila* lats gene encodes a putative protein kinase. *Development* 121: 1053–1063

Yin F, Yu J, Zheng Y, Chen Q, Zhang N, Pan D (2013) Spatial organization of hippo signaling at the plasma membrane mediated by the tumor suppressor Merlin/NF2. *Cell* 154: 1342–1355

Yu J, Zheng Y, Dong J, Klusza S, Deng WM, Pan D (2010) Kibra functions as a tumor suppressor protein that regulates hippo signaling in conjunction with Merlin and Expanded. *Dev Cell* 18: 288–299

Yue T, Tian A, Jiang J (2012) The cell adhesion molecule echinoid functions as a tumor suppressor and upstream regulator of the hippo signaling pathway. *Dev Cell* 22: 255–267

Zeng M, Shang Y, Araki Y, Guo T, Huganir RL, Zhang M (2016) Phase transition in postsynaptic densities underlies formation of synaptic complexes and synaptic plasticity. *Cell* 166: 1163–1175

Zhao B, Wei X, Li W, Udan RS, Yang Q, Kim J, Xie J, Ikenoue T, Yu J, Li L *et al* (2007) Inactivation of YAP oncoprotein by the hippo pathway is involved in cell contact inhibition and tissue growth control. *Genes Dev* 21: 2747–2761

Zheng Y, Pan D (2019) The hippo signaling pathway in development and disease. *Dev Cell* 50: 264–282

Zheng Y, Liu B, Wang L, Lei H, Pulgar Prieto KD, Pan D (2017) Homeostatic control of Hpo/MST kinase activity through autophosphorylation-dependent recruitment of the STRIPAK PP2A phosphatase complex. *Cell Rep* 21: 3612–3623

Zhu L, Brangwynne CP (2015) Nuclear bodies: the emerging biophysics of nucleoplasmic phases. *Curr Opin Cell Biol* 34: 23–30

License: This is an open access article under the terms of the Creative Commons Attribution License, which permits use, distribution and reproduction in any medium, provided the original work is properly cited.

 © 2023 The Authors
