# Supplementary figures and images for: Phase separation of Hippo signalling complexes (part 1 of 2)
Source: EMBO J. 2023 Feb 20;42(6):e112863. doi: 10.15252/embj.2022112863 (PMC10015380; doi:10.15252/embj.2022112863)

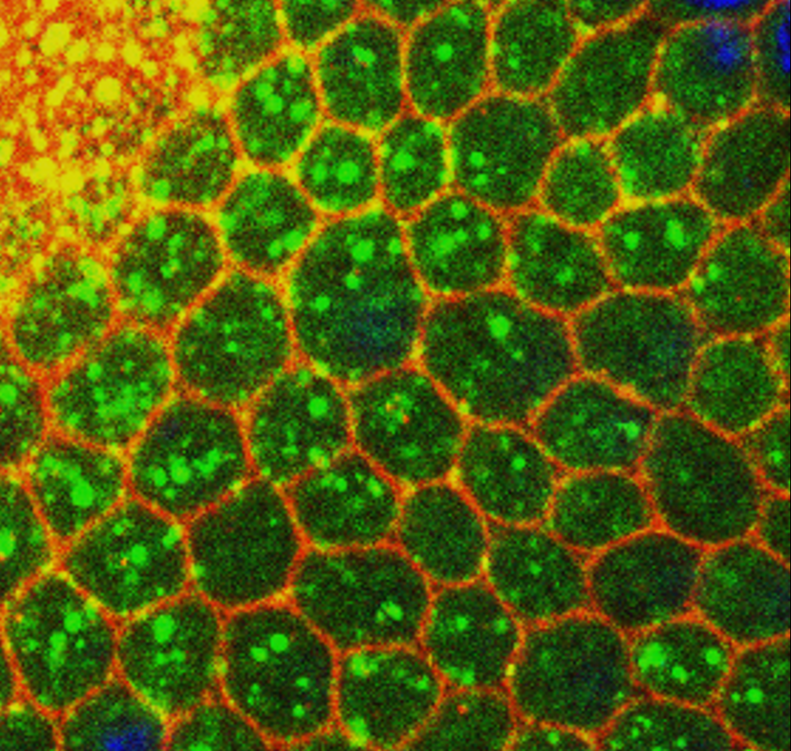

Supplement: Supplementary file 4 — Source Data for Figure 1 [file EMBJ-42-e112863-s001.zip › Fig1/Fig1B4.tif]

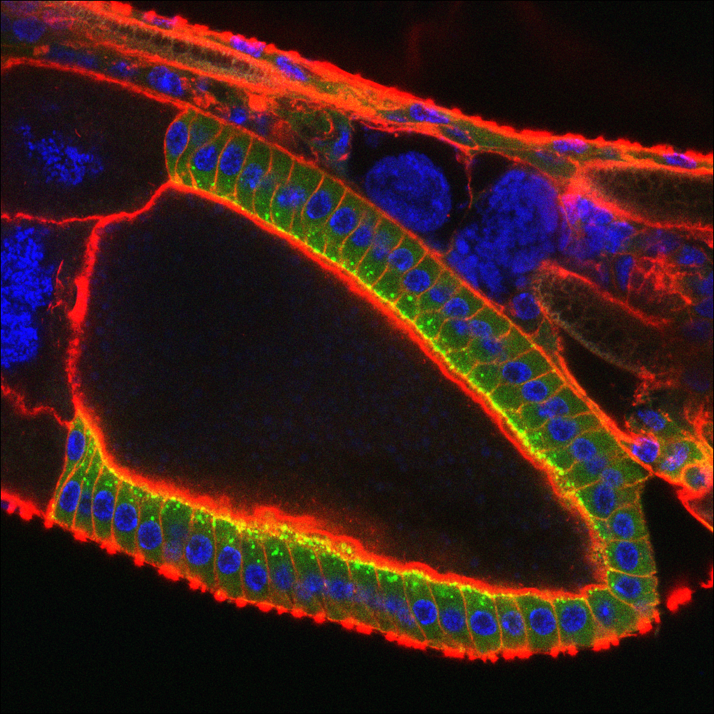

Supplement: Supplementary file 4 — Source Data for Figure 1 [file EMBJ-42-e112863-s001.zip › Fig1/Fig1B1.tif]

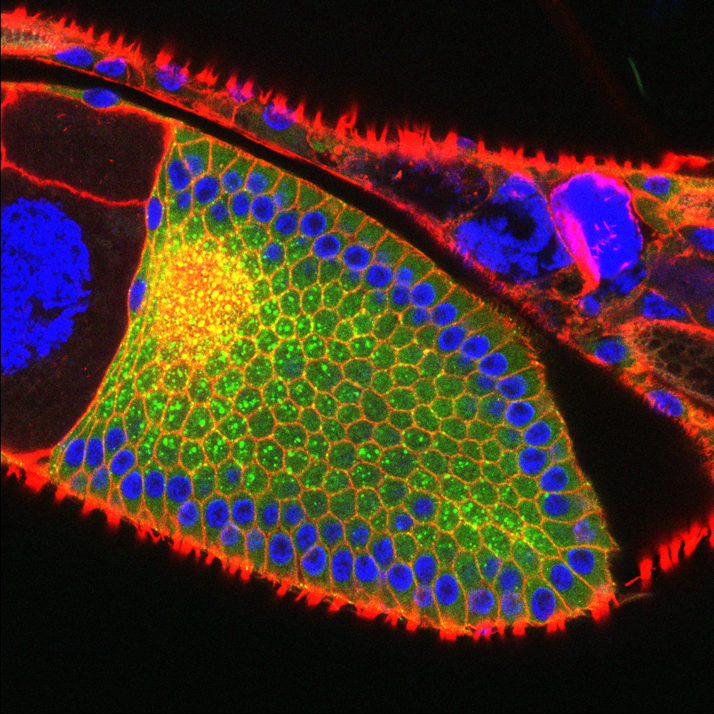

Supplement: Supplementary file 4 — Source Data for Figure 1 [file EMBJ-42-e112863-s001.zip › Fig1/Fig1B2.tif]

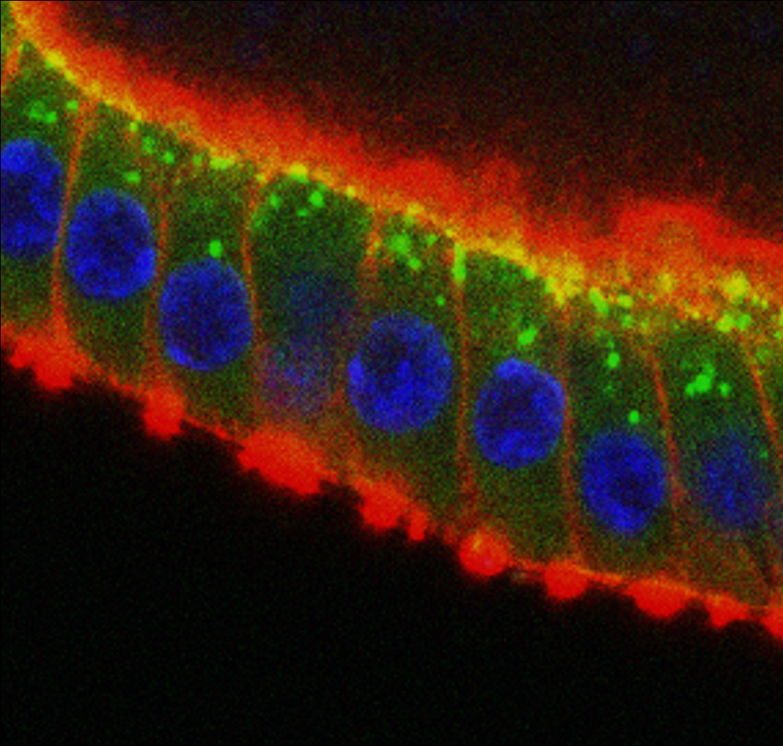

Supplement: Supplementary file 4 — Source Data for Figure 1 [file EMBJ-42-e112863-s001.zip › Fig1/Fig1B3.tif]

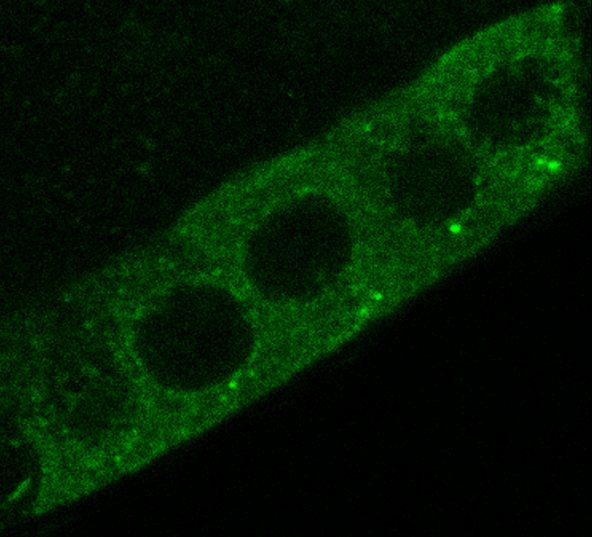

Supplement: Supplementary file 4 — Source Data for Figure 1 [file EMBJ-42-e112863-s001.zip › Fig1/Fig1D4.tif]

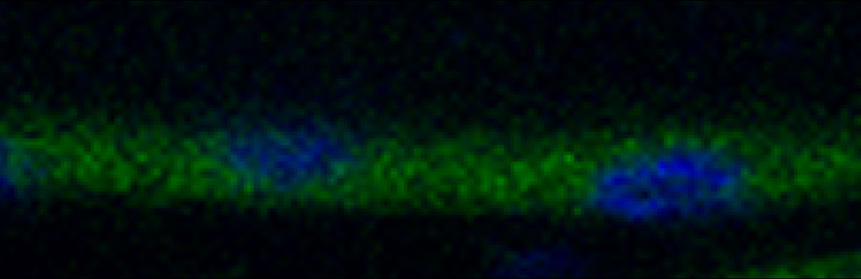

Supplement: Supplementary file 4 — Source Data for Figure 1 [file EMBJ-42-e112863-s001.zip › Fig1/Fig1D2.tif]

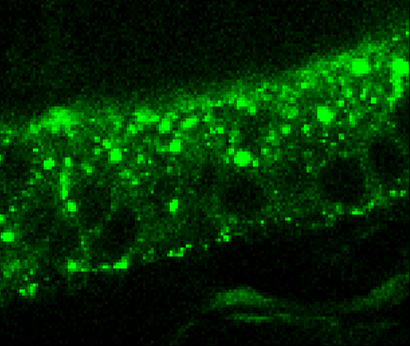

Supplement: Supplementary file 4 — Source Data for Figure 1 [file EMBJ-42-e112863-s001.zip › Fig1/Fig1D3.tif]

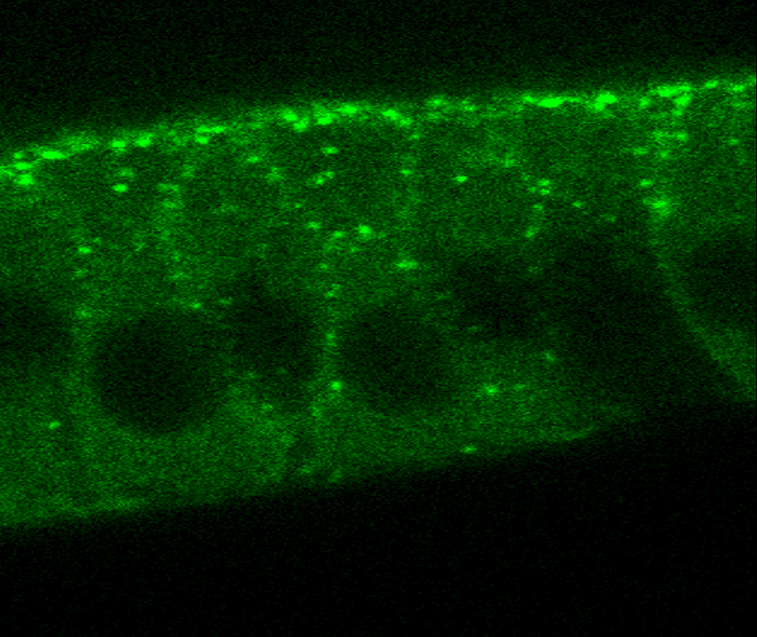

Supplement: Supplementary file 4 — Source Data for Figure 1 [file EMBJ-42-e112863-s001.zip › Fig1/Fig1D1.tif]

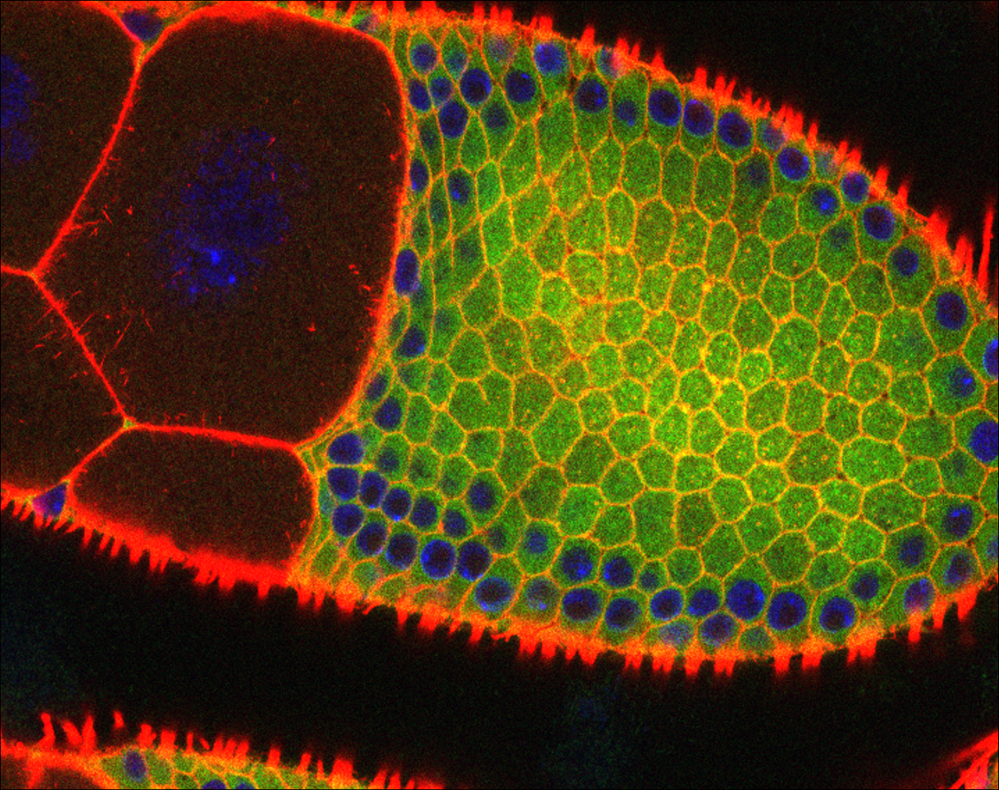

Supplement: Supplementary file 4 — Source Data for Figure 1 [file EMBJ-42-e112863-s001.zip › Fig1/Fig1A2.tif]

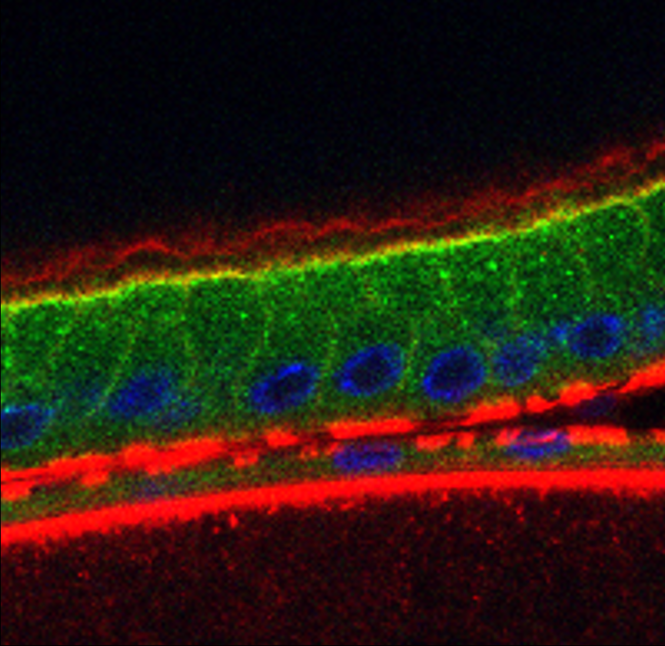

Supplement: Supplementary file 4 — Source Data for Figure 1 [file EMBJ-42-e112863-s001.zip › Fig1/Fig1A3.tif]

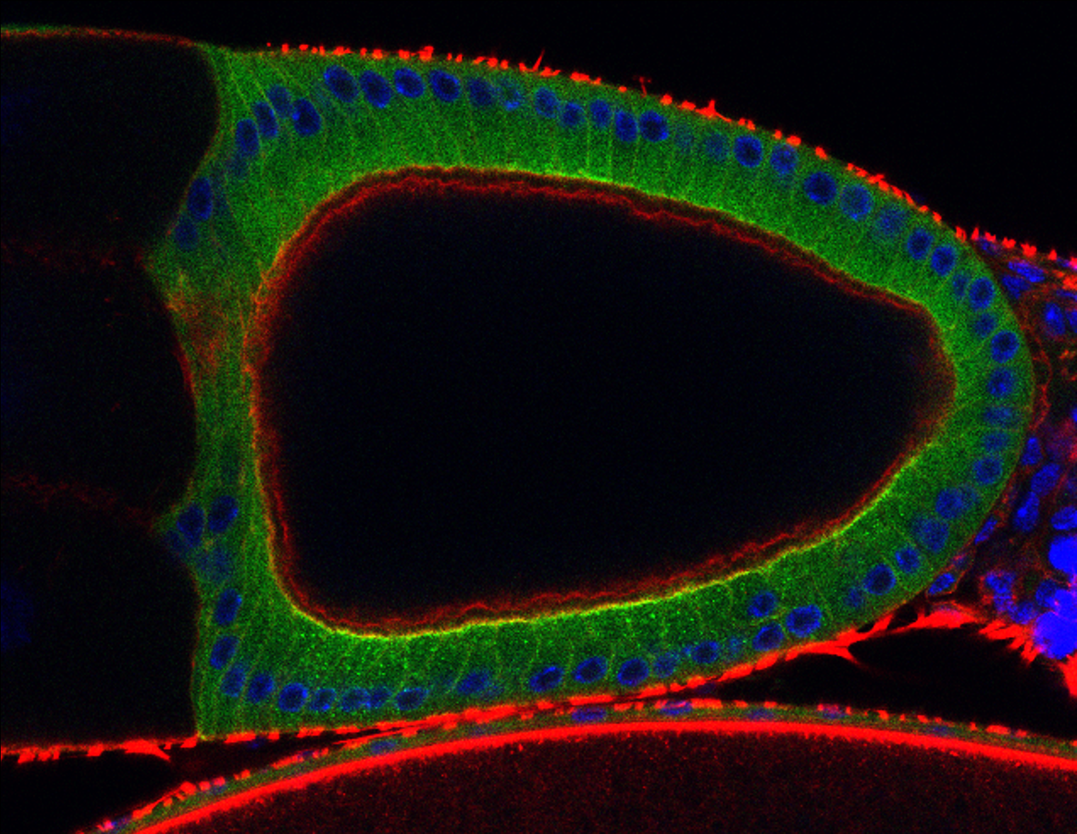

Supplement: Supplementary file 4 — Source Data for Figure 1 [file EMBJ-42-e112863-s001.zip › Fig1/Fig1A1.tif]

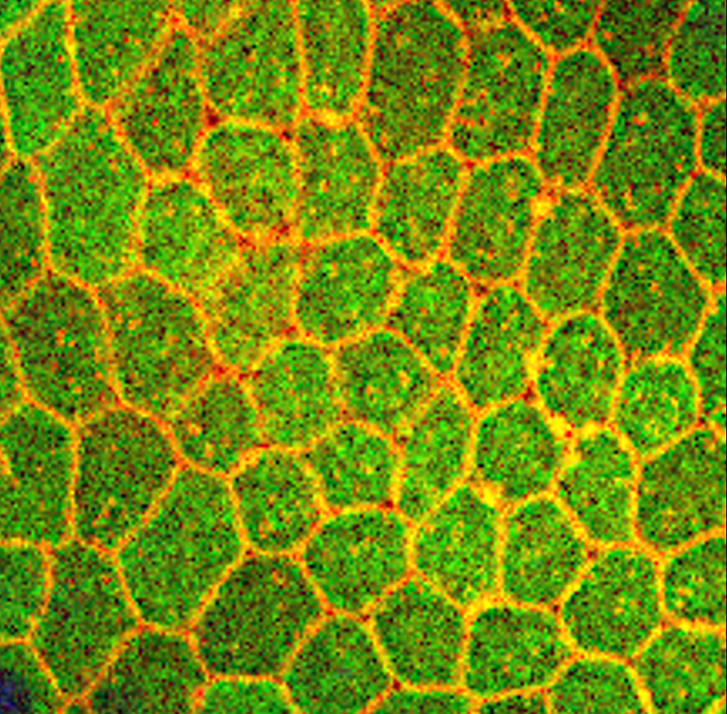

Supplement: Supplementary file 4 — Source Data for Figure 1 [file EMBJ-42-e112863-s001.zip › Fig1/Fig1A4.tif]

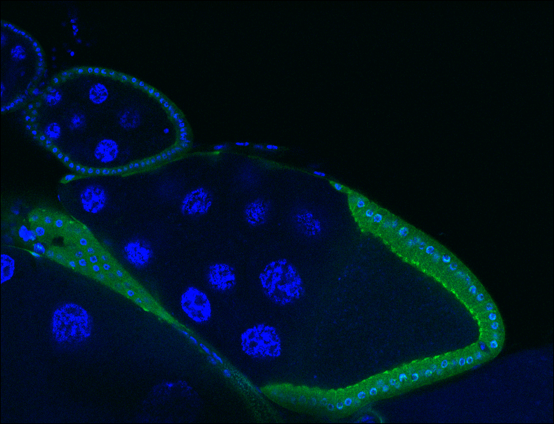

Supplement: Supplementary file 5 — Source Data for Figure 2 [file EMBJ-42-e112863-s012.zip › Fig2/Fig2E1.tif]

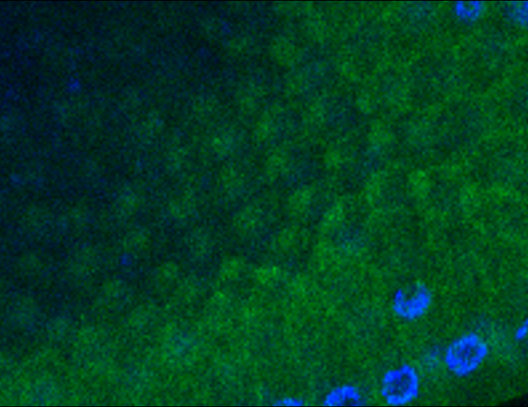

Supplement: Supplementary file 5 — Source Data for Figure 2 [file EMBJ-42-e112863-s012.zip › Fig2/Fig2E2.tif]

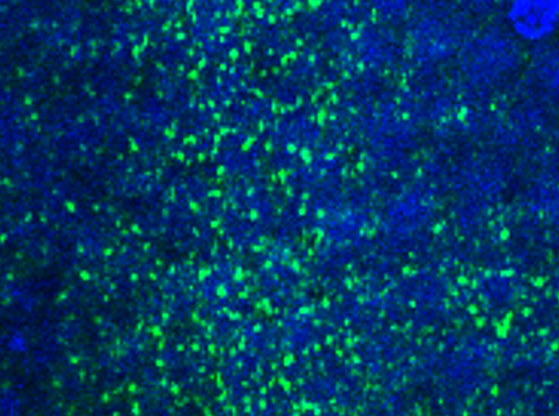

Supplement: Supplementary file 5 — Source Data for Figure 2 [file EMBJ-42-e112863-s012.zip › Fig2/Fig2D2.tif]

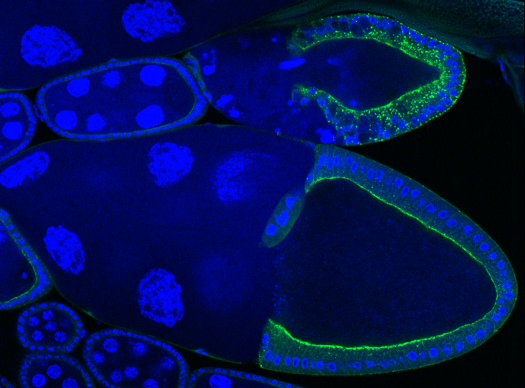

Supplement: Supplementary file 5 — Source Data for Figure 2 [file EMBJ-42-e112863-s012.zip › Fig2/Fig2D1.tif]

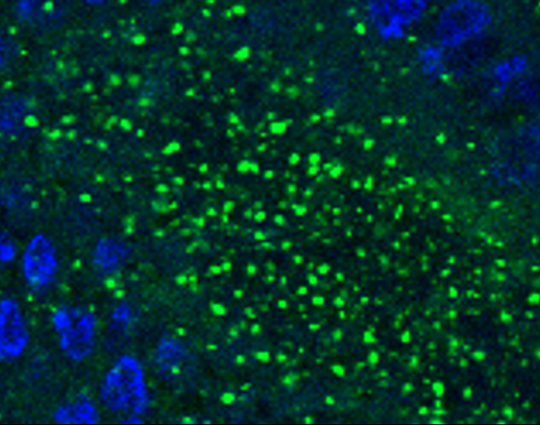

Supplement: Supplementary file 5 — Source Data for Figure 2 [file EMBJ-42-e112863-s012.zip › Fig2/Fig2C2.tif]

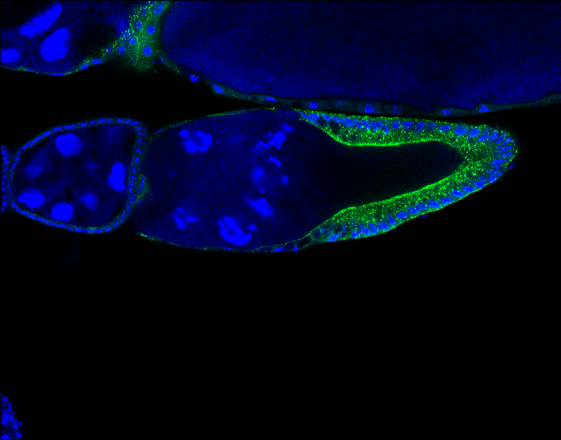

Supplement: Supplementary file 5 — Source Data for Figure 2 [file EMBJ-42-e112863-s012.zip › Fig2/Fig2C1.tif]

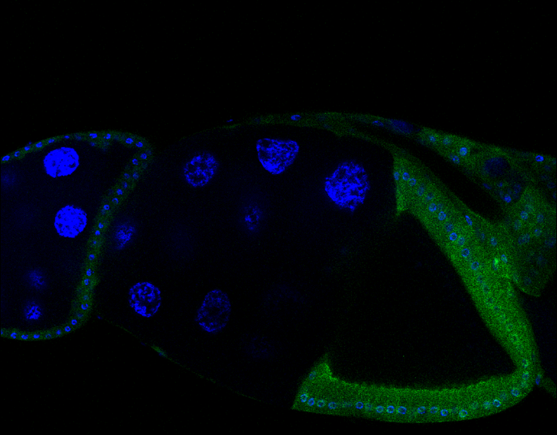

Supplement: Supplementary file 5 — Source Data for Figure 2 [file EMBJ-42-e112863-s012.zip › Fig2/Fig2B1.tif]

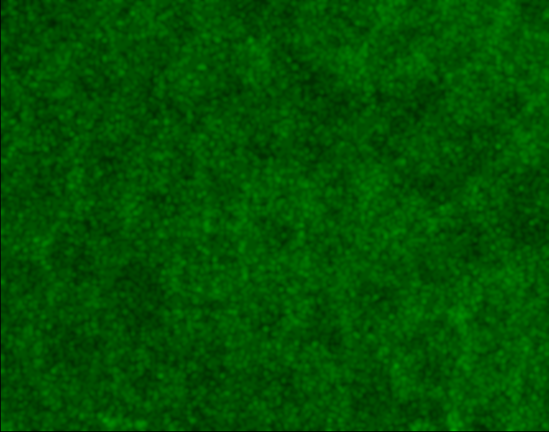

Supplement: Supplementary file 5 — Source Data for Figure 2 [file EMBJ-42-e112863-s012.zip › Fig2/Fig2B2.tif]

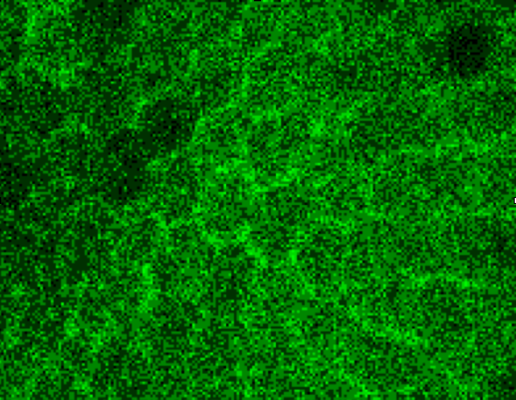

Supplement: Supplementary file 5 — Source Data for Figure 2 [file EMBJ-42-e112863-s012.zip › Fig2/Fig2A2.tif]

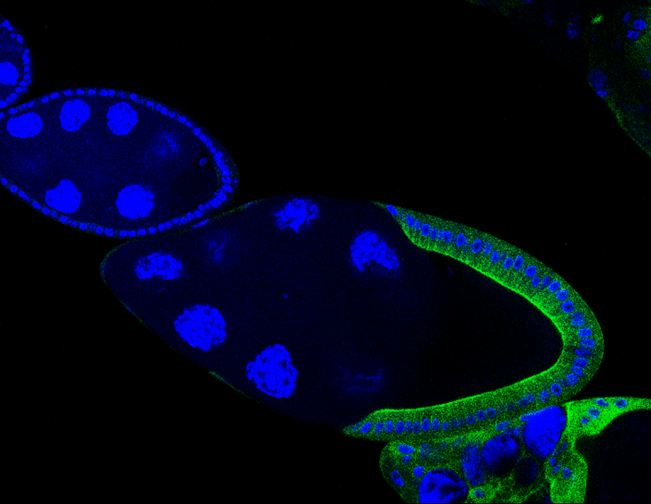

Supplement: Supplementary file 5 — Source Data for Figure 2 [file EMBJ-42-e112863-s012.zip › Fig2/Fig2A1.tif]

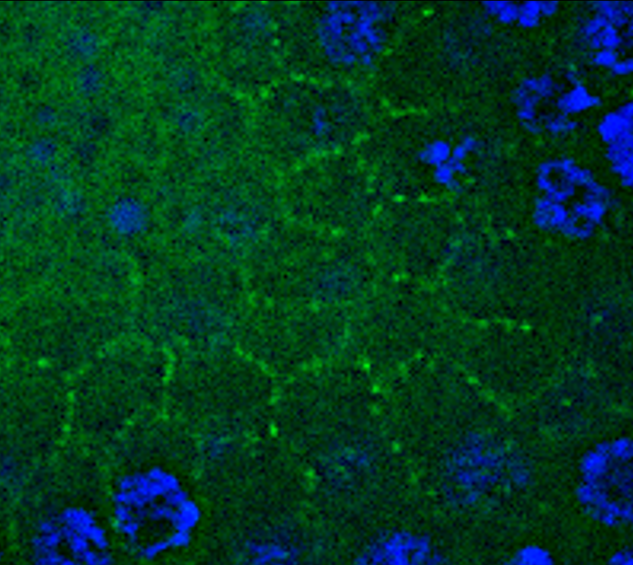

Supplement: Supplementary file 6 — Source Data for Figure 3 [file EMBJ-42-e112863-s010.zip › Fig3/Fig3C1.tif]

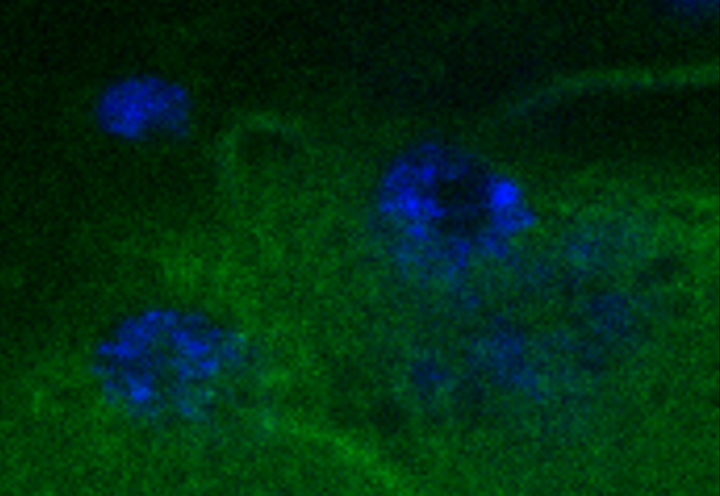

Supplement: Supplementary file 6 — Source Data for Figure 3 [file EMBJ-42-e112863-s010.zip › Fig3/Fig3C2.tif]

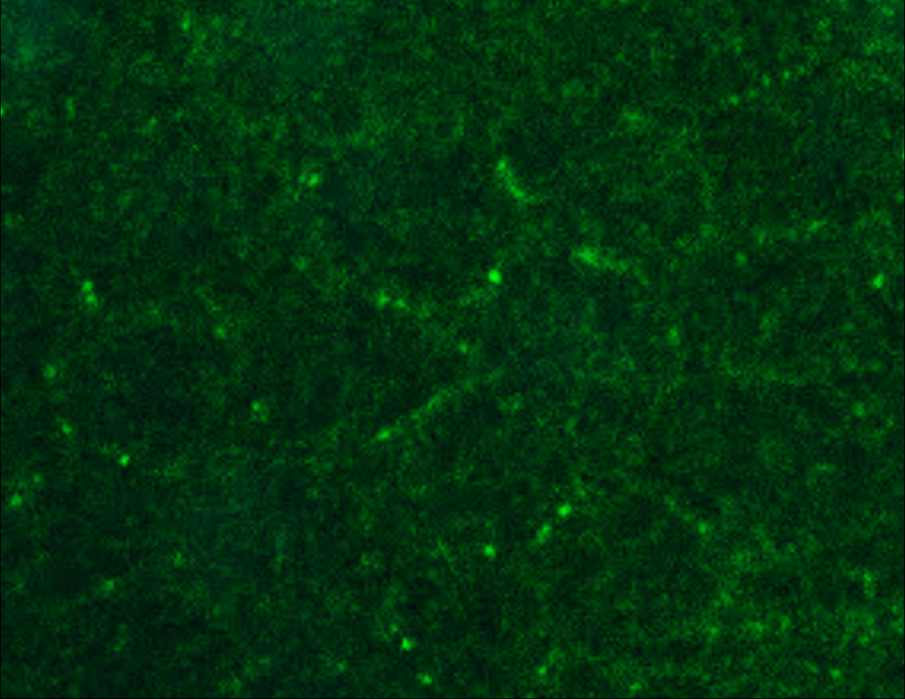

Supplement: Supplementary file 6 — Source Data for Figure 3 [file EMBJ-42-e112863-s010.zip › Fig3/Fig3A1.tif]

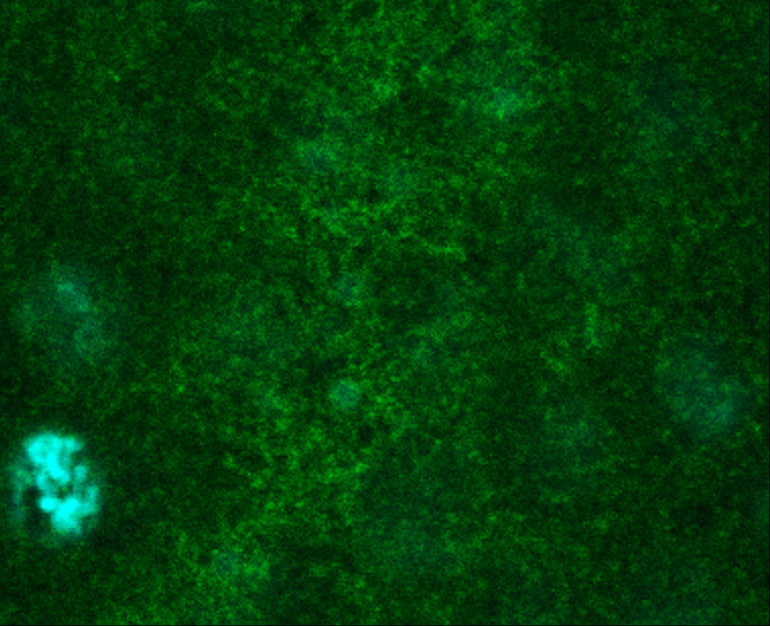

Supplement: Supplementary file 6 — Source Data for Figure 3 [file EMBJ-42-e112863-s010.zip › Fig3/Fig3A3.tif]

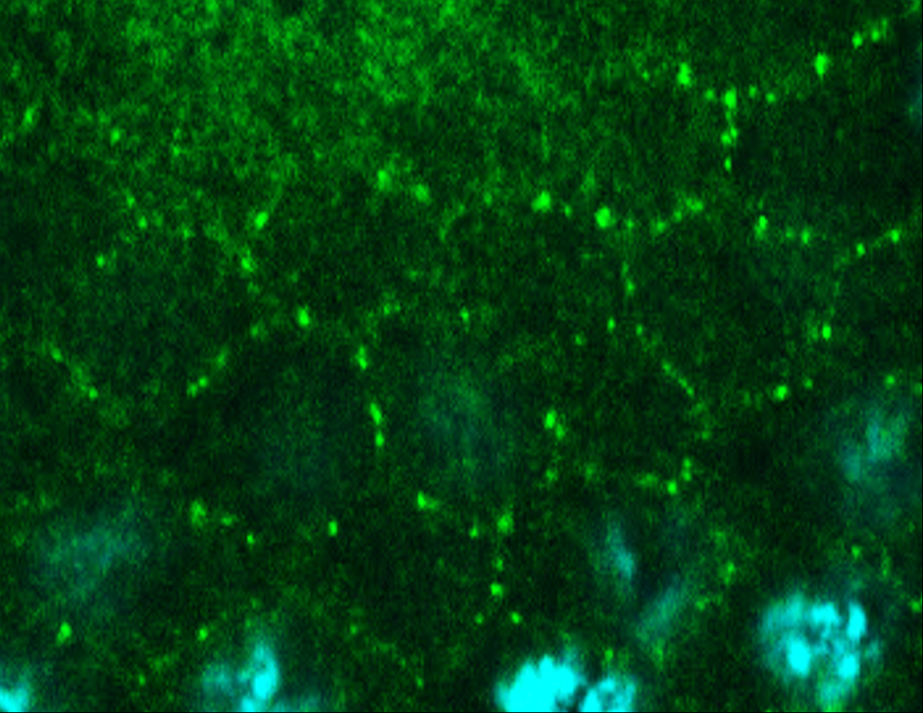

Supplement: Supplementary file 6 — Source Data for Figure 3 [file EMBJ-42-e112863-s010.zip › Fig3/Fig3A2.tif]

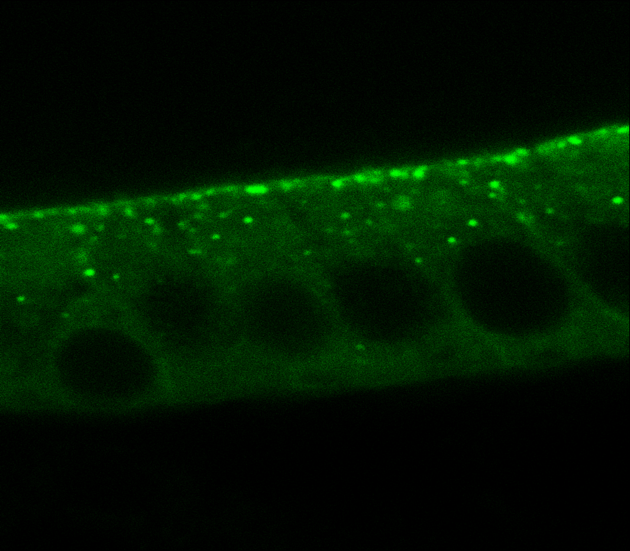

Supplement: Supplementary file 7 — Source Data for Figure 4 [file EMBJ-42-e112863-s004.zip › Fig4/Fig4D3.tif]

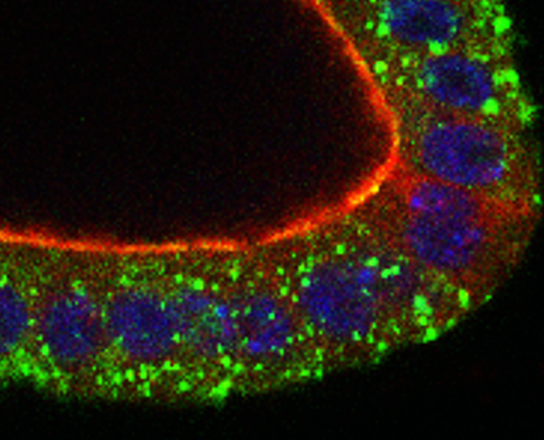

Supplement: Supplementary file 7 — Source Data for Figure 4 [file EMBJ-42-e112863-s004.zip › Fig4/Fig4H1.tif]

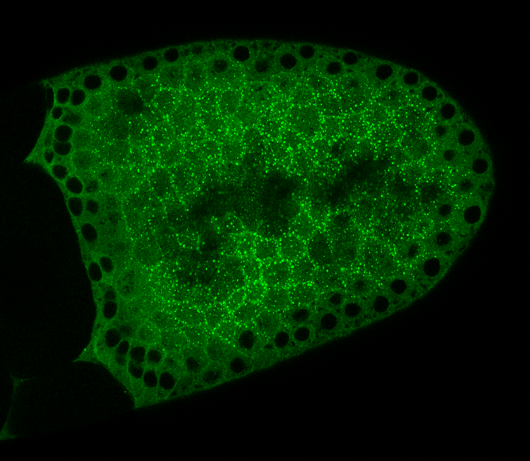

Supplement: Supplementary file 7 — Source Data for Figure 4 [file EMBJ-42-e112863-s004.zip › Fig4/Fig4D2.tif]

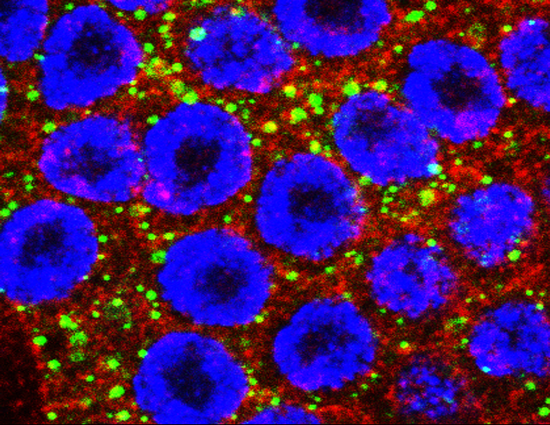

Supplement: Supplementary file 7 — Source Data for Figure 4 [file EMBJ-42-e112863-s004.zip › Fig4/Fig4H2.tif]

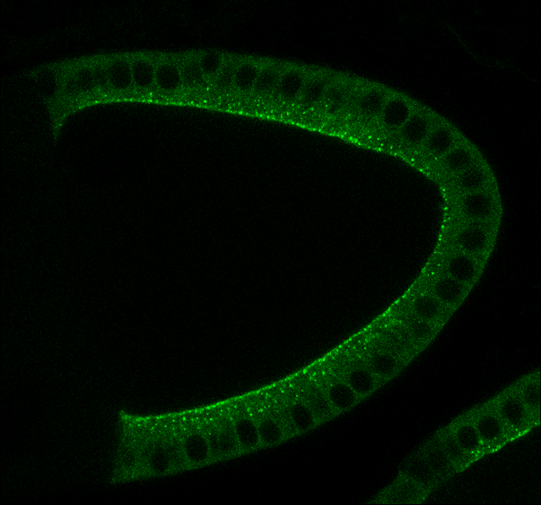

Supplement: Supplementary file 7 — Source Data for Figure 4 [file EMBJ-42-e112863-s004.zip › Fig4/Fig4D1.tif]

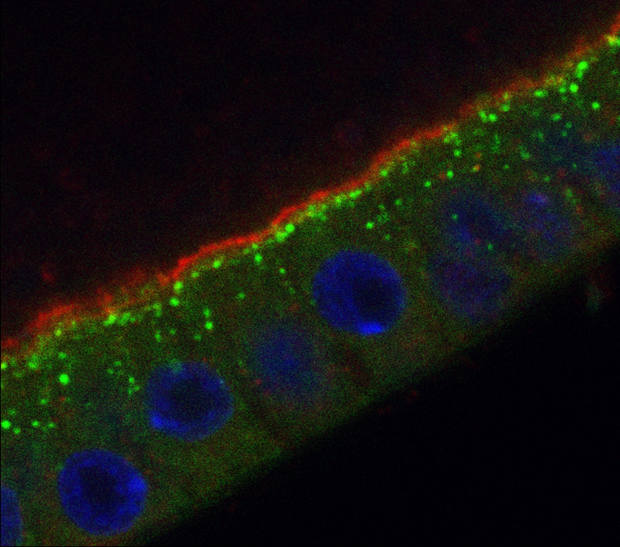

Supplement: Supplementary file 7 — Source Data for Figure 4 [file EMBJ-42-e112863-s004.zip › Fig4/Fig4E1.tif]

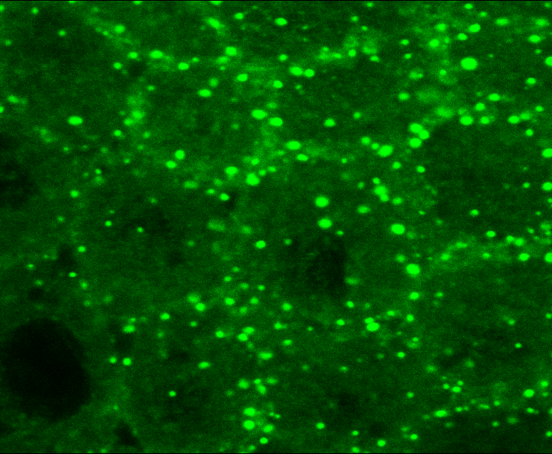

Supplement: Supplementary file 7 — Source Data for Figure 4 [file EMBJ-42-e112863-s004.zip › Fig4/Fig4D4.tif]

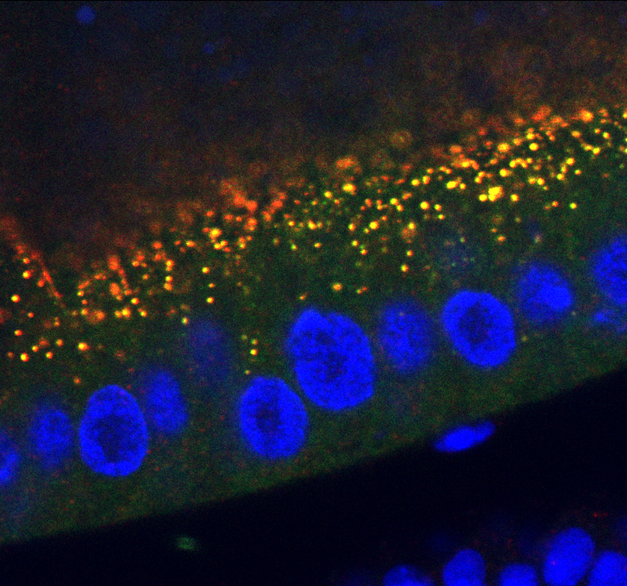

Supplement: Supplementary file 7 — Source Data for Figure 4 [file EMBJ-42-e112863-s004.zip › Fig4/Fig4E2.tif]

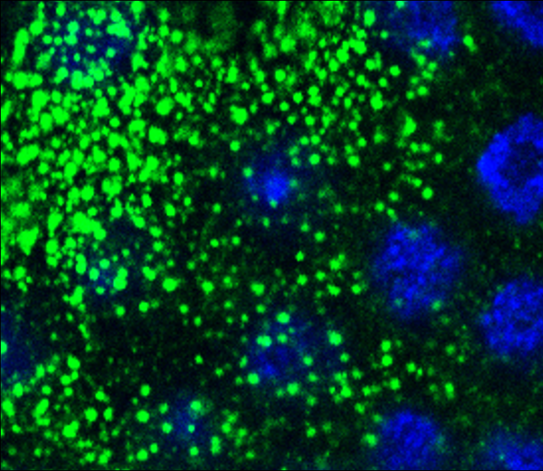

Supplement: Supplementary file 7 — Source Data for Figure 4 [file EMBJ-42-e112863-s004.zip › Fig4/Fig4C4.tif]

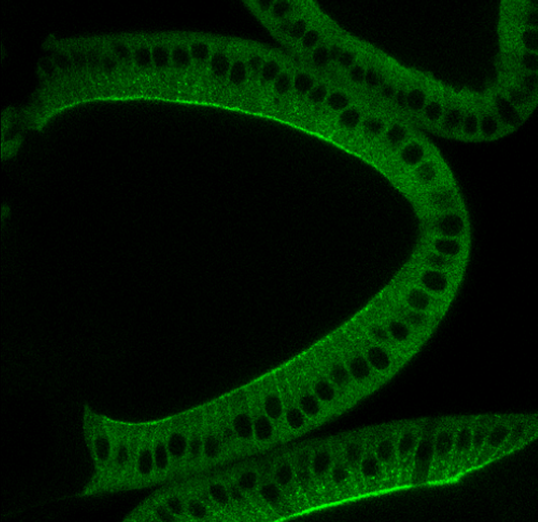

Supplement: Supplementary file 7 — Source Data for Figure 4 [file EMBJ-42-e112863-s004.zip › Fig4/Fig4B1.tif]

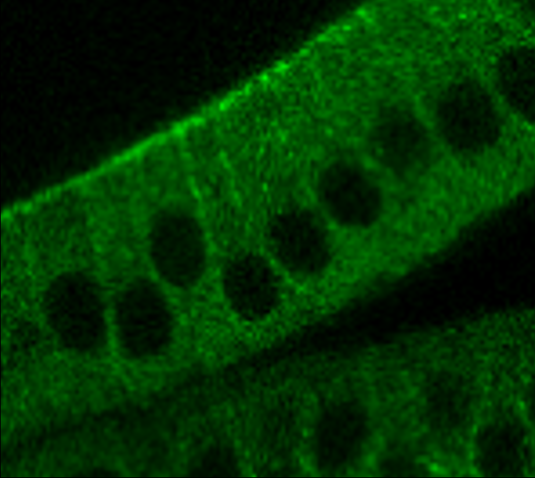

Supplement: Supplementary file 7 — Source Data for Figure 4 [file EMBJ-42-e112863-s004.zip › Fig4/Fig4B3.tif]

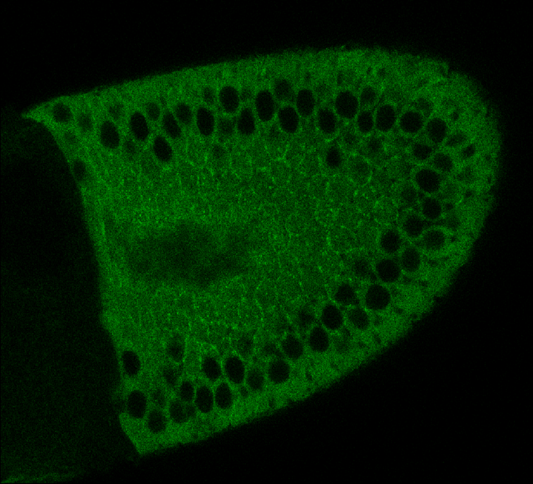

Supplement: Supplementary file 7 — Source Data for Figure 4 [file EMBJ-42-e112863-s004.zip › Fig4/Fig4B2.tif]

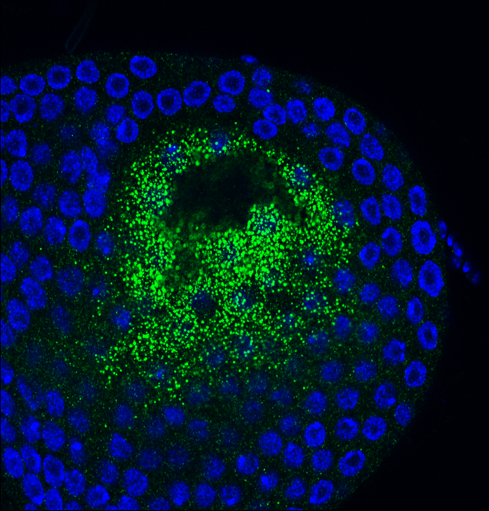

Supplement: Supplementary file 7 — Source Data for Figure 4 [file EMBJ-42-e112863-s004.zip › Fig4/Fig4C2.tif]

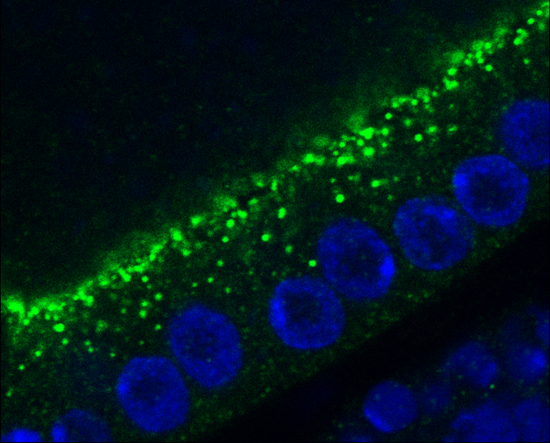

Supplement: Supplementary file 7 — Source Data for Figure 4 [file EMBJ-42-e112863-s004.zip › Fig4/Fig4C3.tif]

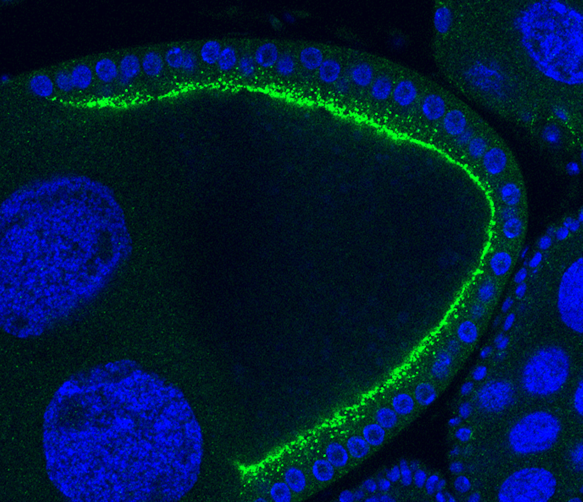

Supplement: Supplementary file 7 — Source Data for Figure 4 [file EMBJ-42-e112863-s004.zip › Fig4/Fig4C1.tif]

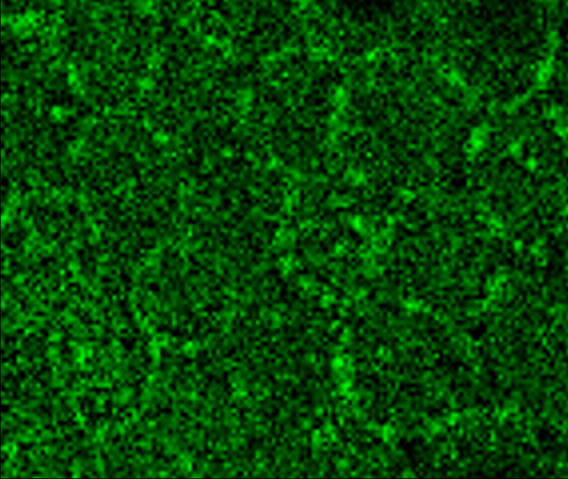

Supplement: Supplementary file 7 — Source Data for Figure 4 [file EMBJ-42-e112863-s004.zip › Fig4/Fig4B4.tif]

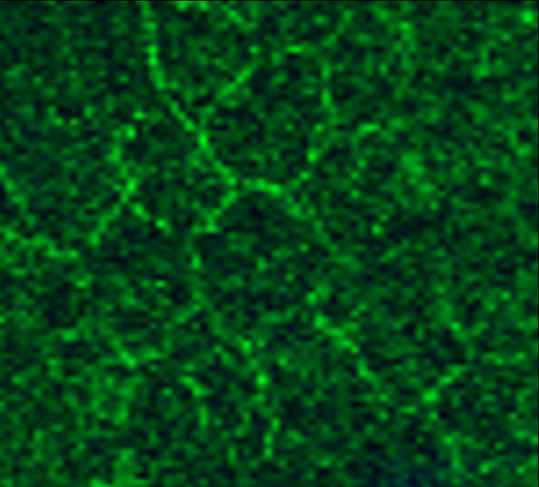

Supplement: Supplementary file 7 — Source Data for Figure 4 [file EMBJ-42-e112863-s004.zip › Fig4/Fig4A4.tif]

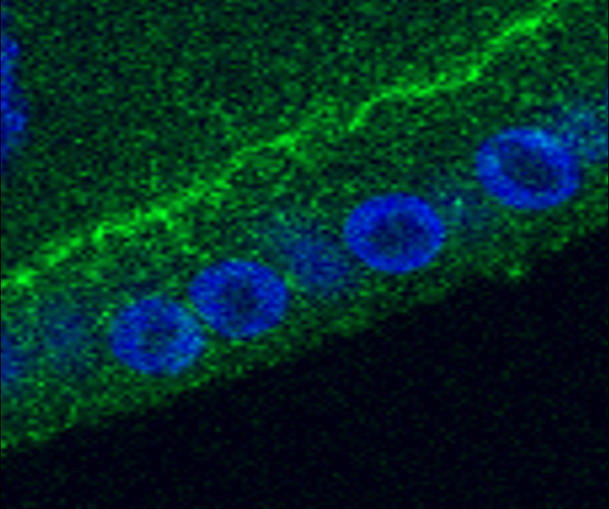

Supplement: Supplementary file 7 — Source Data for Figure 4 [file EMBJ-42-e112863-s004.zip › Fig4/Fig4A3.tif]

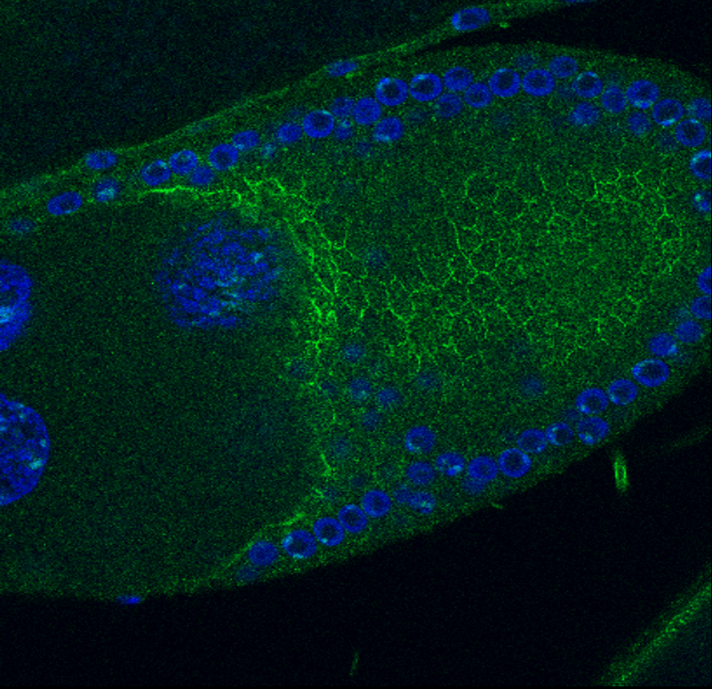

Supplement: Supplementary file 7 — Source Data for Figure 4 [file EMBJ-42-e112863-s004.zip › Fig4/Fig4A2.tif]

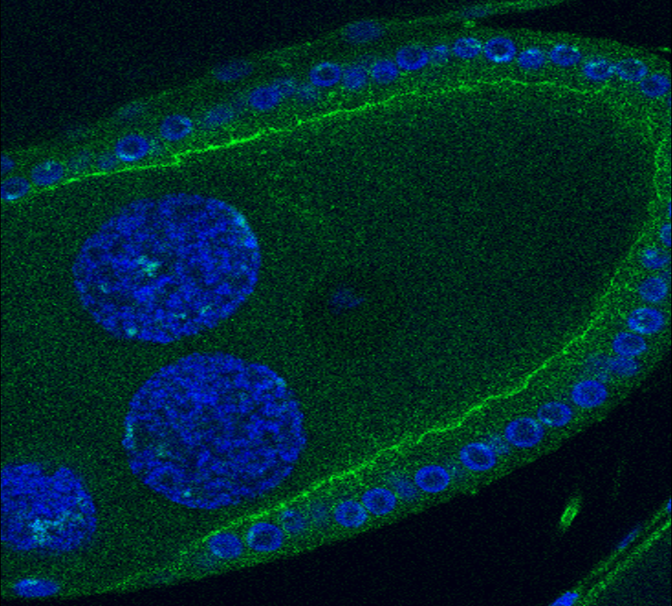

Supplement: Supplementary file 7 — Source Data for Figure 4 [file EMBJ-42-e112863-s004.zip › Fig4/Fig4A1.tif]

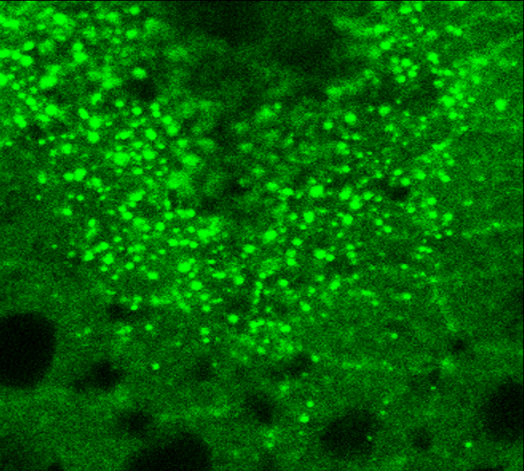

Supplement: Supplementary file 7 — Source Data for Figure 4 [file EMBJ-42-e112863-s004.zip › Fig4/Fig4F2.tif]

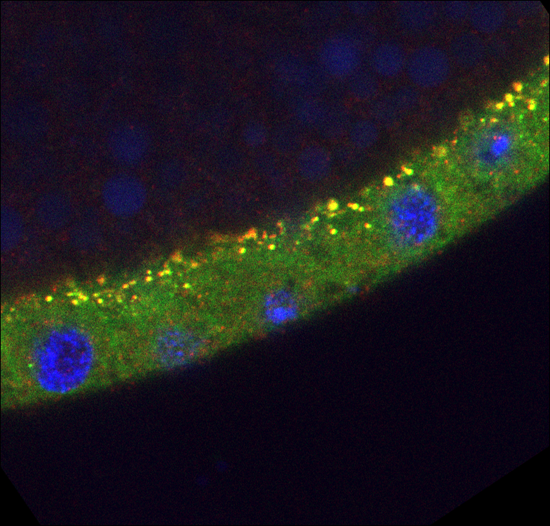

Supplement: Supplementary file 7 — Source Data for Figure 4 [file EMBJ-42-e112863-s004.zip › Fig4/Fig4F1.tif]

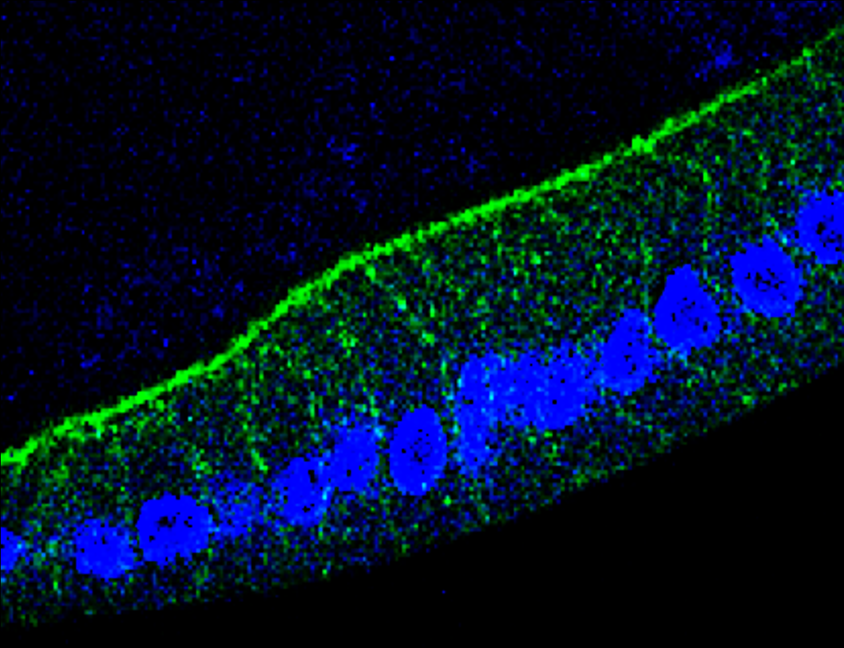

Supplement: Supplementary file 7 — Source Data for Figure 4 [file EMBJ-42-e112863-s004.zip › Fig4/Fig4G1.tif]

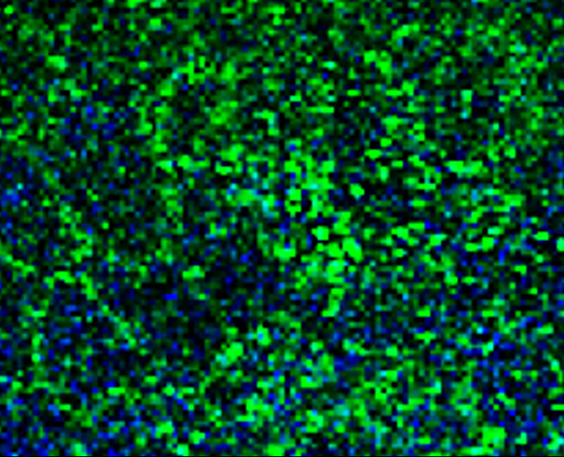

Supplement: Supplementary file 7 — Source Data for Figure 4 [file EMBJ-42-e112863-s004.zip › Fig4/Fig4G2.tif]

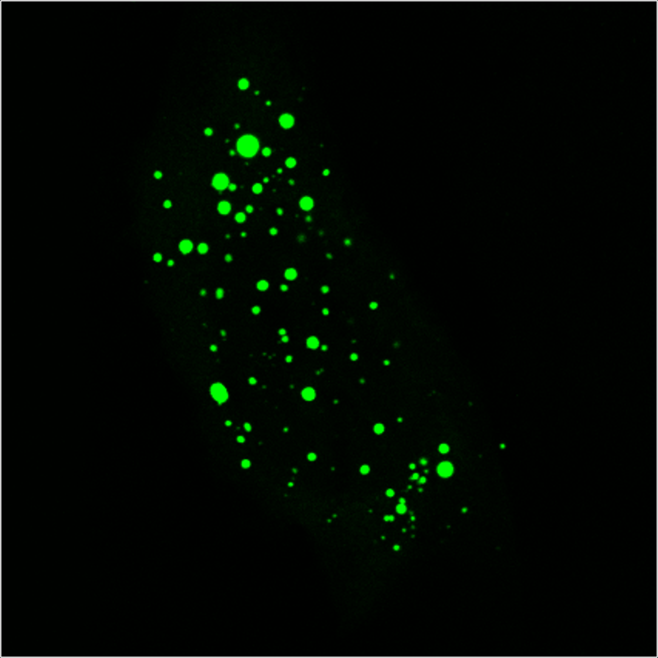

Supplement: Supplementary file 8 — Source Data for Figure 5 [file EMBJ-42-e112863-s009.zip › Fig5/Fig5B3.tif]

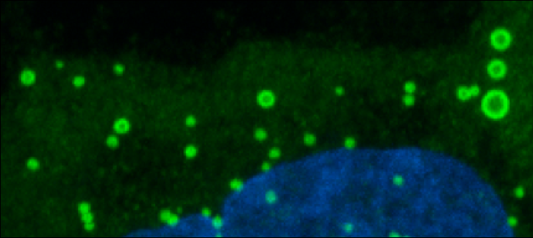

Supplement: Supplementary file 8 — Source Data for Figure 5 [file EMBJ-42-e112863-s009.zip › Fig5/Fig5B2.tif]

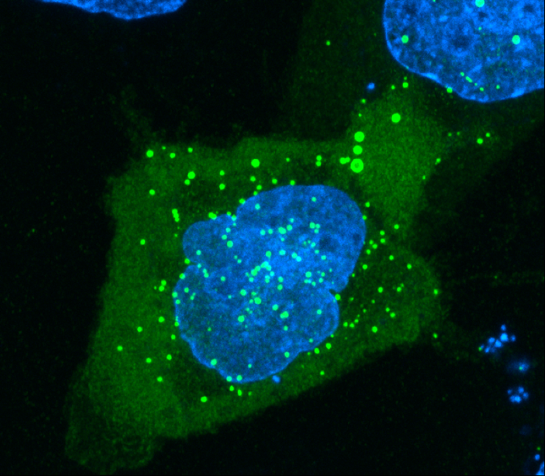

Supplement: Supplementary file 8 — Source Data for Figure 5 [file EMBJ-42-e112863-s009.zip › Fig5/Fig5B1.tif]

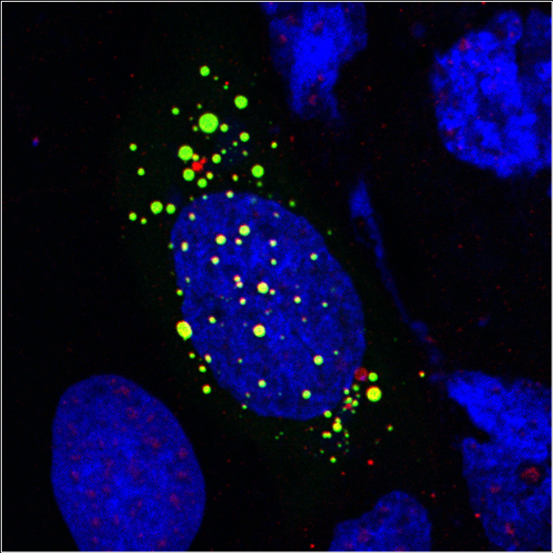

Supplement: Supplementary file 8 — Source Data for Figure 5 [file EMBJ-42-e112863-s009.zip › Fig5/Fig5B5.tif]

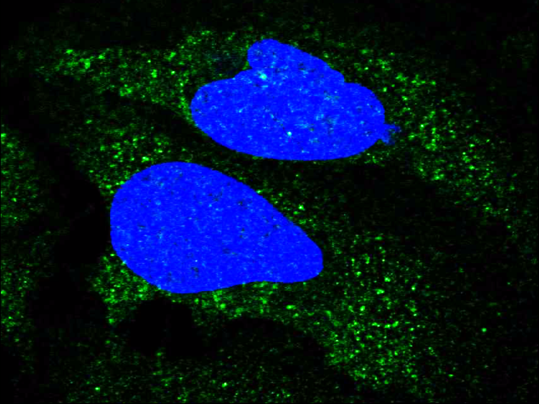

Supplement: Supplementary file 8 — Source Data for Figure 5 [file EMBJ-42-e112863-s009.zip › Fig5/Fig5A9.tif]

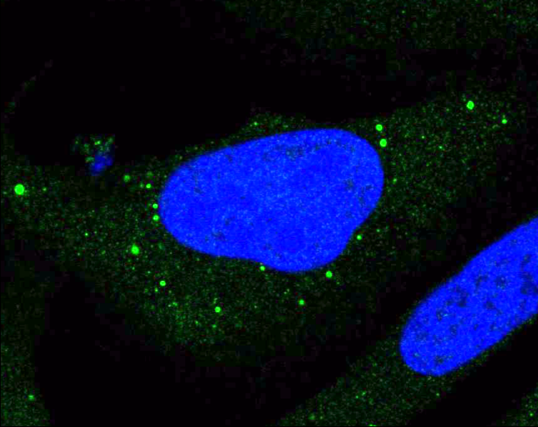

Supplement: Supplementary file 8 — Source Data for Figure 5 [file EMBJ-42-e112863-s009.zip › Fig5/Fig5A8.tif]

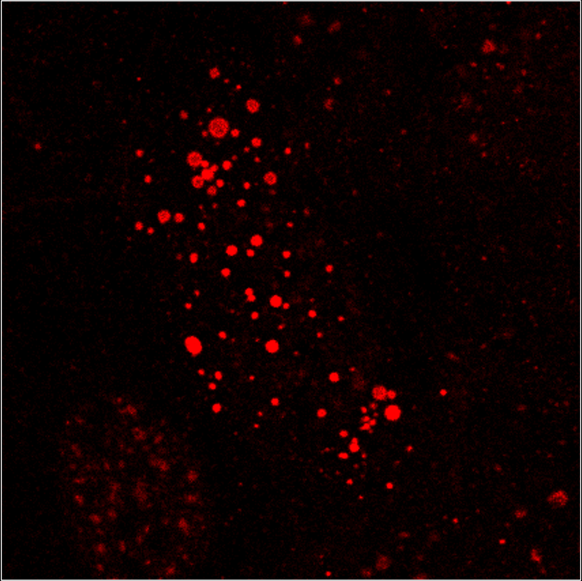

Supplement: Supplementary file 8 — Source Data for Figure 5 [file EMBJ-42-e112863-s009.zip › Fig5/Fig5B4.tif]

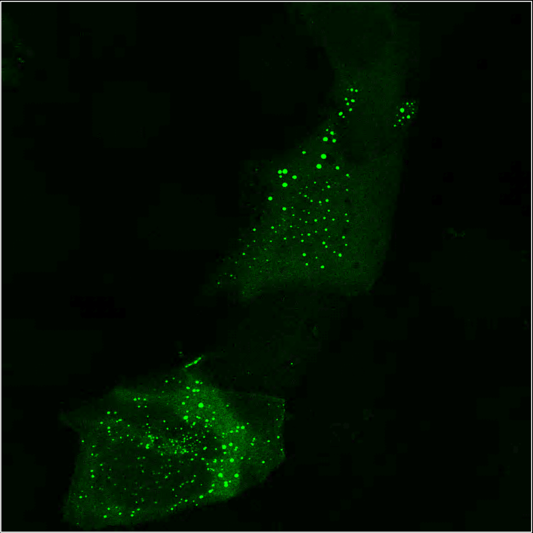

Supplement: Supplementary file 8 — Source Data for Figure 5 [file EMBJ-42-e112863-s009.zip › Fig5/Fig5B6.tif]

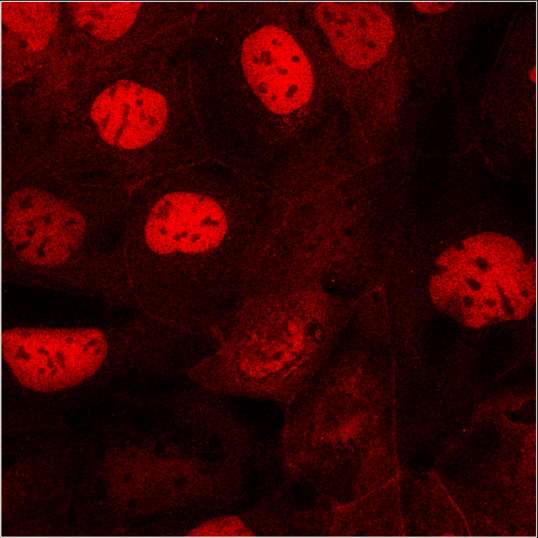

Supplement: Supplementary file 8 — Source Data for Figure 5 [file EMBJ-42-e112863-s009.zip › Fig5/Fig5B7.tif]

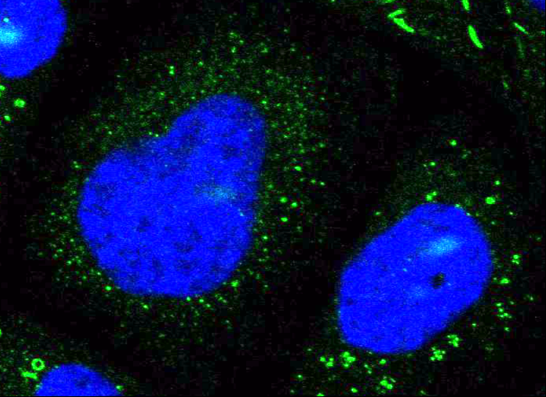

Supplement: Supplementary file 8 — Source Data for Figure 5 [file EMBJ-42-e112863-s009.zip › Fig5/Fig5A6.tif]

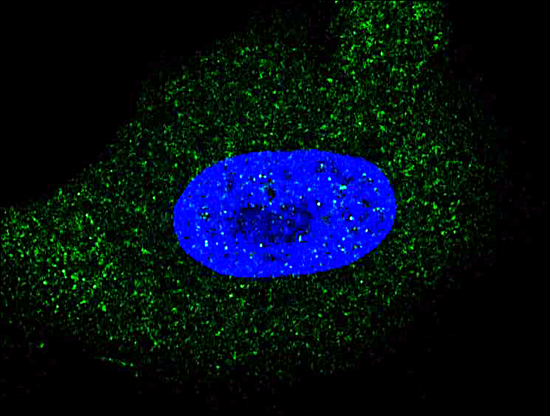

Supplement: Supplementary file 8 — Source Data for Figure 5 [file EMBJ-42-e112863-s009.zip › Fig5/Fig5A7.tif]

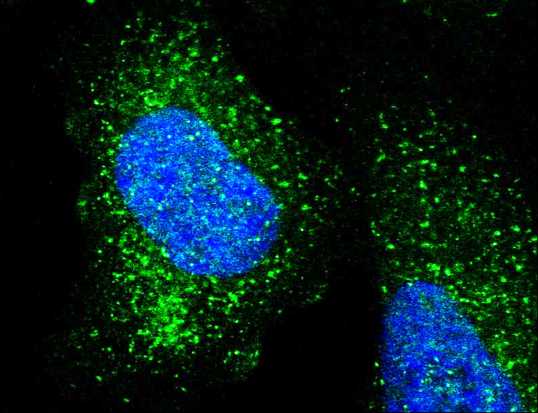

Supplement: Supplementary file 8 — Source Data for Figure 5 [file EMBJ-42-e112863-s009.zip › Fig5/Fig5A5.tif]

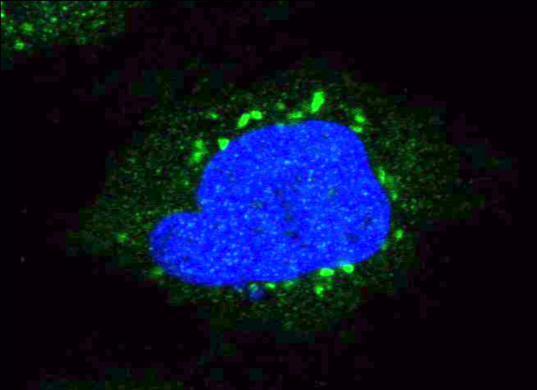

Supplement: Supplementary file 8 — Source Data for Figure 5 [file EMBJ-42-e112863-s009.zip › Fig5/Fig5A4.tif]

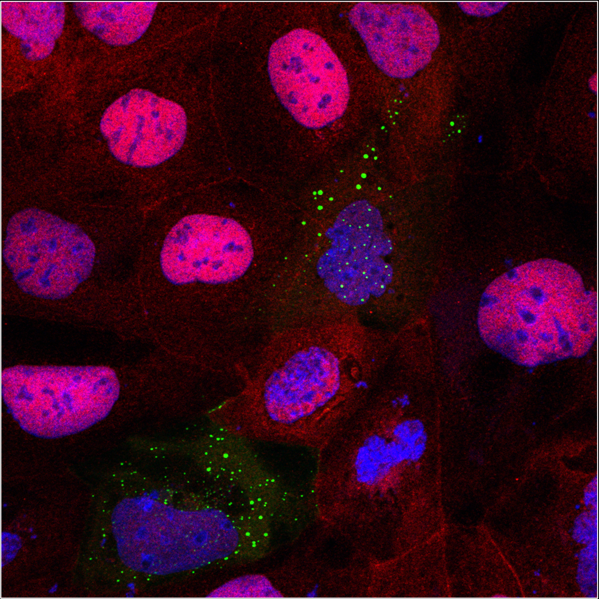

Supplement: Supplementary file 8 — Source Data for Figure 5 [file EMBJ-42-e112863-s009.zip › Fig5/Fig5B8.tif]

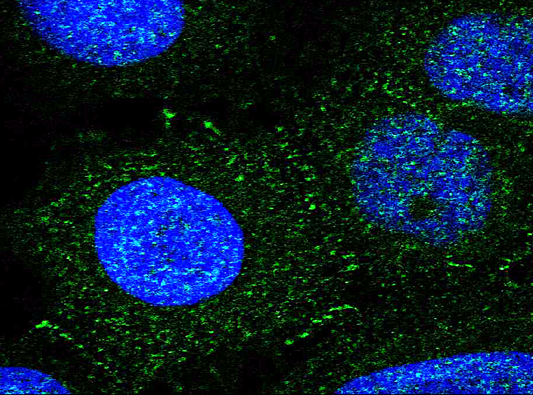

Supplement: Supplementary file 8 — Source Data for Figure 5 [file EMBJ-42-e112863-s009.zip › Fig5/Fig5A1.tif]

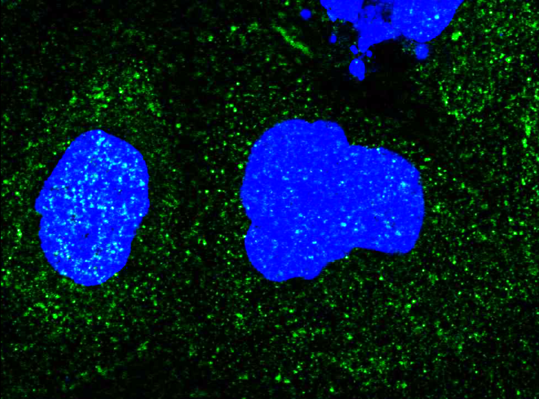

Supplement: Supplementary file 8 — Source Data for Figure 5 [file EMBJ-42-e112863-s009.zip › Fig5/Fig5A3.tif]

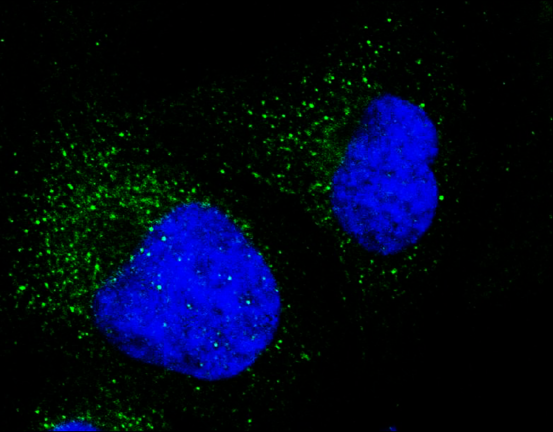

Supplement: Supplementary file 8 — Source Data for Figure 5 [file EMBJ-42-e112863-s009.zip › Fig5/Fig5A2.tif]

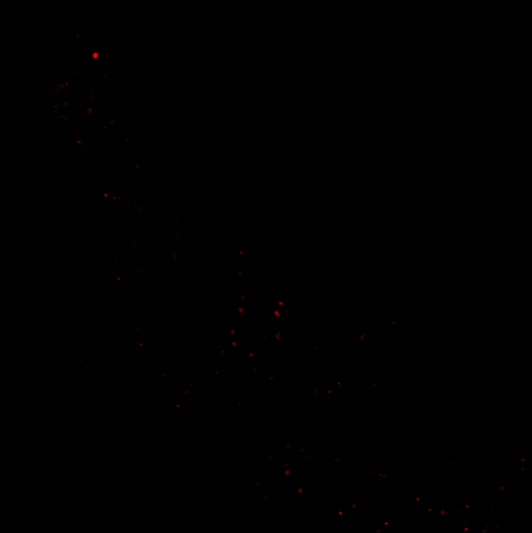

Supplement: Supplementary file 9 — Source Data for Figure 6 [file EMBJ-42-e112863-s008.zip › Fig6/Fig6A8.tif]

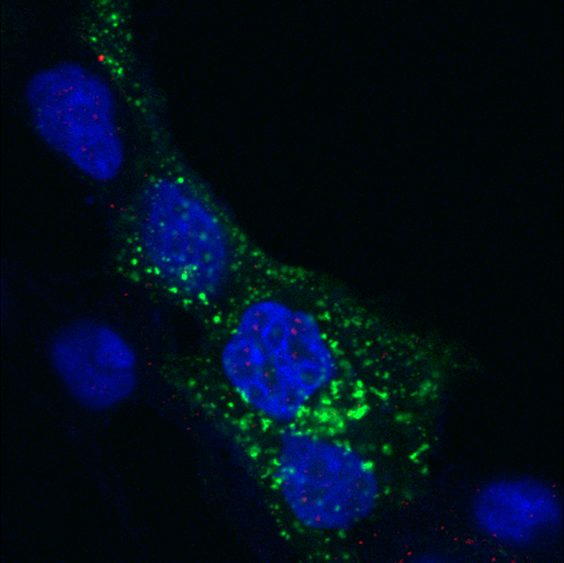

Supplement: Supplementary file 9 — Source Data for Figure 6 [file EMBJ-42-e112863-s008.zip › Fig6/Fig6A9.tif]

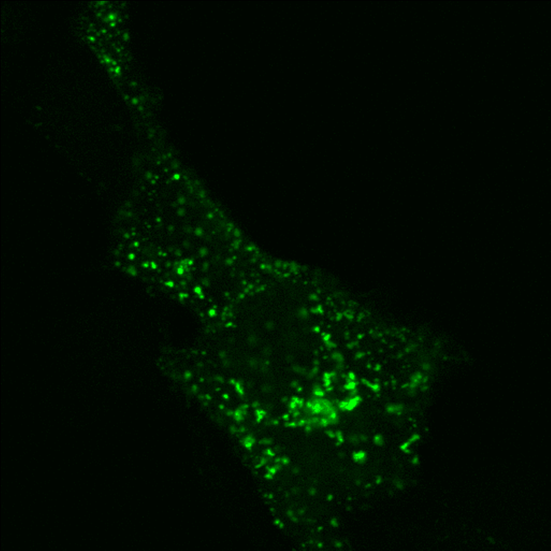

Supplement: Supplementary file 9 — Source Data for Figure 6 [file EMBJ-42-e112863-s008.zip › Fig6/Fig6A7.tif]

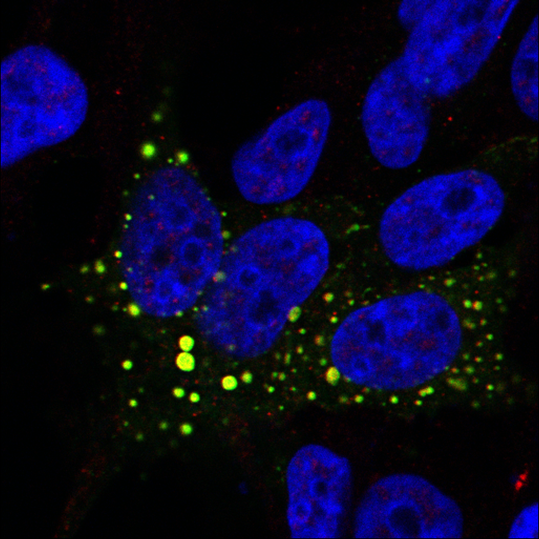

Supplement: Supplementary file 9 — Source Data for Figure 6 [file EMBJ-42-e112863-s008.zip › Fig6/Fig6A6.tif]

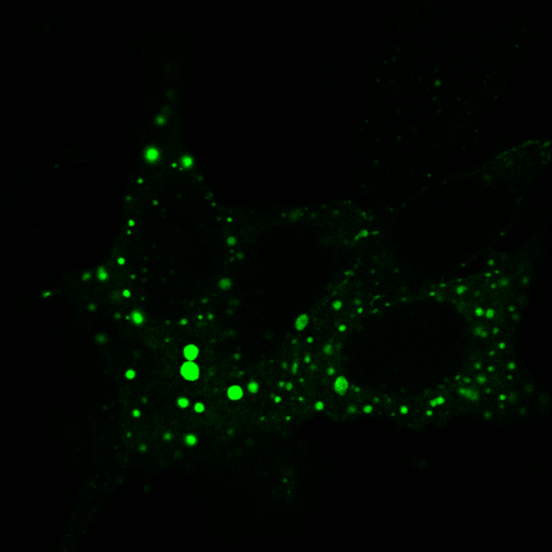

Supplement: Supplementary file 9 — Source Data for Figure 6 [file EMBJ-42-e112863-s008.zip › Fig6/Fig6A4.tif]

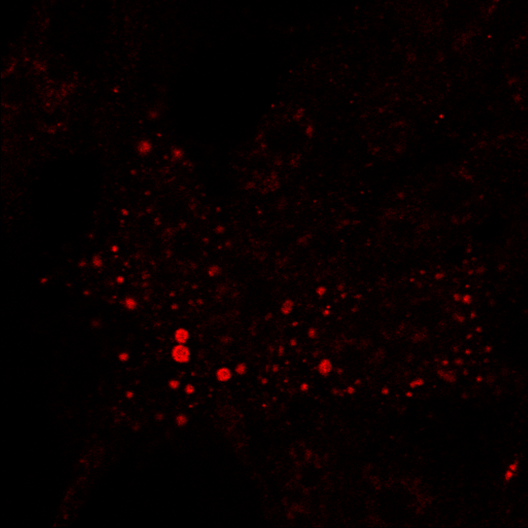

Supplement: Supplementary file 9 — Source Data for Figure 6 [file EMBJ-42-e112863-s008.zip › Fig6/Fig6A5.tif]

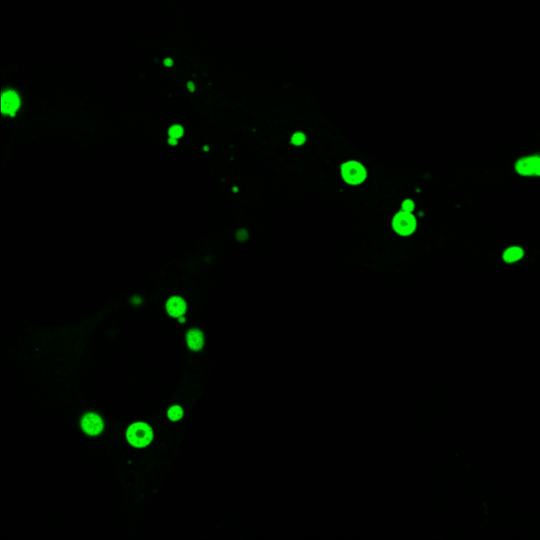

Supplement: Supplementary file 9 — Source Data for Figure 6 [file EMBJ-42-e112863-s008.zip › Fig6/Fig6A1.tif]

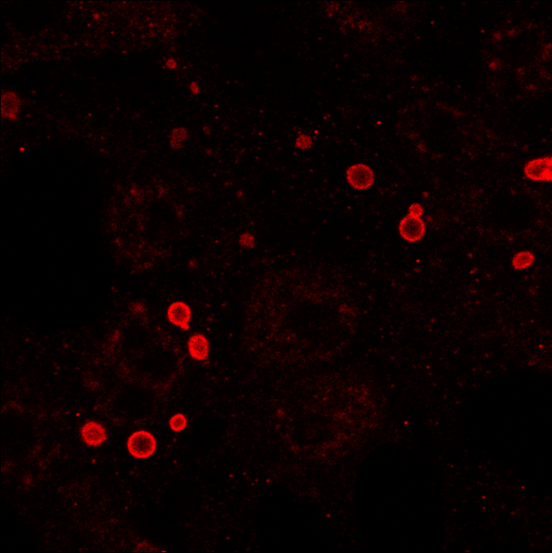

Supplement: Supplementary file 9 — Source Data for Figure 6 [file EMBJ-42-e112863-s008.zip › Fig6/Fig6A2.tif]

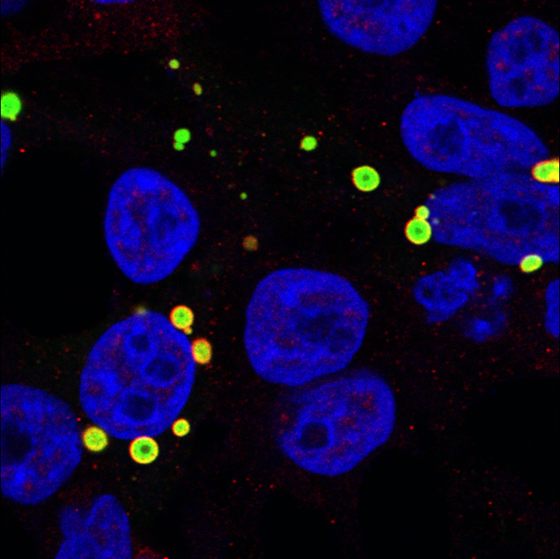

Supplement: Supplementary file 9 — Source Data for Figure 6 [file EMBJ-42-e112863-s008.zip › Fig6/Fig6A3.tif]

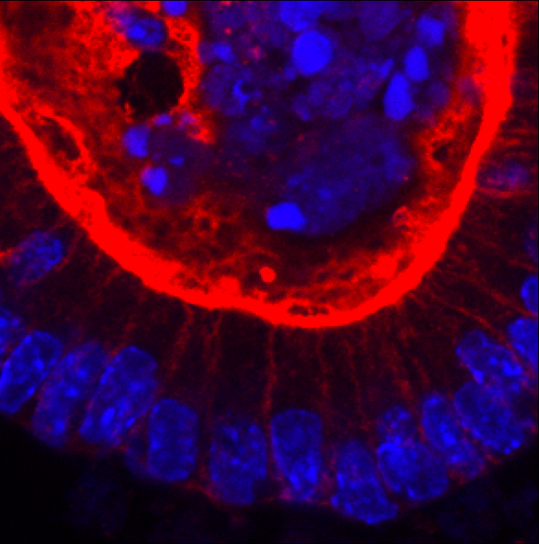

Supplement: Supplementary file 10 — Source Data for Figure 7 [file EMBJ-42-e112863-s002.zip › Fig7/Fig7B1.tif]

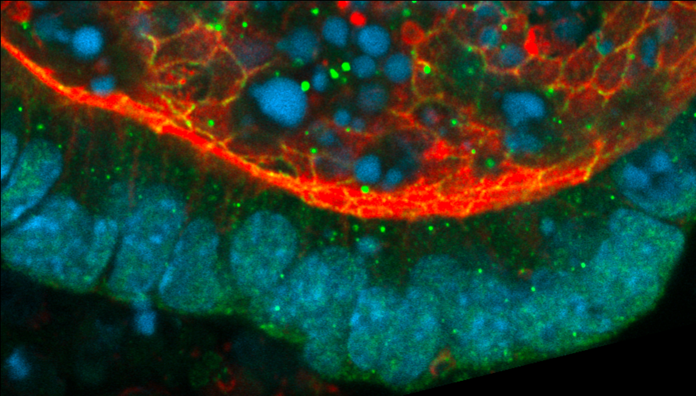

Supplement: Supplementary file 10 — Source Data for Figure 7 [file EMBJ-42-e112863-s002.zip › Fig7/Fig7C5.tif]

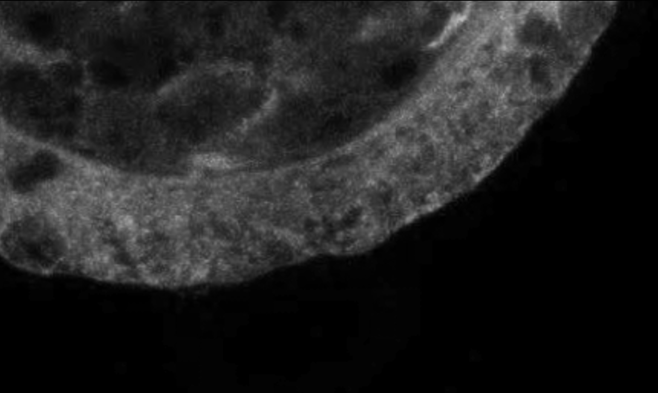

Supplement: Supplementary file 10 — Source Data for Figure 7 [file EMBJ-42-e112863-s002.zip › Fig7/Fig7C4.tif]

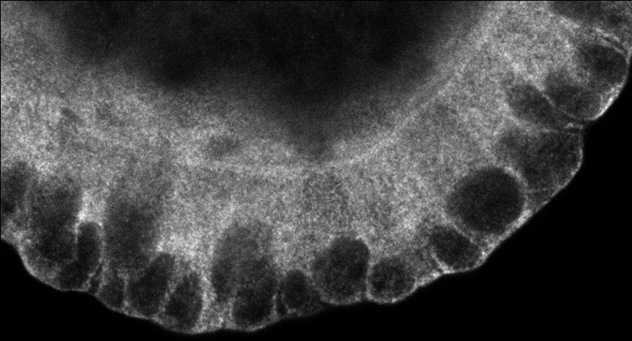

Supplement: Supplementary file 10 — Source Data for Figure 7 [file EMBJ-42-e112863-s002.zip › Fig7/Fig7C6.tif]

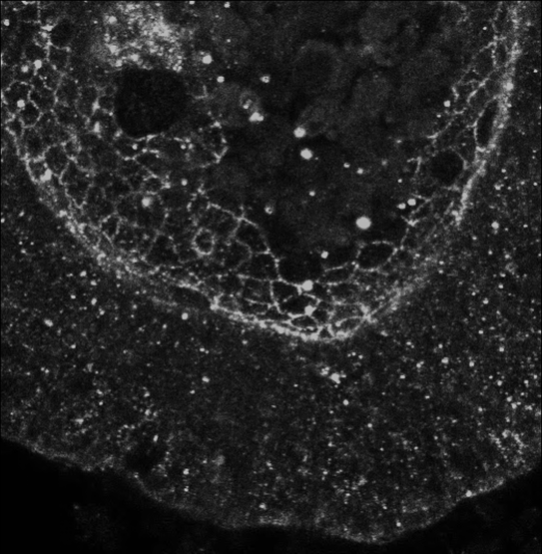

Supplement: Supplementary file 10 — Source Data for Figure 7 [file EMBJ-42-e112863-s002.zip › Fig7/Fig7B2.tif]

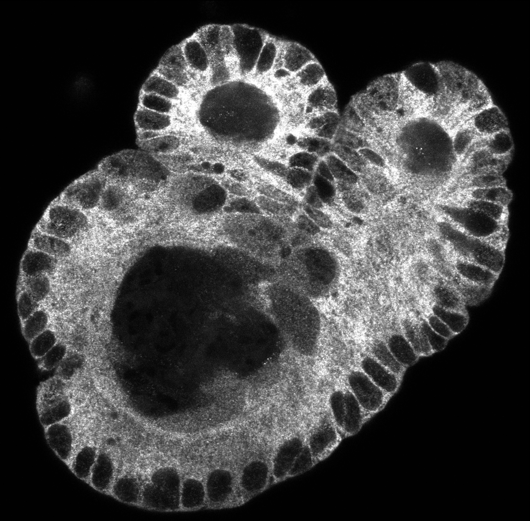

Supplement: Supplementary file 10 — Source Data for Figure 7 [file EMBJ-42-e112863-s002.zip › Fig7/Fig7B3.tif]

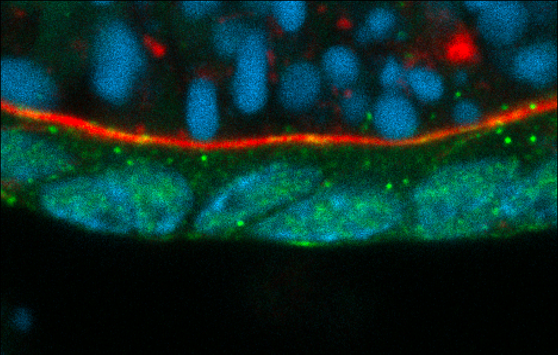

Supplement: Supplementary file 10 — Source Data for Figure 7 [file EMBJ-42-e112863-s002.zip › Fig7/Fig7C3.tif]

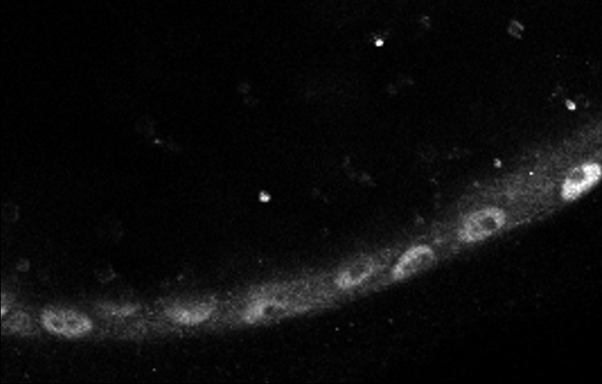

Supplement: Supplementary file 10 — Source Data for Figure 7 [file EMBJ-42-e112863-s002.zip › Fig7/Fig7C2.tif]

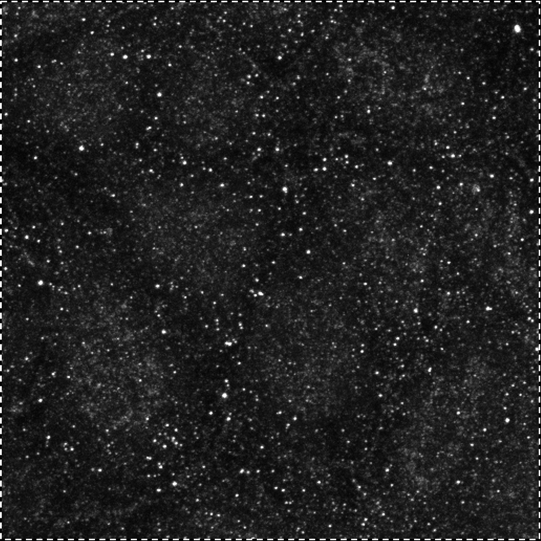

Supplement: Supplementary file 10 — Source Data for Figure 7 [file EMBJ-42-e112863-s002.zip › Fig7/Fig7A8.tif]

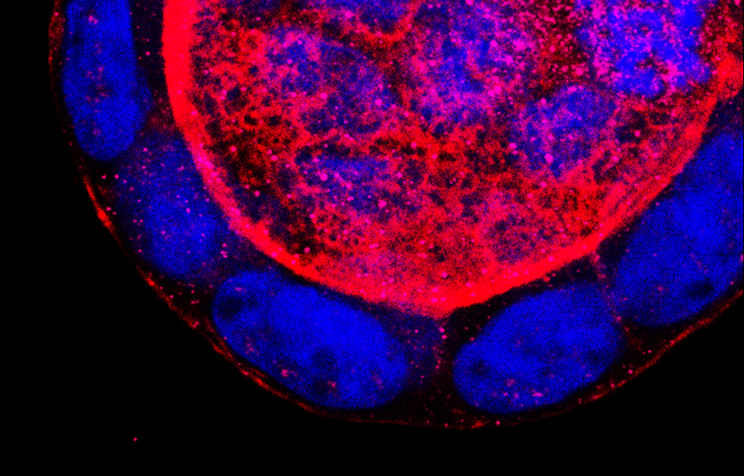

Supplement: Supplementary file 10 — Source Data for Figure 7 [file EMBJ-42-e112863-s002.zip › Fig7/Fig7A9.tif]

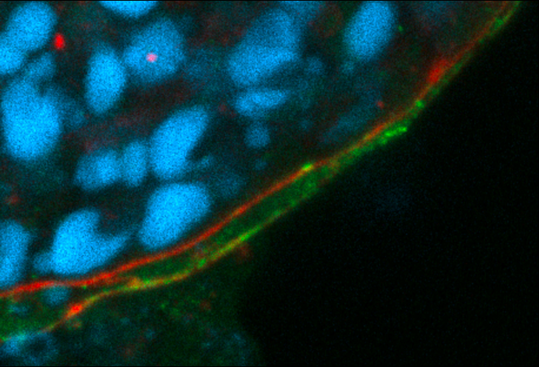

Supplement: Supplementary file 10 — Source Data for Figure 7 [file EMBJ-42-e112863-s002.zip › Fig7/Fig7C1.tif]

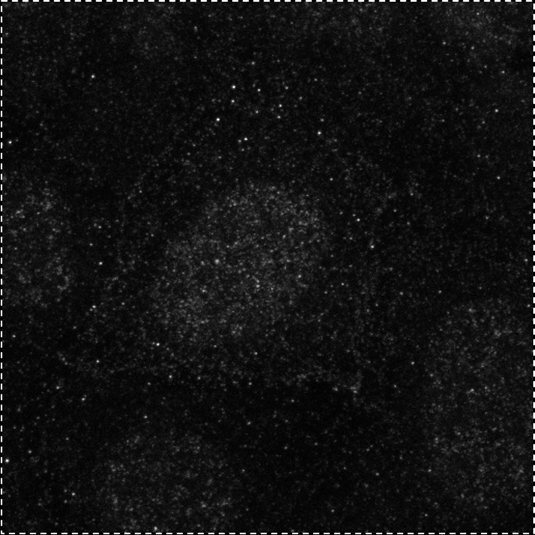

Supplement: Supplementary file 10 — Source Data for Figure 7 [file EMBJ-42-e112863-s002.zip › Fig7/Fig7A4.tif]

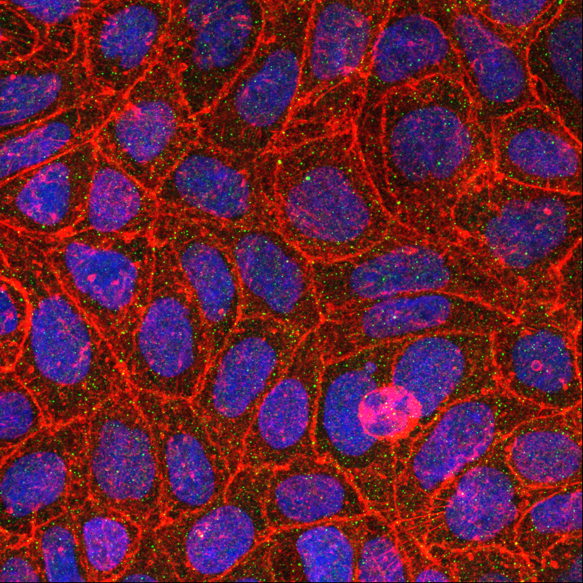

Supplement: Supplementary file 10 — Source Data for Figure 7 [file EMBJ-42-e112863-s002.zip › Fig7/Fig7A5.tif]

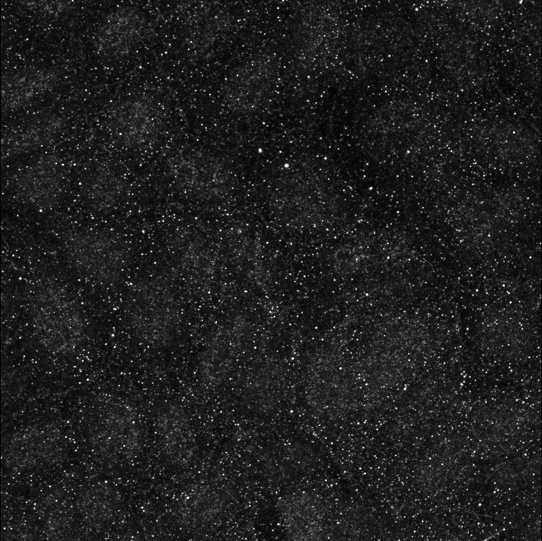

Supplement: Supplementary file 10 — Source Data for Figure 7 [file EMBJ-42-e112863-s002.zip › Fig7/Fig7A7.tif]

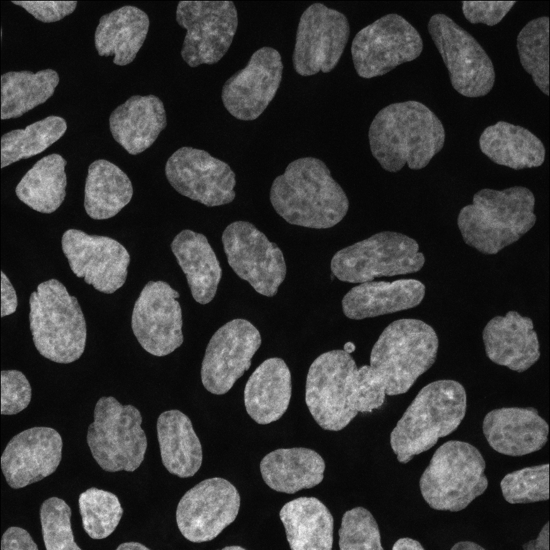

Supplement: Supplementary file 10 — Source Data for Figure 7 [file EMBJ-42-e112863-s002.zip › Fig7/Fig7A6.tif]

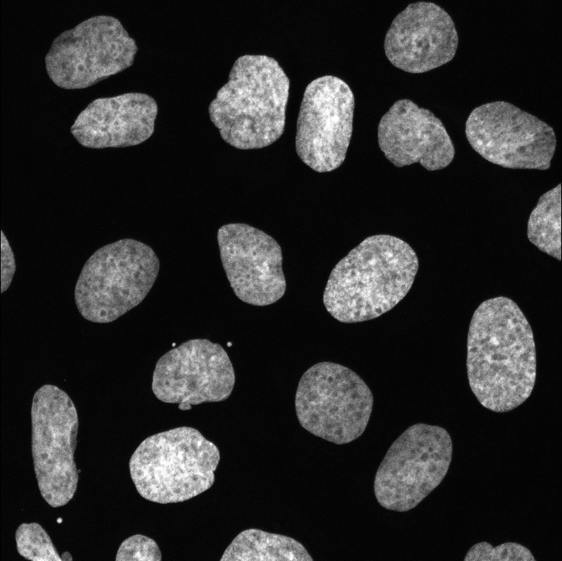

Supplement: Supplementary file 10 — Source Data for Figure 7 [file EMBJ-42-e112863-s002.zip › Fig7/Fig7A2.tif]

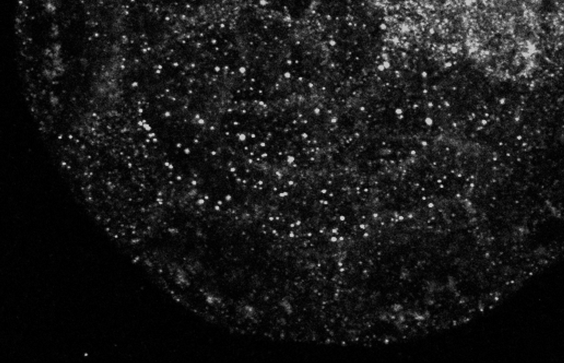

Supplement: Supplementary file 10 — Source Data for Figure 7 [file EMBJ-42-e112863-s002.zip › Fig7/Fig7A10.tif]

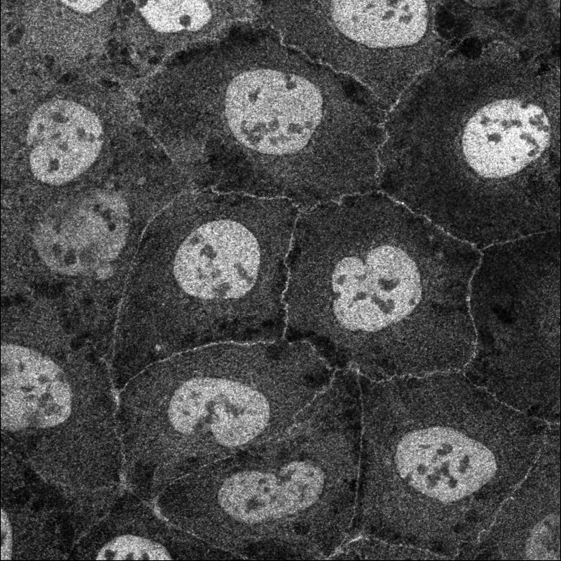

Supplement: Supplementary file 10 — Source Data for Figure 7 [file EMBJ-42-e112863-s002.zip › Fig7/Fig7A11.tif]

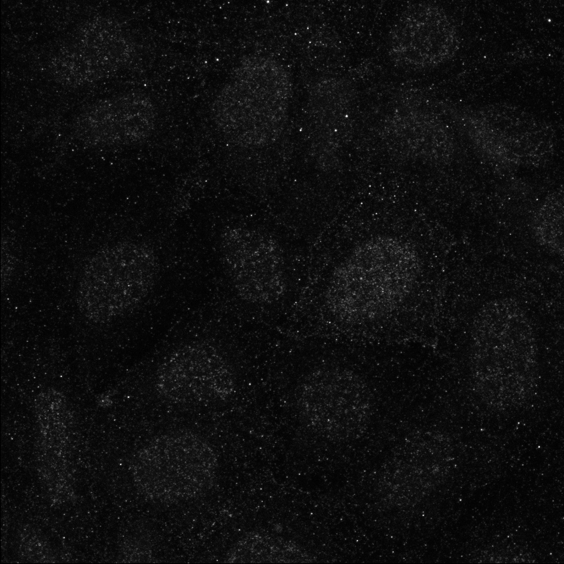

Supplement: Supplementary file 10 — Source Data for Figure 7 [file EMBJ-42-e112863-s002.zip › Fig7/Fig7A3.tif]

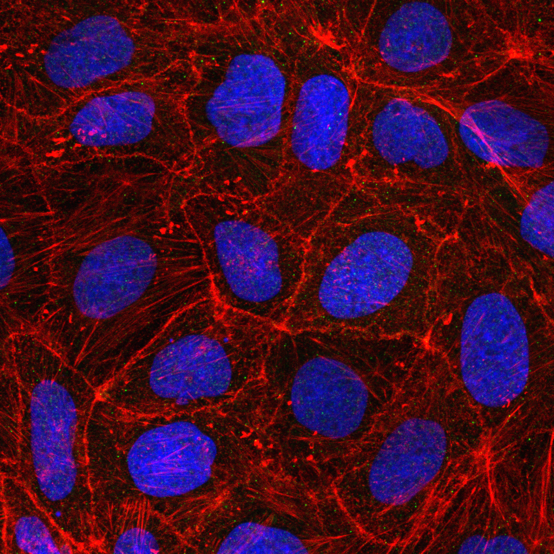

Supplement: Supplementary file 10 — Source Data for Figure 7 [file EMBJ-42-e112863-s002.zip › Fig7/Fig7A1.tif]

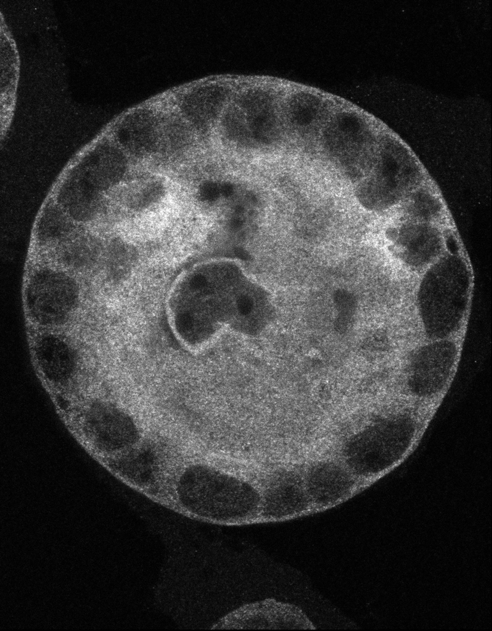

Supplement: Supplementary file 10 — Source Data for Figure 7 [file EMBJ-42-e112863-s002.zip › Fig7/Fig7A13.tif]

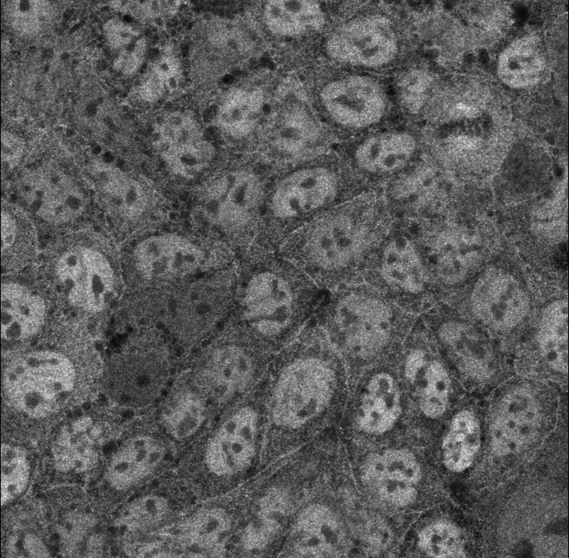

Supplement: Supplementary file 10 — Source Data for Figure 7 [file EMBJ-42-e112863-s002.zip › Fig7/Fig7A12.tif]

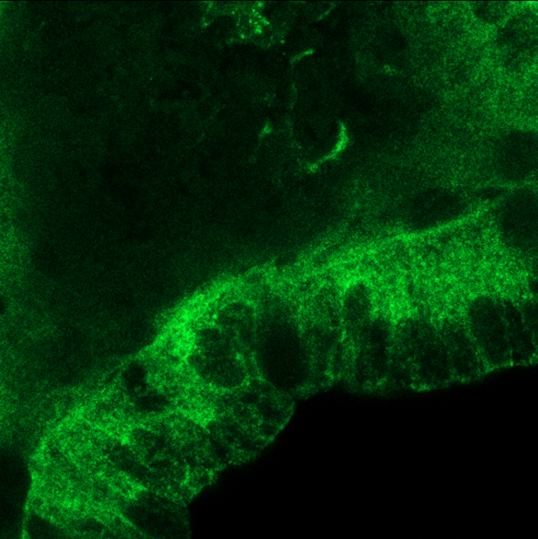

Supplement: Supplementary file 11 — Source Data for Figure 8 [file EMBJ-42-e112863-s006.zip › Fig8/Fig8B10.tif]
