# Supplementary figures and images for: Phase separation of Hippo signalling complexes (part 2 of 2)
Source: EMBO J. 2023 Feb 20;42(6):e112863. doi: 10.15252/embj.2022112863 (PMC10015380; doi:10.15252/embj.2022112863)

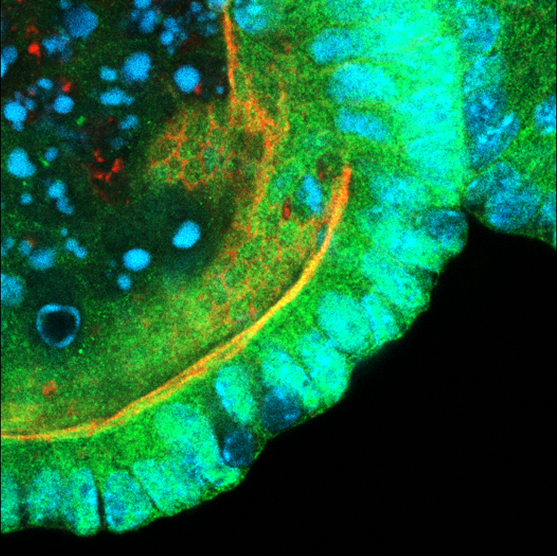

Supplement: Supplementary file 11 — Source Data for Figure 8 [file EMBJ-42-e112863-s006.zip › Fig8/Fig8B11.tif]

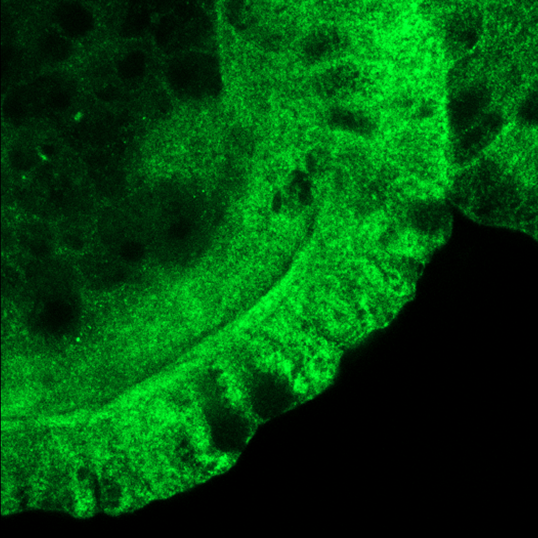

Supplement: Supplementary file 11 — Source Data for Figure 8 [file EMBJ-42-e112863-s006.zip › Fig8/Fig8B12.tif]

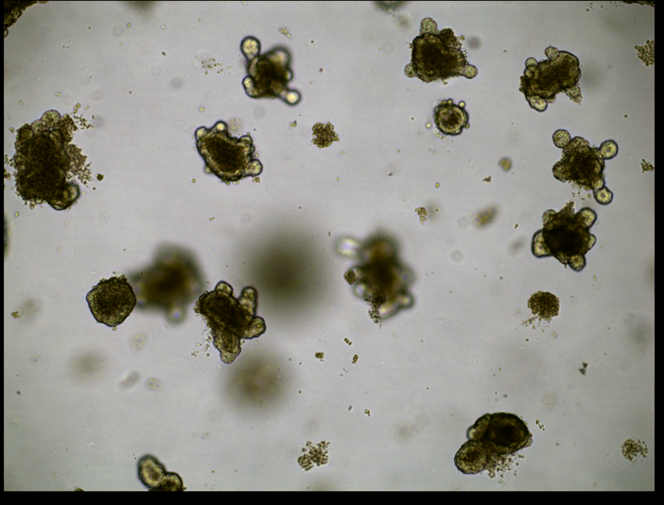

Supplement: Supplementary file 11 — Source Data for Figure 8 [file EMBJ-42-e112863-s006.zip › Fig8/Fig8A1.tif]

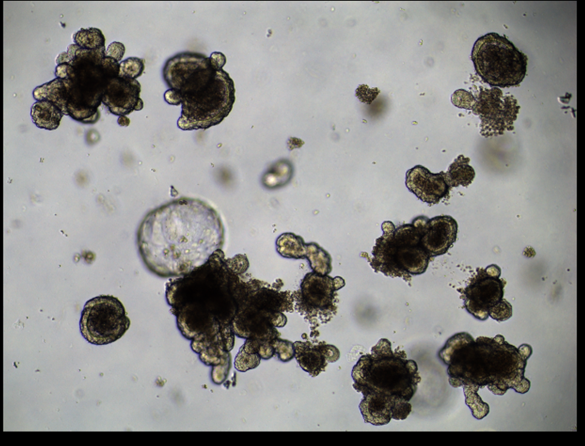

Supplement: Supplementary file 11 — Source Data for Figure 8 [file EMBJ-42-e112863-s006.zip › Fig8/Fig8A2.tif]

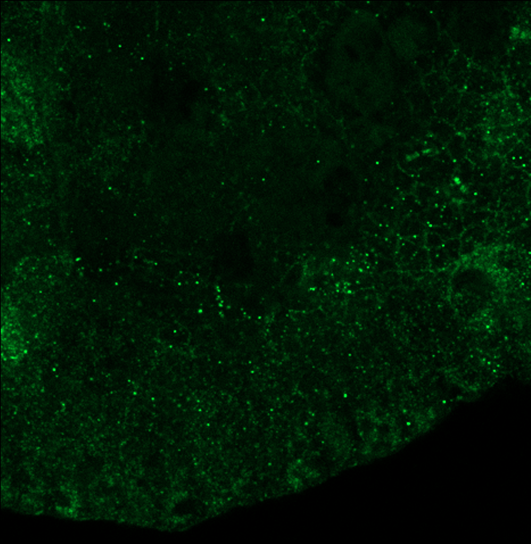

Supplement: Supplementary file 11 — Source Data for Figure 8 [file EMBJ-42-e112863-s006.zip › Fig8/Fig8B8.tif]

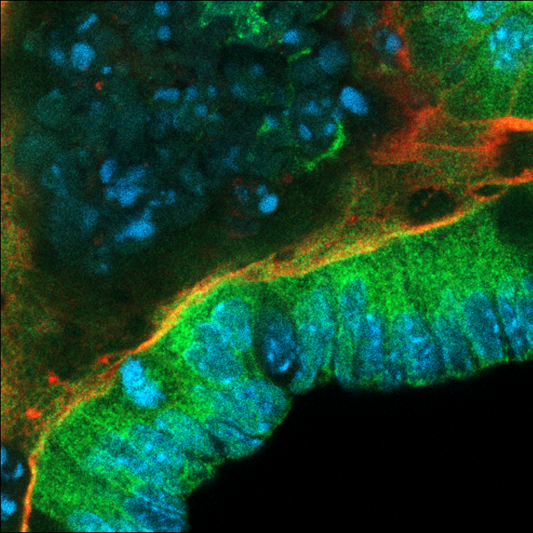

Supplement: Supplementary file 11 — Source Data for Figure 8 [file EMBJ-42-e112863-s006.zip › Fig8/Fig8B9.tif]

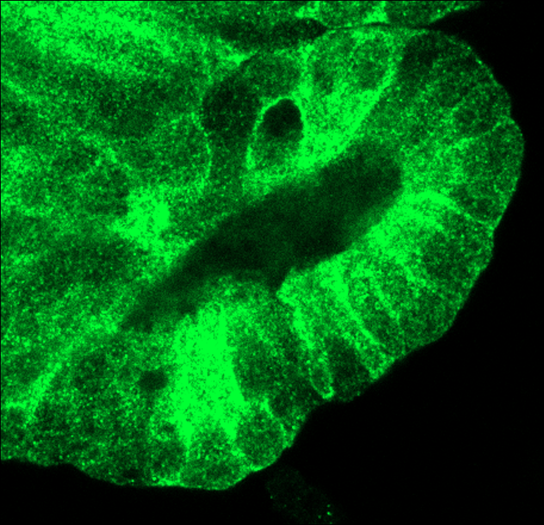

Supplement: Supplementary file 11 — Source Data for Figure 8 [file EMBJ-42-e112863-s006.zip › Fig8/Fig8B4.tif]

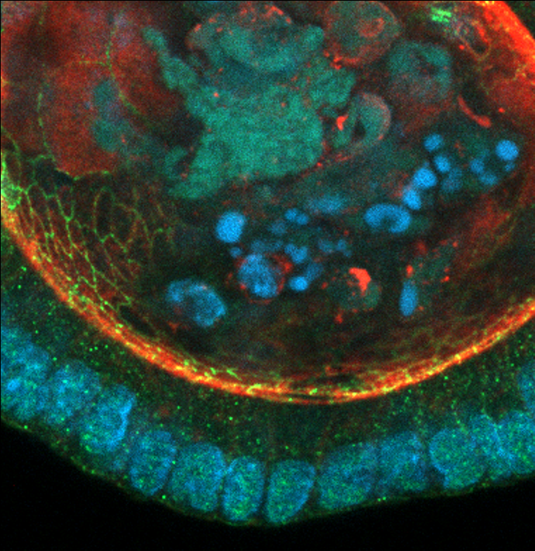

Supplement: Supplementary file 11 — Source Data for Figure 8 [file EMBJ-42-e112863-s006.zip › Fig8/Fig8B5.tif]

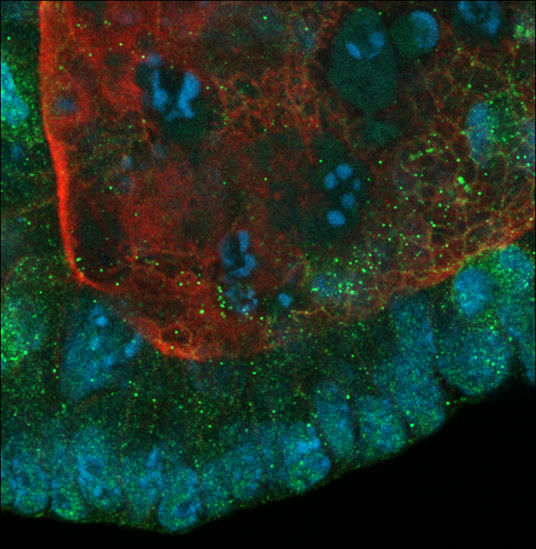

Supplement: Supplementary file 11 — Source Data for Figure 8 [file EMBJ-42-e112863-s006.zip › Fig8/Fig8B7.tif]

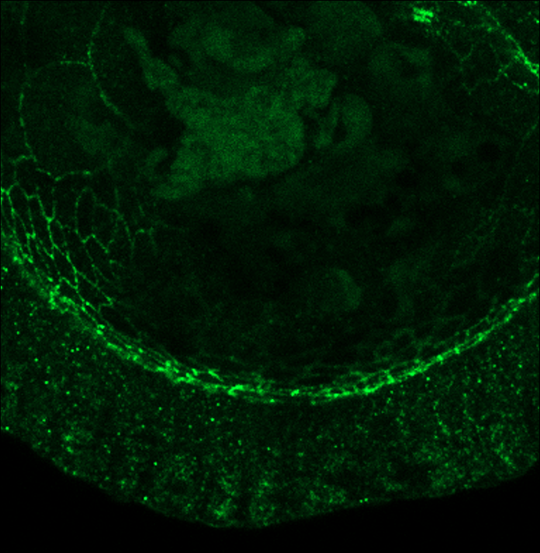

Supplement: Supplementary file 11 — Source Data for Figure 8 [file EMBJ-42-e112863-s006.zip › Fig8/Fig8B6.tif]

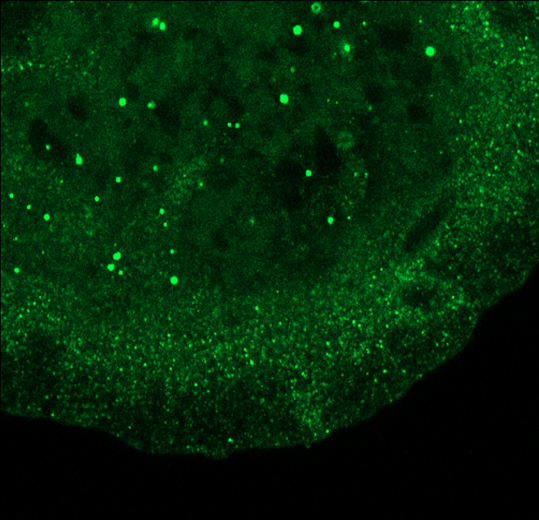

Supplement: Supplementary file 11 — Source Data for Figure 8 [file EMBJ-42-e112863-s006.zip › Fig8/Fig8B2.tif]

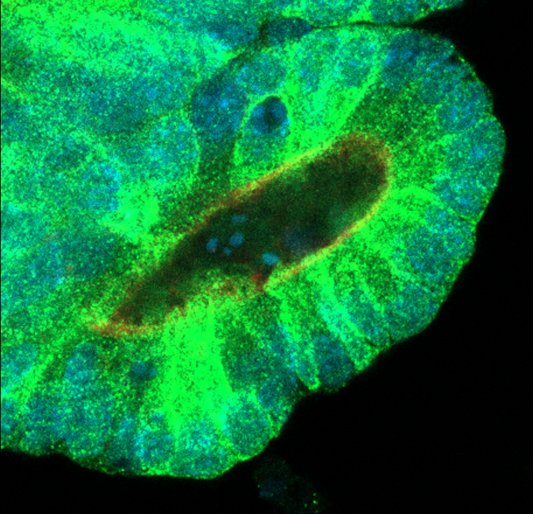

Supplement: Supplementary file 11 — Source Data for Figure 8 [file EMBJ-42-e112863-s006.zip › Fig8/Fig8B3.tif]

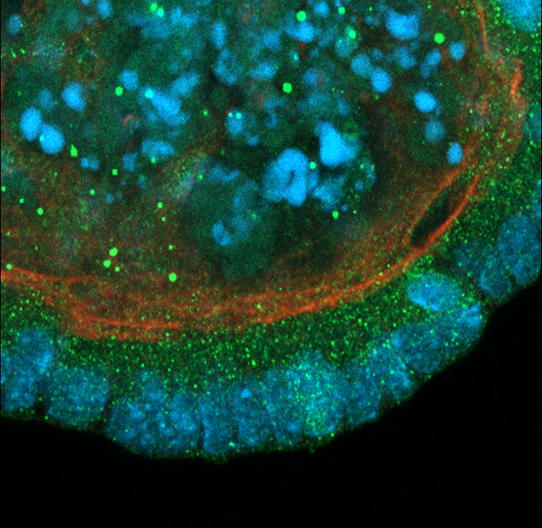

Supplement: Supplementary file 11 — Source Data for Figure 8 [file EMBJ-42-e112863-s006.zip › Fig8/Fig8B1.tif]
