# Supplementary material for: Phase separation of Hippo signalling complexes
Source: EMBO J. 2023 Feb 20;42(6):e112863. doi: 10.15252/embj.2022112863 (PMC10015380; doi:10.15252/embj.2022112863)
Supplement: Supplementary file 12 [file EMBJ-42-e112863-s003.pdf]

THE EMBO JOURNAL

Phase separation of Hippo signalling complexes

Teresa Bonello, Danfeng Cai, Georgina Fletcher, Kyler Weingartner, Victoria Pengilly, Kimberly Lange, Zhe Liu, Jennifer Lippincott-Schwartz, Jennifer Kavran, and Barry Thompson
DOI: 10.15252/embj.2022112863

Corresponding author: Barry Thompson (barry.thompson@anu.edu.au)

Review Timeline:

Submission Date:	18th Oct 22
Editorial Decision:	24th Nov 22
Revision Received:	18th Dec 22
Editorial Decision:	12th Jan 23
Revision Received:	12th Jan 23
Accepted:	23rd Jan 23

Editor: Ieva Gailite

Transaction Report:

(Note: Please note that the manuscript was transferred from another journal where it was originally reviewed. Since the original reviews are not subject to EMBO's transparent review process policy, the reports and author response cannot be published. With the exception of the correction of typographical or spelling errors that could be a source of ambiguity, letters and reports are not edited. Depending on transfer agreements, referee reports obtained elsewhere may or may not be included in this compilation. Referee reports are anonymous unless the Referee chooses to sign their reports.)

Thank you for submitting your manuscript together with the reviews from another journal and your point-by-point response to them to The EMBO Journal. I have now received a report from an arbitrating advisor, who has evaluated the revised manuscript. I have copied the advisor's comments below.

As you can see, the advisor finds the topic of interest, but also highlights that the study remains rather preliminary at this stage. From the editorial side, addressing the full functional relevance of Hippo pathway component condensate formation will not be required. However, further strengthening of the evidence for the biomolecular condensate properties of the observed puncta will be needed for acceptance here. In particular, please address the advisor's comments on Figures 2 and 3.

We generally allow three months as standard revision time. As a matter of policy, competing manuscripts published during this period will not negatively impact on our assessment of the conceptual advance presented by your study. However, please contact me as soon as possible upon publication of any related work to discuss the appropriate course of action. Should you foresee a problem in meeting this three-month deadline, please contact us to arrange an extension.

When preparing your letter of response to the referees' comments, please bear in mind that this will form part of the Review Process File and will therefore be available online to the community. For more details on our Transparent Editorial Process, please visit our website: https://www.embopress.org/page/journal/14602075/authorguide#transparentprocess. Please also see the attached instructions for further guidelines on preparation of the revised manuscript.

Please feel free to contact me if you have any questions regarding the revision. I would be happy to discuss the revision in more detail via email or phone/videoconferencing. Thank you for the opportunity to consider your work for publication, and I look forward to receiving the revised manuscript.

Advisor's comments:

The Hippo pathway is one of the central grow regulating signaling pathways in metazoan and well characterized on the molecular level. It involves membrane associated upstream kinases that signal to their interaction partners in the cytoplasm - Hippo being the central component - and the downstream kinases LATS1/2 regulating the nuclear shuttling behavior of the transcription factor YAP/TAZ. The very interesting aspect of that pathway is that it is able to integrate mechanical stress as well as integrate other signaling cues to regulate cell growth. Although we know the many players of the Hippo pathway, the key question remains how the many different signals from the different components are integrated to regulate cell proliferation. Anything that helps to answer this would be very exciting. In this paper the authors suggest a role for condensates, which is an exciting idea, and based on their initial data, likely to be a key component. However given that this would be somewhat of a paradigm shift in the Hippo field, it needs to be done properly.
I have read the paper and the reviews and have to agree with the reviewers comments. My viewpoint corresponds to the second paragraph in the report from reviewer 2. In addition, the figures come across as too preliminary.

So in summary, I think they have some interesting observation but don't really address how phase separated condensates could integrate cell signalling. I think they would have to completely rewrite and reassemble figures. It has many minor problems with the figures and labelling, that I did not really list but see below.

Overall it gives the impression of being just too preliminary. If they worked this up properly it could be a very interesting study.

Other points:

Figure 1 no scale bars

Mechanical manipulation of embryos would be a good experiment

Structuring of data is not well done. In the first results paragraph Fig S4 and Fig S5?

Figure 2 no scale bars either

Reversibility of treatment is missing, however one of the key hallmarks of biomolecular condensates. Why is this experiment not done with endogenously expressed Hippo?

Figure legends are missing information

2G: a valid hypothesis, but why is it not tested by experiments?

Figure 3: fusion events are studied with Hippo kinased dead overexpression construct, this is not a relevant construct to study physiological Hippo condensates. The title of the condensates infers Hpo condensates, but all that is studied is an overexpressed kinase dead construct.

The bleaching experiments are rather crude, the whole area is bleached, rather than the "condensate" A kymograph of the

shown image in B is missing, why? Because the are bleached is by far too big. How about bleaching only half of the "condensate"?

The shapes that are studied don't appear round but seem to be rather other forms of assemblies

Figure 4:

Description of experiments rather incomplete. What is live and what is fixed?

E/F: cannot be judged whether there is any colocalization

Analysis Hpo in human cell line CACO2 is missing

Overall comments: language is very imprecise when it comes to the main point: biomolecular condensates, condensates, punctae, assemblies,

"Together, these results show that mammalian Hpo signalling complexes form biomolecular condensates both in vitro and in vivo, and that those condensates contain active, phosphorylated, LATS kinase. Thus, mammalian Hpo signalling complexes undergo phase separation in a similar manner to those in Drosophila"

Although these statements may or may not be true we do not learn what the consequences are

Figure 8: figure legend is missing?!

Discussion:

"Our results demonstrate that Hpo kinase complexes undergo phase separation to form biomolecular condensates and implicate condensation as an important mechanism of Hpo signalling"

This is indeed not shown

The Hippo pathway is one of the central grow regulating signaling pathways in metazoan and well characterized on the molecular level. It involves membrane associated upstream kinases that signal to their interaction partners in the cytoplasm -Hippo being the central component - and the downstream kinases LATS1/2 regulating the nuclear shuttling behavior of the transcription factor YAP/TAZ. The very interesting aspect of that pathway is that it is able to integrate mechanical stress as well as integrate other signaling cues to regulate cell growth. Although we know the many players of the Hippo pathway, the key question remains how the many different signals from the different components are integrated to regulate cell proliferation. Anything that helps to answer this would be very exciting. In this paper the authors suggest a role for condensates, which is an exciting idea, and based on their initial data, likely to be a key component.

> We thank the arbitrating reviewer for their positive overall assessment of the research question and the exciting hypothesis that there is a role for condensates in signalling.

However given that this would be somewhat of a paradigm shift in the Hippo field, it needs to be done properly. I have read the paper and the reviews and have to agree with the reviewers comments. My viewpoint corresponds to the following comment from reviewer 2. In addition, the figures come across as too preliminary.
Referee 2: "Overall, although there is a body of published data showing that Hippo pathway components form obvious punctae in tissues and this manuscript investigates a plausible explanation for these punctae, it falls short of convincingly demonstrating that the observed punctae are biomolecular condensates or that pathway components normally form such condensates. Furthermore, it does not address a critical question - the functional significance of the observed ability to form punctae, either in terms of pathway activation or normal growth control. In sum, the observations reported here are too preliminary and do not sufficiently advance a mechanistic understanding of Hippo signaling to warrant publication at this time."
So in summary, I think they have some interesting observation but don't really address how phase separated condensates could integrate cell signalling. I think they would have to completely rewrite and reassemble figures. It has many minor problems with the figures and labelling, that I did not really list but see below. Overall it gives the impression of being just too preliminary. If they worked this up properly it could be a very interesting study.

> Referee 2's comments were based on the first version of the manuscript, submitted more than one year ago. The revised version has a large amount of new data that addresses the comments of all three Referees from Developmental Cell. We now also provide further additional new data and other refinements in the current version of our manuscript, our second revision (see below). In addition, a competing manuscript from DJ Pan, just published in Cell, also confirms our central findings that Hippo signalling complexes form condensates. The two papers are highly complementary, as the Cell paper has more in vitro analysis and our manuscript has more in vivo analysis. Together, the two papers make an overwhelming case for the importance of condensates in signalling.

To further address Referee 2's "critical question - the functional significance of the observed ability to form punctae … in terms of pathway activation" and "how phase separated condensates could integrate cell signalling" we provide new data. The new data show that Kibra-driven condensate formation is sufficient to activate signalling (measured by pLATS staining) only when Kibra contains long intrinsically disordered regions (IDRs) that promote phase separation. When the IDRs are deleted, Kibra is no longer able to form large condensates or to activate signalling. These new data provide additional evidence to directly link the propensity to drive condensate formation with signalling.

Other points:
Figure 1 no scale bars
> We apologise for this oversight. Scale bars are now added.

Mechanical manipulation of embryos would be a good experiment
> We agree with the reviewer that adding the mechanically stretched cells to this figure is worthwhile, and we now provide this data in the revised Figure 1D group of panels. We previously showed that mechanical stretching of the follicular epithelium occurs simply via growth of the oocyte (Fletcher et al Development).

Structuring of data is not well done. In the first results paragraph Fig S4 and Fig S5?
> To clarify, the first results paragraph references only Figure 1A and Fig S1. The second results paragraph mentions Fig 1A-E and Figs S2, S3, S4 & S5. Nevertheless, we take the reviewer's broader point, and to address this issue we have combined the previous Figs S1-S5 into a revised Fig S1 and S2 to ensure the supplementary figures are succinct and accord to EMBO J guidelines.

Figure 2 no scale bars either
> We apologise for this oversight. Scale bars are now added.

Reversibility of treatment is missing, however one of the key hallmarks of biomolecular condensates. Why is this experiment not done with endogenously expressed Hippo?
> To clarify, reversibility of this treatment was shown in Fig S17. It may have been difficult to find because it was buried deep in the supplementary figures. To address this point, we have now incorporated this data into a revised Fig S3, along with hexanediol treatment of endogenously expressed Hippo, so that these data are much easier for readers to locate.

Figure legends are missing information
> We have extended the figure legend accordingly.

2G: a valid hypothesis, but why is it not tested by experiments?
> We thank the reviewer for raising this issue. While there are experiments in the supplementary figures to support this schematic diagram, we understand the potential confusion. Thus, we have now removed this diagram from the revised Fig 2.

Figure 3: fusion events are studied with Hippo kinased dead overexpression construct, this is not a relevant construct to study physiological Hippo condensates. The title of the condensates infers Hpo condensates, but all that is studied is an overexpressed kinase dead construct.
> To address this point, we have corrected the title of the fusion panels (new Fig 2H). Live imaging of the endogenous Hpo-YFP was not possible owing to its low level of expression and rapid photobleaching. However, our evidence indicates that the HpoKD-Venus sensor is a reliable read-out of physiological Hippo pathway activity, and is sufficiently bright and photo-stable for live-imaging in vivo.

The bleaching experiments are rather crude, the whole area is bleached, rather than the "condensate" A kymograph of the shown image in B is missing, why? Because the are bleached is by far too big. How about bleaching only half of the "condensate"?
> The purpose of these FRAP experiments was to measure dynamic exchange of molecules between the condensate and the whole cytoplasm. It is therefore necessary to bleach the entire condensate but not the rest of the cellular cytoplasm, which is the experiment shown. The reviewer proposes a different type of experiment, which is to bleach half of the condensate, which could recover by exchange of molecules with either the cytoplasm or the other half of the condensate. It is not clear what additional information is derived from bleaching half of the condensate, so we instead provide an alternative type of experimental data to characterise the nature of these condensates (new Fig 6).

The shapes that are studied don't appear round but seem to be rather other forms of assemblies

> The reviewer appears to refer to the few small Hpo^{KD}-Venus punctae selected for live imaging, rather than the many larger and clearly spherical punctae that appear upon starvation (e.g.: Figs 1B, 2D or 4A) or upon overexpression of upstream pathway components (Fig 3C-H). It is always easier to observe a smooth spherical phase boundary with larger punctae, as these are more easily resolved under the microscope. Since molecular clusters typically only form an easily observable phase boundary above a critical size threshold, our observations with Hippo signalling complexes are consistent with the known principles of phase separation.

We note that mammalian condensates behave similarly, with small irregular punctae visible when Hippo signalling is OFF (e.g: new Fig 5A) and larger more spherical punctae visible when Hippo signalling is ON (new Fig 5B,C & 7A). Our new data (new Fig 6) also show that the intrinsically disordered regions (IDRs) in Kibra and SAV1 are required for them to nucleate large spherical punctae and for consequent signalling to pLATS.

Figure 4:
Description of experiments rather incomplete. What is live and what is fixed?

> These are fixed samples. We have updated the figure legend to clarify this point.

E/F: cannot be judged whether there is any colocalization

> To clarify, there is no colocalization between Crb (red) and Hpo^{KD}-Venus (green), while there is substantial colocalization between Ex and Hpo^{KD}-Venus (overlap in yellow punctae) in Fig 3E. Similarly, there is substantial colocalization between Ex and p-Wts (overlap in yellow punctae) in Fig 3F.

Analysis Hpo in human cell line CACO2 is missing

> The names of the two human Hpo kinases are MST1 & MST2. Our data show that MST2 colocalizes with SAV1 in punctae in the cytoplasm (see Fig S5D,E).

Overall comments: language is very imprecise when it comes to the main point: biomolecular condensates, condensates, punctae, assemblies,

> We have revised the text to improve precision in the use of these terms, specifically using punctae to refer to any microscopy data.

"Together, these results show that mammalian Hpo signalling complexes form biomolecular condensates both in vitro and in vivo, and that those condensates contain active, phosphorylated, LATS kinase. Thus, mammalian Hpo signalling complexes undergo phase separation in a similar manner to those in Drosophila"

Although these statements may or may not be true we do not learn what the consequences are

> To address this point, we provide new data. The new data show that Kibra-driven condensate formation is sufficient to activate signalling (measured by pLATS staining) only when Kibra contains long intrinsically disordered regions (IDRs) that promote phase separation. When the IDRs are deleted, Kibra is no longer able to form large condensates or to activate signalling (new Fig 6A-D). These new data provide additional evidence to directly link the propensity to drive condensate formation with signalling.

Figure 8: figure legend is missing?!

> We apologise for this error, which was due to a versioning conflict. We now provide the Figure 8 legend.

Discussion:

"Our results demonstrate that Hpo kinase complexes undergo phase separation to form biomolecular condensates and implicate condensation as an important mechanism of Hpo signalling"

This is indeed not shown

> We have revised this particular sentence to soften the tone. In addition, the new data we provide in the revised Figure 6 shows that IDRs and phase separation are required for Hippo signalling to phosphorylate LATS, which directly links phase separation with signalling.

Thank you for submitting a revised version of your manuscript. I have looked into your response and I find it reasonable. There now remain only a few editorial points that have to be addressed before I can extend formal acceptance of the manuscript.

Please let me know if you have any questions regarding any of these points. You can use the link below to upload the revised files.

Thank you again for giving us the chance to consider your manuscript for The EMBO Journal. I look forward to receiving the final version.

The authors addressed the minor editorial issues.

Editor accepted the revised manuscript.

EMBO Press Author Checklist

Corresponding Author Name: Barry James Thompson
Journal Submitted to: EMBO Journal
Manuscript Number: EMBOJ-2022-112863

USEFUL LINKS FOR COMPLETING THIS FORM
The EMBO Journal - Author Guidelines
EMBO Reports - Author Guidelines
Molecular Systems Biology - Author Guidelines
EMBO Molecular Medicine - Author Guidelines

Reporting Checklist for Life Science Articles (updated January 2022)
This checklist is adapted from Materials Design Analysis Reporting (MDAR) Checklist for Authors. MDAR establishes a minimum set of requirements in transparent reporting in the life sciences (see Statement of Task: 10.31222/osf.io/9sm4x). Please follow the journal's guidelines in preparing your manuscript.
Please note that a copy of this checklist will be published alongside your article.

Abridged guidelines for figures
1. Data
The data shown in figures should satisfy the following conditions:
- the data were obtained and processed according to the field's best practice and are presented to reflect the results of the experiments in an accurate and unbiased manner.
- ideally, figure panels should include only measurements that are directly comparable to each other and obtained with the same assay.
- plots include clearly labeled error bars for independent experiments and sample sizes. Unless justified, error bars should not be shown for technical replicates.
- if n<5, the individual data points from each experiment should be plotted. Any statistical test employed should be justified.
- Source Data should be included to report the data underlying figures according to the guidelines set out in the authorship guidelines on Data Presentation.

2. Captions
Each figure caption should contain the following information, for each panel where they are relevant:
- a specification of the experimental system investigated (eg cell line, species name).
- the assay(s) and method(s) used to carry out the reported observations and measurements.
- an explicit mention of the biological and chemical entity(ies) that are being measured.
- an explicit mention of the biological and chemical entity(ies) that are altered/varied/perturbed in a controlled manner.
- the exact sample size (n) for each experimental group/condition, given as a number, not a range;
- a description of the sample collection allowing the reader to understand whether the samples represent technical or biological replicates (including how many animals, litters, cultures, etc.).
- a statement of how many times the experiment shown was independently replicated in the laboratory.
- definitions of statistical methods and measures:
  - common tests, such as t-test (please specify whether paired vs. unpaired), simple χ2 tests, Wilcoxon and Mann-Whitney tests, can be unambiguously identified by name only, but more complex techniques should be described in the methods section;
  - are tests one-sided or two-sided?
  - are there adjustments for multiple comparisons?
  - exact statistical test results, e.g., P values = x but not P values < x;
  - definition of 'center values' as median or average;
  - definition of error bars as s.d. or s.e.m.

Please complete ALL of the questions below.
Select "Not Applicable" only when the requested information is not relevant for your study.

Materials

Newly Created Materials	Information included in the manuscript?	In which section is the information available? (Reagents and Tools Table, Materials and Methods, Figures, Data Availability Section)
New materials and reagents need to be available; do any restrictions apply?	Not Applicable	

Antibodies	Information included in the manuscript?	In which section is the information available? (Reagents and Tools Table, Materials and Methods, Figures, Data Availability Section)
For **antibodies** provide the following information:<br>- Commercial antibodies: RRID (if possible) or supplier name, catalogue number and or/clone number<br>- Non-commercial: RRID or citation	Yes	Methods

DNA and RNA sequences	Information included in the manuscript?	In which section is the information available? (Reagents and Tools Table, Materials and Methods, Figures, Data Availability Section)
**Short novel DNA or RNA including primers, probes**: provide the sequences.	Not Applicable	

Cell materials	Information included in the manuscript?	In which section is the information available? (Reagents and Tools Table, Materials and Methods, Figures, Data Availability Section)
**Cell lines:** Provide species information, strain. Provide accession number in repository **OR** supplier name, catalog number, clone number, and/**OR** RRID.	Yes	Methods
**Primary cultures:** Provide species, strain, sex of origin, genetic modification status.	Not Applicable	
Report if the cell lines were recently **authenticated** (e.g., by STR profiling) and tested for mycoplasma contamination.	Yes	Methods

Experimental animals	Information included in the manuscript?	In which section is the information available? (Reagents and Tools Table, Materials and Methods, Figures, Data Availability Section)
**Laboratory animals or Model organisms:** Provide species, strain, sex, age, genetic modification status. Provide accession number in repository **OR** supplier name, catalog number, clone number, **OR** RRID.	Yes	Methods
**Animal observed in or captured from the field:** Provide species, sex, and age where possible.	Not Applicable	
Please detail **housing and husbandry conditions**.	Not Applicable	

Plants and microbes	Information included in the manuscript?	In which section is the information available? (Reagents and Tools Table, Materials and Methods, Figures, Data Availability Section)
**Plants:** provide species and strain, ecotype and cultivar where relevant, unique accession number if available, and source (including location for collected wild specimens).	Not Applicable	
**Microbes:** provide species and strain, unique accession number if available, and source.	Not Applicable	

Human research participants	Information included in the manuscript?	In which section is the information available? (Reagents and Tools Table, Materials and Methods, Figures, Data Availability Section)
If collected and within the bounds of privacy constraints report on age, sex and gender or ethnicity for all study participants.	Not Applicable	

Core facilities	Information included in the manuscript?	In which section is the information available? (Reagents and Tools Table, Materials and Methods, Figures, Data Availability Section)
If your work benefited from core facilities, was their service mentioned in the acknowledgments section?	Not Applicable	

Design

Study protocol	Information included in the manuscript?	In which section is the information available? (Reagents and Tools Table, Materials and Methods, Figures, Data Availability Section)
If study protocol has been **pre-registered, provide DOI in the manuscript**. For clinical trials, provide the trial registration number **OR** cite DOI.	Not Applicable	
Report the **clinical trial registration number** (at ClinicalTrials.gov or equivalent), where applicable.	Not Applicable	

Laboratory protocol	Information included in the manuscript?	In which section is the information available? (Reagents and Tools Table, Materials and Methods, Figures, Data Availability Section)
Provide DOI OR other citation details if **external detailed step-by-step protocols** are available.	Not Applicable	

Experimental study design and statistics	Information included in the manuscript?	In which section is the information available? (Reagents and Tools Table, Materials and Methods, Figures, Data Availability Section)
Include a statement about **sample size** estimate even if no statistical methods were used.	Yes	Results and methods
Were any steps taken to minimize the effects of subjective bias when allocating animals/samples to treatment (e.g. **randomization procedure**)? If yes, have they been described?	Yes	Results and methods
Include a statement about **blinding** even if no blinding was done.	Yes	Results and methods
Describe **inclusion/exclusion criteria** if samples or animals were excluded from the analysis. Were the criteria pre-established?<br><br>If sample or data points were omitted from analysis, report if this was due to attrition or intentional exclusion and provide justification.	Yes	Results and methods
For every figure, are **statistical tests** justified as appropriate? Do the data meet the assumptions of the tests (e.g., normal distribution)? Describe any methods used to assess it. Is there an estimate of variation within each group of data? Is the variance similar between the groups that are being statistically compared?	Yes	Results and methods

Sample definition and in-laboratory replication	Information included in the manuscript?	In which section is the information available? (Reagents and Tools Table, Materials and Methods, Figures, Data Availability Section)
In the figure legends: state number of times the experiment was **replicated** in laboratory.	Yes	Figures
In the figure legends: define whether data describe **technical or biological replicates**.	Yes	Figures

Ethics

Ethics	Information included in the manuscript?	In which section is the information available? (Reagents and Tools Table, Materials and Methods, Figures, Data Availability Section)
Studies involving **human participants**: State details of **authority granting ethics approval** (IRB or equivalent committee(s), provide reference number for approval.	Not Applicable	
Studies involving **human participants**: Include a statement confirming that **informed consent** was obtained from all subjects and that the experiments conformed to the principles set out in the WMA Declaration of Helsinki and the Department of Health and Human Services Belmont Report.	Not Applicable	
Studies involving **human participants:** For publication of **patient photos**, include a statement confirming that consent to publish was obtained.	Not Applicable	
Studies involving experimental **animals**: State details of **authority granting ethics approva**l (IRB or equivalent committee(s), provide reference number for approval. Include a statement of compliance with ethical regulations.	Not Applicable	
Studies involving **specimen and field samples:** State if relevant **permits** obtained, provide details of authority approving study; if none were required, explain why.	Not Applicable	

Dual Use Research of Concern (DURC)	Information included in the manuscript?	In which section is the information available? (Reagents and Tools Table, Materials and Methods, Figures, Data Availability Section)
Could your study fall under dual use research restrictions? Please check biosecurity documents and list of **select agents and toxins** (CDC): https://www.selectagents.gov/sat/list.htm	Not Applicable	
If you used a select agent, is the security level of the lab appropriate and reported in the manuscript?	Not Applicable	
If a study is subject to dual use research of concern regulations, is the name of the **authority granting approval and reference number** for the regulatory approval provided in the manuscript?	Not Applicable	

Reporting

The MDAR framework recommends adoption of discipline-specific guidelines, established and endorsed through community initiatives. Journals have their own policy about requiring specific guidelines and recommendations to complement MDAR.

Adherence to community standards	Information included in the manuscript?	In which section is the information available? (Reagents and Tools Table, Materials and Methods, Figures, Data Availability Section)
State if relevant guidelines or checklists (e.g., **ICMJE, MIBBI, ARRIVE, PRISMA**) have been followed or provided.	Not Applicable	
For **tumor marker prognostic studies,** we recommend that you follow the **REMARK** reporting guidelines (see link list at top right). See author guidelines, under 'Reporting Guidelines'. Please confirm you have followed these guidelines.	Not Applicable	
For **phase II and III randomized controlled trials**, please refer to the **CONSORT** flow diagram (see link list at top right) and submit the CONSORT checklist (see link list at top right) with your submission. See author guidelines, under 'Reporting Guidelines'. Please confirm you have submitted this list.	Not Applicable	

Data Availability

Data availability	Information included in the manuscript?	In which section is the information available? (Reagents and Tools Table, Materials and Methods, Figures, Data Availability Section)
Have **primary datasets** been deposited according to the journal's guidelines (see 'Data Deposition' section) and the respective accession numbers provided in the Data Availability Section?	Not Applicable	
Were **human clinical and genomic datasets** deposited in a public access-controlled repository in accordance to ethical obligations to the patients and to the applicable consent agreement?	Not Applicable	
Are **computational models** that are central and integral to a study available without restrictions in a machine-readable form? Were the relevant accession numbers or links provided?	Not Applicable	
If publicly available data were reused, provide the respective **data citations in the reference list**.	Not Applicable	
